# Supplementary material for: Behaviour-based dependency networks between places shape urban economic resilience
Source: Nat Hum Behav. 2024 Dec 23;9(3):496–506. doi: 10.1038/s41562-024-02072-7 (PMC11936834; doi:10.1038/s41562-024-02072-7)
Supplement: Supplementary file 1 — Supplementary Notes 1–7, Figs. 1–41 and Tables 1–52. [file 41562_2024_2072_MOESM1_ESM.pdf]

nature human behaviour

Supplementary information https://doi.org/10.1038/s41562-024-02072-7

Behaviour-based dependency networks between places shape urban economic resilience

In the format provided by the authors and unedited

Supplementary Notes

1 Mobility data analytics and representativeness **7**

1.1 Home estimation and stop detection 7
1.2 Safegraph POI data and visit attribution 8
1.3 Data representativeness 9
1.4 Post-stratification of mobility data 9

2 Behavior-based dependency networks **15**

2.1 Measuring behavior-based dependency between places 15
2.2 Qualitative characteristics of networks 15
2.3 Comparison of networks under different co-visit detection parameters 16

3 Statistical robustness of behavior-based dependency networks **30**

3.1 Robustness against choice of time period 30
3.2 Computing quartiles of w_{ij} via bootstrap method 30
3.3 Robustness against post-stratification processing 32

4 Modeling dependency network weights **38**

4.1 Gravity-based null networks 38
4.2 Regression model of w_{ij} 39
4.3 Robustness against different network instances 39
4.4 Robustness against co-visit detection parameters 44
4.5 Robustness against different time periods 46

5 Impacts of behavior-based dependency during the COVID-19 pandemic **49**

5.1 Analysis of visitation losses in cities 49
5.2 Model specification and estimation results 49
5.3 Robustness against co-visit detection parameters 52
5.4 Results using changes in visits during different time periods 57
5.5 Results on post-pandemic recovery using different time periods 57
5.6 Robustness of regression results when using different time periods to generate dependency networks 63
5.7 Robustness of regression results when using only short non-work stays 64
5.8 Case study on school closure period 65

6 Cascading impacts of hypothetical shocks **81**

6.1 Leontief open model 81
6.2 Cascading effects of individual place closures 81

7 Software **89**

List of Figures

1 Estimates of number of visits to POIs under different maximum spatial threshold parameters . . . 11
2 Sample rates of mobile phone location data across the five metropolitan areas. 12
3 Comparison of census population and smartphone users for census block groups in the five metropolitan areas . . . 13
4 Comparison of census block group median income and the sample rate of mobile phone smartphone users in the five metropolitan areas . . . 14
5 Distribution of time and step differences between two POIs visited by the same individual on the same day . . . 16
6 POI subcategories with the largest average in- and out-weights . . . 17
7 Proportion of edge weights among POI category pairs . . . 19
8 POI subcategories with the largest average in- and out-weights, when $T_c = 1$ hour . . 20
9 POI subcategories with the largest average in- and out-weights, when $T_c = 3$ hour . . 21
10 Dependency network in New York under different co-visit detection parameters . . . 22
11 Dependency network in Boston under different co-visit detection parameters 23
12 Dependency network in Seattle under different co-visit detection parameters 24
13 Dependency network in Los Angeles under different co-visit detection parameters . . 25
14 Dependency network in Dallas under different co-visit detection parameters 26
15 Network diagram showing the average dependencies between POI subcategories in New York and Boston . . . 27
16 Network diagram showing the average dependencies between POI subcategories in Seattle and Los Angeles . . . 28
17 Network diagram showing the average dependencies between POI subcategories in Dallas . . . 29
18 Comparison of in- and out-weights of each POI across different data collection periods 31
19 Comparison of in- and out-weights of each POI across different data collection periods, contd. . . . 32
20 Comparison of category pairwise weight proportion across different data collection periods . . . 33
21 Comparison of category pairwise weight proportion across different data collection periods . . . 34
22 Out- and in-weights of POIs when applying bootstrap method to remove smaller weights 35
23 Out- and in-weights of categories when applying bootstrap method to remove insignificant weights . . . 36
24 Out- and in-weights of POIs when applying post-stratification to correct for biases in mobile phone data . . . 37
25 Correlation between the observed n_{ij} values and the gravity component g_{ij} under different model parameters . . . 38
26 Comparison of actual and null networks in New York and Boston . . . 40
27 Comparison of actual and null networks in Seattle and Los Angeles . . . 41
28 Comparison of actual and null networks in Dallas . . . 42
29 Comparison between real and null network on the proportion of edge weights among POI category pairs . . . 42
30 Histograms of edge distances between actual and null networks. . . . 43

31 Regression results for dependency weights when using different co-visit detection parameters, contd. 45
32 Change in visits to POIs in New York, Boston, Seattle, Los Angeles, and Dallas during the pandemic period. 50
33 Correlation between change in visits of ego and the weighted change in visits of alters by category pairs 51
34 Regression results for dependency weights when using different co-visit detection parameters, contd. 56
35 Change in visits to POIs in New York, Boston, Seattle, Los Angeles, and Dallas during the fall semester period. 66
36 Cascading impacts of a 50% visit reduction to colleges. 82
37 Cascading impacts of a 100% visit reduction to colleges. 83
38 Cascading impacts of a 25% visit reduction to colleges. 84
39 Total cascading impact of closing places on other locations in New York and Boston . 86
40 Total cascading impact of closing places on other locations in Seattle and Los Angeles 87
41 Total cascading impact of closing places on other locations in Dallas 88

List of Tables

1 Number of places in the Safegraph dataset in the five core-based statistical areas (CBSAs) analyzed in this study. 8
2 Description of the four core-based statistical areas (CBSAs) analyzed in this study. . 10
3 Linear regression models predicting the logged dependency weights between POIs i and j ($log_{10}w_{ij}$) using physical factors 44
4 Linear regression models predicting the logged dependency weights between POIs i and j ($log_{10}w_{ij}$) selected using the bootstrap method with physical factors 46
5 Linear regression models predicting the logged dependency weights between POIs i and j ($log_{10}w_{ij}$) selected using only short stays ($<$ 4 hrs) with physical factors . . . 47
6 Linear regression models predicting the logged dependency weights between POIs i and j ($log_{10}w_{ij}$) using network from 2019 January - April data 47
7 Linear regression models predicting the logged dependency weights between POIs i and j ($log_{10}w_{ij}$) selected using network from 2019 May - August data 48
8 Linear regression models predicting the change in visits to POIs during the pandemic (2019 March - May) in New York 53
9 Linear regression models predicting the change in visits to POIs during the pandemic (2019 March - May) in Boston 54
10 Linear regression models predicting the change in visits to POIs during the pandemic (2019 March - May) in Seattle 54
11 Linear regression models predicting the change in visits to POIs during the pandemic (2019 March - May) in Los Angeles 55
12 Linear regression models predicting the change in visits to POIs during the pandemic (2019 March - May) in Dallas 55
13 Linear regression models predicting the change in visits to POIs during the pandemic (2019 June - August) in New York 58

14 Linear regression models predicting the change in visits to POIs during the pandemic (2019 June - August) in Boston 58
15 Linear regression models predicting the change in visits to POIs during the pandemic (2019 June - August) in Seattle 59
16 Linear regression models predicting the change in visits to POIs during the pandemic (2019 June - August) in Los Angeles 59
17 Linear regression models predicting the change in visits to POIs during the pandemic (2019 June - August) in Dallas 60
18 Linear regression models predicting the change in visits to POIs during the pandemic (2019 September - November) in New York 60
19 Linear regression models predicting the change in visits to POIs during the pandemic (2019 September - November) in Boston 61
20 Linear regression models predicting the change in visits to POIs during the pandemic (2019 September - November) in Seattle 61
21 Linear regression models predicting the change in visits to POIs during the pandemic (2019 September - November) in Los Angeles 62
22 Linear regression models predicting the change in visits to POIs during the pandemic (2019 September - November) in Dallas 62
23 Linear regression models predicting the recovery of visits to POIs during the pandemic (2020 June - August) in New York compared to the initial stages of the pandemic (2020 March - May) 63
24 Linear regression models predicting the recovery of visits to POIs during the pandemic (2020 June - August) in Boston compared to the initial stages of the pandemic (2020 March - May) 64
25 Linear regression models predicting the recovery of visits to POIs during the pandemic (2020 June - August) in Seattle compared to the initial stages of the pandemic (2020 March - May) 65
26 Linear regression models predicting the recovery of visits to POIs during the pandemic (2020 June - August) in Los Angeles compared to the initial stages of the pandemic (2020 March - May) 67
27 Linear regression models predicting the recovery of visits to POIs during the pandemic (2020 June - August) in Dallas compared to the initial stages of the pandemic (2020 March - May) 68
28 Linear regression models predicting the recovery of visits to POIs during the pandemic (2020 September - November) in New York compared to the initial stages of the pandemic (2020 March - May) 68
29 Linear regression models predicting the recovery of visits to POIs during the pandemic (2020 September - November) in Boston compared to the initial stages of the pandemic (2020 March - May) 69
30 Linear regression models predicting the recovery of visits to POIs during the pandemic (2020 September - November) in Seattle compared to the initial stages of the pandemic (2020 March - May) 69
31 Linear regression models predicting the recovery of visits to POIs during the pandemic (2020 September - November) in Los Angeles compared to the initial stages of the pandemic (2020 March - May) 70

32 Linear regression models predicting the recovery of visits to POIs during the pandemic (2020 September - November) in Dallas compared to the initial stages of the pandemic (2020 March - May) 70
33 Linear regression models predicting the change in visits to POIs during the pandemic (2019 September - November) in New York when using dependency network generated from data between 2019 January to April 71
34 Linear regression models predicting the change in visits to POIs during the pandemic (2019 September - November) in Boston when using dependency network generated from data between 2019 January to April 71
35 Linear regression models predicting the change in visits to POIs during the pandemic (2019 September - November) in Seattle when using dependency network generated from data between 2019 January to April 72
36 Linear regression models predicting the change in visits to POIs during the pandemic (2019 September - November) in Los Angeles when using dependency network generated from data between 2019 January to April 72
37 Linear regression models predicting the change in visits to POIs during the pandemic (2019 September - November) in Dallas when using dependency network generated from data between 2019 January to April 73
38 Linear regression models predicting the change in visits to POIs during the pandemic (2019 September - November) in New York when using dependency network generated from data between 2019 May to August 73
39 Linear regression models predicting the change in visits to POIs during the pandemic (2019 September - November) in Boston when using dependency network generated from data between 2019 May to August 74
40 Linear regression models predicting the change in visits to POIs during the pandemic (2019 September - November) in Seattle when using dependency network generated from data between 2019 May to August 74
41 Linear regression models predicting the change in visits to POIs during the pandemic (2019 September - November) in Los Angeles when using dependency network generated from data between 2019 May to August 75
42 Linear regression models predicting the change in visits to POIs during the pandemic (2019 September - November) in Dallas when using dependency network generated from data between 2019 May to August 75
43 Linear regression models predicting the change in visits to POIs during the pandemic (2019 September - November) in New York when using dependency network generated from stays shorter than 4 hours 76
44 Linear regression models predicting the change in visits to POIs during the pandemic (2019 September - November) in Boston when using dependency network generated from stays shorter than 4 hours 76
45 Linear regression models predicting the change in visits to POIs during the pandemic (2019 September - November) in Seattle when using dependency network generated from stays shorter than 4 hours 77
46 Linear regression models predicting the change in visits to POIs during the pandemic (2019 September - November) in Los Angeles when using dependency network generated from stays shorter than 4 hours 77

47 Linear regression models predicting the change in visits to POIs during the pandemic (2019 September - November) in Dallas when using dependency network generated from stays shorter than 4 hours . . . 78
48 Linear regression models predicting the change in visits to POIs during the school semester (2019 September - November) compared to summer break (2019 June - August) in New York . . . 78
49 Linear regression models predicting the change in visits to POIs during the school semester (2019 September - November) compared to summer break (2019 June - August) in Boston . . . 79
50 Linear regression models predicting the change in visits to POIs during the school semester (2019 September - November) compared to summer break (2019 June - August) in Seattle . . . 79
51 Linear regression models predicting the change in visits to POIs during the school semester (2019 September - November) compared to summer break (2019 June - August) in Los Angeles . . . 80
52 Linear regression models predicting the change in visits to POIs during the school semester (2019 September - November) compared to summer break (2019 June - August) in Dallas . . . 80

1 Mobility data analytics and representativeness

1.1 Home estimation and stop detection

In this study, we utilize an anonymized location dataset of mobile phones and smartphone devices provided by Spectus Inc., a location data intelligence company which collects anonymous, privacy-compliant location data of mobile devices using their software development kit (SDK) technology in mobile applications and ironclad privacy framework. Spectus processes data collected from mobile devices whose owners have actively opted in to share their location and requires all application partners to disclose their relationship with Spectus, directly or by category, in the privacy policy. With this commitment to privacy, the data set contains location data for roughly 15 million daily active users in the United States. Through Spectus' Data for Good program, Spectus provides mobility insights for academic research and humanitarian initiatives. All data analyzed in this study are aggregated to preserve privacy[1]. Each entry in the data table comprises anonymized device ID, location coordinates, start time, and dwell time of the stop for the device.

To define the type of location (Home or Work), different variables are used, including the number of days spent in a given location in the last month, the daily average number of hours spent in that location, and the time of the day spent in the location (nighttime/daytime). To estimate the home position of a user, the algorithm combines the three variables and creates a score that represents the probability that the position points to the home. The more days and the average number of hours spent in the position, the higher the score is. Higher scores will also be assigned to the most common places during the night. The location that maximizes this score is defined as the home of the device.

Once the location of the home location is identified, the algorithm looks for the work position. Note that the algorithm requires the work location to be located at least 100 meters apart from the home location. The same variables used for the detection of the home location are used, but a higher score is given to daytime locations for the work location rather than nighttime locations. Spectus runs the algorithm every week in order to confirm or update the inferred home and work locations as we observe new data. We will only consider devices that have been present in Spectus' dataset for at least 15 days. Spectus tightly restricts access to the inferred precise home and work locations of devices. Furthermore, it is used as input into various downstream processes to create more privacy-protected versions of Spectus datasets. For example, we only expose home and work datasets in Spectus Workbench associated with standard Census Block Groups, created by the U.S. Census Bureau, rather than the precise locations. This offers a good balance between utility and privacy: according to the U.S. Census Bureau, there are between 600 and 3000 people living in each block group. Each block group is an aggregate of contiguous U.S. blocks sharing similar socio-demographic characteristics. The representativeness of this data has been tested and corrected in Section 1.3 in the Supplementary Material. The stops, which are location clusters where individual users stay for a given duration, are estimated using the Sequence Oriented Clustering approach [17].

In this study, we selected New York, Boston, Seattle, Los Angeles, and Dallas as the five CBSAs given constraints on data collection. The five cities were selected with respect to the diversity of characteristics in terms of geographical locations (2 Northeast, 1 Northwest, 1 Southwest, and 1 Southern), sociodemographic details (population ranges from 3.5M in Seattle to 19.8M in New York), political inclinations, and weather characteristics.

[1] `https://spectus.ai/privacy/privacy-policy/`

Supplementary Table 1: Number of places in the Safegraph dataset in the five core-based statistical areas (CBSAs) analyzed in this study.

	CBSAs				
Place category	New York	Boston	Seattle	Los Angeles	Dallas
Arts and Museums	4,580	1,183	799	3,697	1,073
City and Outdoors	7,201	5,311	2,792	4,817	2,174
Coffee and Tea	14,799	3,481	3,392	11,395	4,360
College	3,737	1,266	766	2,661	942
Entertainment	2,296	657	584	2,101	745
Food	66,348	12,687	9,924	39,907	16,795
Grocery	14,763	2,942	1,964	7,636	3,792
Health	20,654	4,799	3,913	14,212	5,806
Office	7,840	3,256	2,628	10,936	6,128
Service	114,581	33,525	23,920	104,253	45,520
Shopping	92,211	20,302	14,089	79,024	29,069
Sports	12,383	5,892	2,464	10,509	3,782
Transportation	41,729	11,114	13,245	32,141	14,056
All places	403,669	107,081	81,140	326,332	136,686

1.2 Safegraph POI data and visit attribution

To measure the visitation patterns of individuals in urban environments, we attribute the stops of individual users to specific places in the city. To study the stops at different places, we use stops that are longer than 10 minutes but shorter than ten hours. In our study, we use location data of places collected by Safegraph [2]. To protect the users' privacy, we have removed various privacy-sensitive places from our places database. Sensitive places include health-related places, places where the vulnerable population are located, military-related, religious facilities, places that are related to sexual-orientation, and adult-oriented places [3]. As a result, we have a total of 403,669 places in New York, 107,081 places in Boston, 81,140 places in Seattle, 326,332 places in Los Angeles, and 136,686 places in Dallas. The breakdown of the number of places by the place category is shown in Table 1. To attribute a stop to a place, we simply attribute each stop the closest place in our dataset. To avoid attributing a stop to place far away, we attribute the stop to a place within $d_{max} = 100$ meters from the observed location of the stop. If the stop is further away than 100 meters from any place in the dataset, the stop is discarded from our dataset and not used for computing the diversity of encounters.

The robustness of our estimation of the number of visits to different locations has been tested using different spatial thresholds of d_{max}. Different levels of d_{max} could change how individuals' stays are attributed to the places and thus could affect our estimates of the income diversity of physical encounters. Figure 1 compares the estimated number of visits to places over a 1 month period in the five cities when we use different levels of d_{max} (y-axis) with our default parameter $d_{max} = 100m$ (x-axis). For all values $d_{max} = \{50, 150, 200\}$, the Pearson correlation of the number of visits are extremely high, in most cases above $\rho > 0.95$. This robustness check shows that the estimated

[2] `https://www.safegraph.com/`

[3] `https://spectus.ai/privacy/spoi-policy/`

visitation patterns do not depend on the choice of the spatial threshold parameter for visit attribution.

1.3 Data representativeness

The location data used in our study is collected from smartphones via various apps and services. Although a significant portion (85% according to 2021 data[4]) of the US population owns a smartphone, one could question the representativeness of the 1.16 million user samples across geographical regions and income quantiles. Studies have reported the digital divide and smartphone usage gaps across sociodemographic groups in the US [14]. In this section, we test whether our group of users in the mobility data is representative of the total population, and further employ post-stratification techniques to correct for any potential biases in the sampling rates across places and socioeconomic status and to test whether the results on income diversity dynamics are robust to such uncertainties concerning data representativeness.

The sampling percentage of the mobility data (100% $\times$ number of observed mobile phone users divided by the total population from the census data) is around 5% to 10% across all census block groups (CBGs) in the metropolitan regions, as shown in Figure 2. Sample rates are calculated by dividing the total number of users who were observed across a three-month period in the dataset by the census block group population obtained from the American Community Survey [2]. There is a large variation in the sample rates, within and across metropolitan areas. To test whether the users in the location data are representative of the entire population, first, we compare the population detection in our mobility data and the 2019 ACS data for each of the CBGs in the cities. The panels in Figure 3 show the comparison between the census population (x-axis) and the number of observed smartphone users (y-axis) on the CBG scale in the months of September 2019 to January 2020 in the five metropolitan areas. The correlation is moderately high, between around $\rho = 0.55$ and $\rho = 0.78$, showing that despite the use of such small census areas and potential bias in the smartphone usage patterns, we are able to obtain a good representation of the population. To overcome this bias, in Section 1.4, we use post-stratification techniques to correct for such differences in the sample percentages across CBGs. Later, in Section 3.3, we assess whether our estimates of visitation patterns are affected by the representativeness of the data.

In addition to the differences in sampling rates across CBGs, differences in representativeness across income groups are important to ensure the findings of the study are not biased toward specific sociodemographic groups. To measure the representativeness across income quantiles, we plot the correlation between the median income of the census block group and the sample rate (shown in Figure 2) for the five metropolitan areas, as shown in Figure 4. A balanced dataset would have a nonsignificant correlation between the two metrics. However, we observe a small (below $\rho = 0.2$) but significant positive correlation between income and sample rates for New York, Los Angeles, and Dallas. This indicates that in these metropolitan areas, higher income groups are over-represented in the dataset. In Section 1.4, we use post-stratification techniques to correct for such differences in the sample percentages across CBGs and income groups.

1.4 Post-stratification of mobility data

To correct for, and to understand the effects of the varying sampling rates across CBGs and income groups on our estimation of visitation patterns to places, we apply a post-stratification technique,

[4]https://www.statista.com/topics/2711/us-smartphone-market/#topicHeader__wrapper

Supplementary Table 2: Description of the four core-based statistical areas (CBSAs) analyzed in this study.

CBSA	Population	# users (monthly)	# stays (monthly)	# places
New York-Newark-Jersey City	19.95M	1,456K	36.67M	403,669
Boston-Cambridge-Newton	4.64M	144K	2.34M	107,081
Seattle-Tacoma-Bellevue	3.55M	141K	2.23M	81,140
Los Angeles-Long Beach-Anaheim	13.05M	452K	10.00M	326,332
Dallas-Fort Worth-Arlington	6.70M	425K	8.83M	136,686
Total	47.89M	2.62M	60.07M	1,054,908

which is used in previous studies [10, 18]. Post-stratification is a well know sampling tool [11] and is typically used to study the impact of sampling biases in mobile phone location data [6] or (geolocated) social media data [16] on various downstream tasks and analyses. Following the methods employed in Moro et al. [10], we denote w_g the expansion factor, which is the ratio of the population of census block group g to the population detected in our mobility data. We then weight the visits completed by people from census block group g spends at place α by

$$\hat{n}_{g\alpha} = w_g n_{g\alpha}$$

so that the number of visits are inversely weighted by the representativeness of the mobile phone users in census block group g. Using this method, we could increase (decrease) the visits to places by people coming from census block groups that are under-estimated (over-estimated).

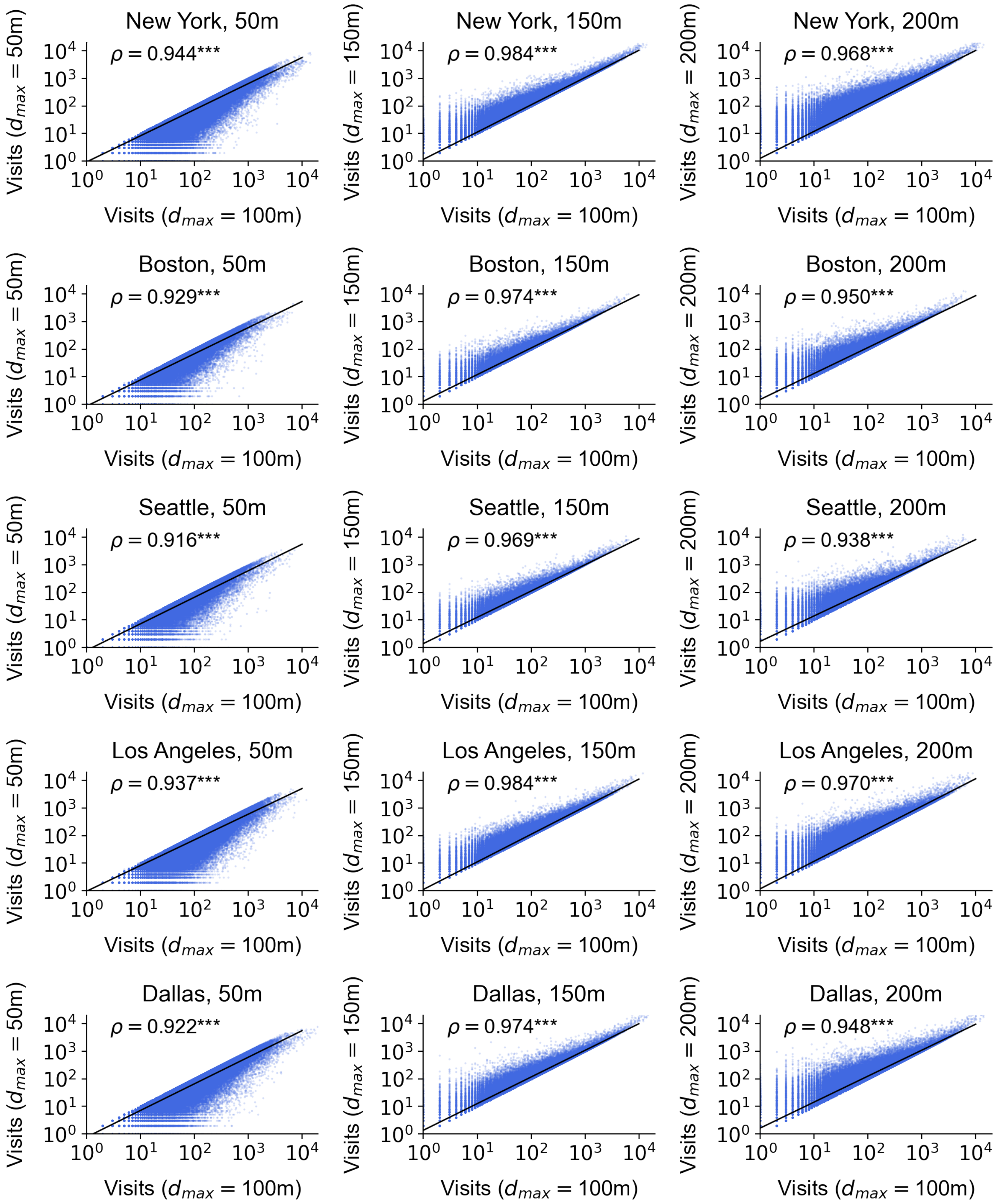

Supplementary Figure 1: Estimates of number of visits to POIs under different maximum spatial threshold parameters. Pearson correlation of estimated number of visits to different POIs between different spatial threshold parameters are extremely high across all cities.

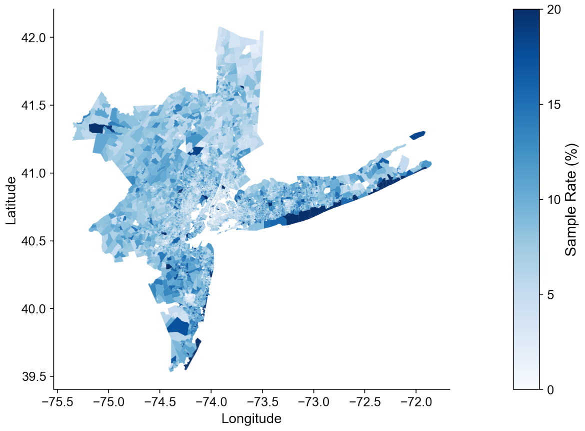

(a) New York

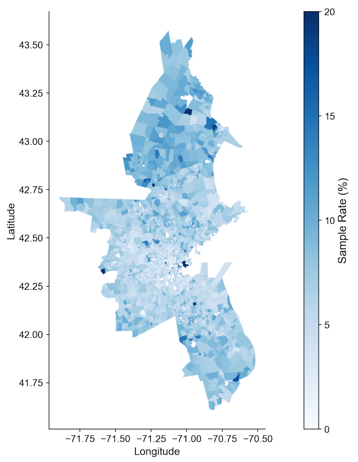

(b) Boston

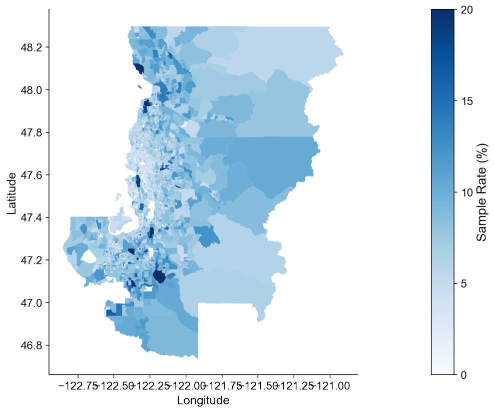

(c) Seattle

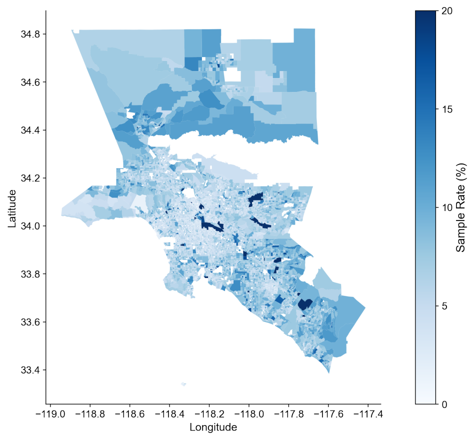

(d) Los Angeles

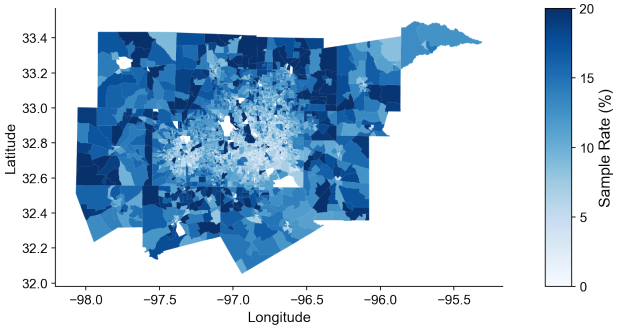

(e) Dallas

Supplementary Figure 2: Sample rates of mobile phone location data across the five metropolitan areas. Sample rates are calculated by dividing the total number of users who were observed across a three month period in the dataset by the census block group population obtained from the American Community Survey [2]. Maps were produced in Python using the TIGER shapefiles from the U.S. Census Bureau [15]

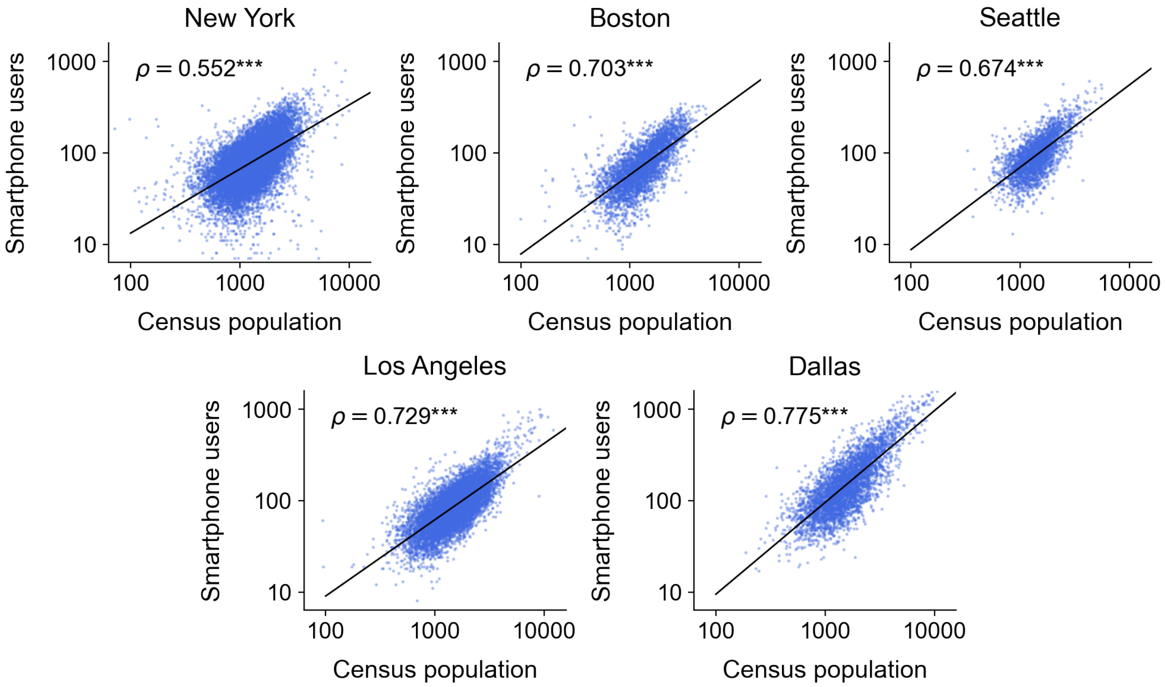

Supplementary Figure 3: Comparison of census population and smartphone users for census block groups in the five metropolitan areas. The correlation is moderately high, between around $\rho = 0.55$ and $\rho = 0.78$, showing that despite the use of such small census areas and potential bias in the smartphone usage patterns, we are able to obtain a good representation of the population. This bias is corrected using post-stratification techniques.

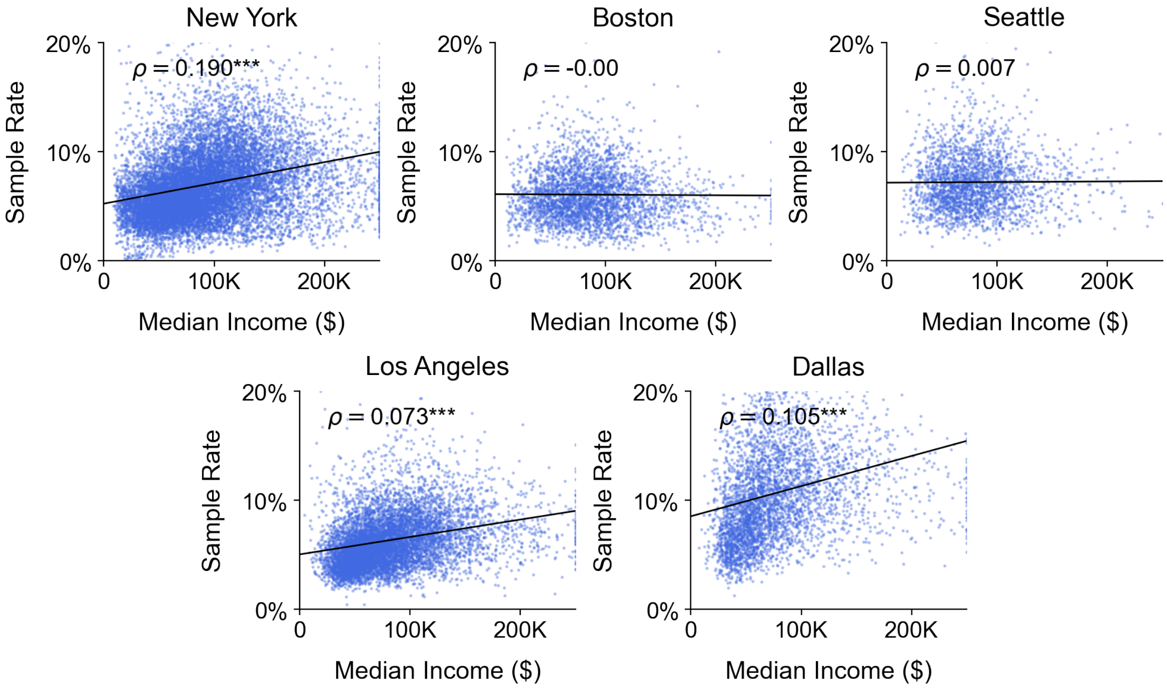

Supplementary Figure 4: Comparison of census block group median income and the sample rate of mobile phone smartphone users in the five metropolitan areas. The correlation is small (below $\rho = 0.2$) but significantly positive in New York, Los Angeles, and Dallas. This indicates that in these metropolitan areas, higher income groups are over-represented in the dataset. This bias is corrected using post-stratification techniques.

2 Behavior-based dependency networks

2.1 Measuring behavior-based dependency between places

Using the large and longitudinal dataset of GPS location records in five major US metropolitan areas introduced in Supplementary Note 1, we construct the behavior-based dependency network at the level of points-of-interest (POIs; e.g., businesses and amenities) in cities. Within the mobility dataset, we identified stays at places that were detected to be between 10 minutes and 10 hours and we spatially matched those stays with the closest place locations within 100 meters to infer visits to specific POIs, as described in Supplementary Note 1. The representativeness of the data across regions and income levels was ensured via post-stratification techniques.

In this study, the dependence of a POI i on another POI j is defined as

$$w_{ij} = \frac{n_{ij}}{n_i} \tag{1}$$

where n_i denotes the number of visits to POI i and n_{ij} denotes the number of 'co-visits' between POIs i and j. A co-visit is defined as an instance in which POIs i and j were visited by the same individual:

- on the same day,
- within T_c hours from exiting POI i (j) to entering POI j (i), and
- within T_s intermediate POIs

Because the denominator is based on the number of visits to the target POI, $w_{ij} \neq w_{ji}$. This simple but intuitive measure considers the asymmetric nature of dependencies between POIs. As shown in Figure 5, the distributions of the time difference or number of intermediate steps between any given two POIs within a movement trajectory follow a long-tailed distribution, where the majority of the pairs of POIs can be included within 6 hours and 1 step difference. Therefore, as a baseline parameter setting, we use $T_c = 6$ hours and $T_s = 1$ POIs (which indicates direct visitation from POI i to POI j, or vice-versa). The characteristics of the dependency network when using different co-visit detection threshold parameters T_c and T_s are tested and discussed in the following sections such as Supplementary Note 2.3 and 4.2. By computing the dependency weights w_{ij} $\forall i, j$, we obtain the behavior-based dependency matrix $W \in \mathbb{R}^{N \times N}$ where N is the total number of POIs present in the CBSA.

2.2 Qualitative characteristics of networks

To obtain a better understanding of the generated behavior-based dependency network, the POI subcategories with the largest average in- and out- weights were plotted in Figure 6. Supercenters, malls, department malls, and airports had the largest in-weights, indicating that these places were depended by many other POIs. On the other hand, places such as art dealers and various types of retail stores had the largest average out-weight, indicating that such places had high dependency on other places. The subcategories that appeared in both rankings were consistent across the five metropolitan areas. As shown in Figures 8 and 9, the POI subcategories that appeared in each ranking were similar when we changed the co-visit detection parameter to $T_c = 1$ and $T_c = 3$, respectively.

To further observe the dependency relationships between categories, we computed the proportion of edge weights among POI category pairs, shown in Figure 7. The proportion of edge weights was

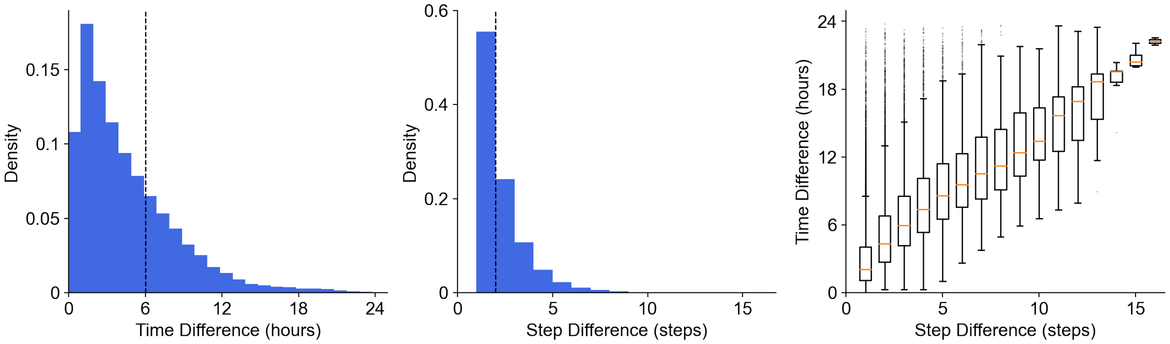

Supplementary Figure 5: Distribution of time and step differences between two POIs visited by the same individual on the same day. The majority of the co-visits can be captured by using a threshold of 6 hours time difference and 1 step difference. The dependency networks were computed using co-visits that occurred within 6 hours and 1 step. The right panel shows the box plots of time difference in hours for each step difference value. The box extends from the first quartile (Q1) to the third quartile (Q3) of the data, with a line at the median. The whiskers extend from the box to the farthest data point lying within 1.5x the inter-quartile range (IQR) from the box. Flier points are those past the end of the whiskers.

computed by taking the sum of weights that connect the vertical and horizontal categories and dividing that by the total weights that exist in the network, more formally, by $\sum_{i \in A, j \in B} w_{ij} / \sum_{i,j} w_{ij}$. As the matrices in Figure 7 show, the patterns of dependency are consistent across cities, with Food, Service, Shopping, and Transport playing a big role in both depending on and being depended by others.

2.3 Comparison of networks under different co-visit detection parameters

How much do the co-visit detection parameters (T_s,T_c) affect the structural properties of the dependency networks? To investigate this, we generated the dependency network using 25 pairs of parameters ($T_s = [1, 2, 3, 5, unlimited]$ and $T_c = [1, 3, 6, 12, 24]$ hours). The following analysis compares the following characteristics of the dependency networks across different co-vist detection parameters:

- POI subcategories with largest in- and out-weights (Figures 8 and 9)
- Distributions of in-weights and out-weights per POI (panels (a) and (b) in Figures 10 to 14)
- Relationship between average weight and physical distance between the POIs (panels (a) and (b) in Figures 10 to 14)
- Difference in the proportion of weights for each category pair (positive means baseline network has more weights between the category pairs) (panel (c) in Figures 10 to 14)

As shown in Figures 8 and 9, the POI subcategories that appeared in each ranking were similar when we changed the co-visit detection parameter to $T_c = 1$ and $T_c = 3$, respectively. Under all parameters, supercenters, malls, department malls, and airports had the largest in-weights, while places such as art dealers and various types of retail stores had the largest average out-weight.

Panels (a) in Figures 10 to 14) show that although the distribution obviously shifts to the right (more steps would include more links, thus more in- and out- weights) the maximum step difference

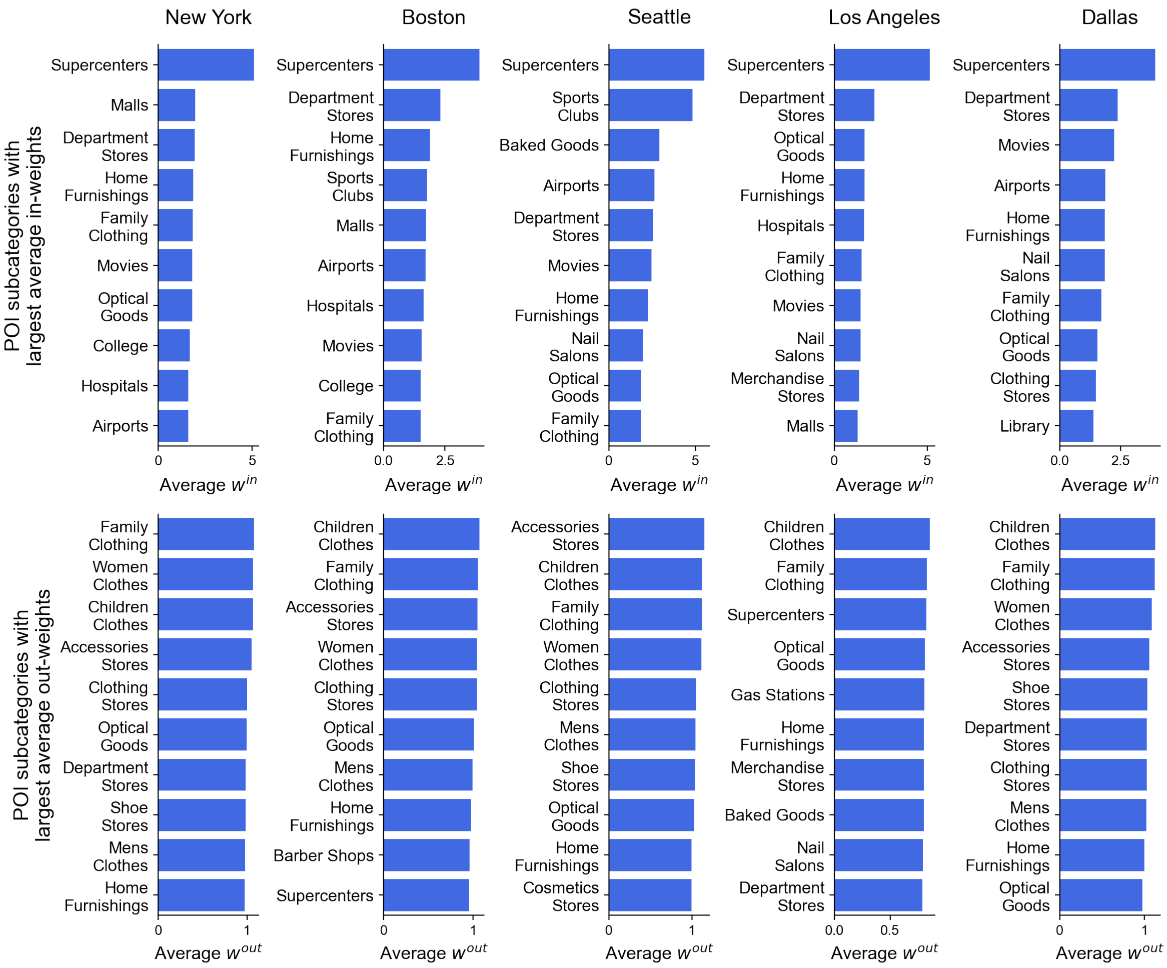

Supplementary Figure 6: POI subcategories with the largest average in- and out-weights Supercenters, malls, department malls, and airports had the largest in-weights, while places such as art dealers and various types of retail stores had the largest average out-weight.

parameter does not make a big difference on the distributions of the in-weights, out-weights, or the decay of average weights with the physical distance.

On the other hand, Panels (b) in Figures 10 to 14) show that the distributions are significantly affected by the choice of time difference parameter. Obviously, the longer the temporal threshold we use, the distributions shift to the right (more time difference would include more links, thus more in- and out- weights). Especially, the dependency networks with the 1 hour threshold present a significantly sparser network with lower in-weights, out-weights, and faster decay of distance with respect to physical distance. The $T_c = 6$ threshold that we chose as the baseline parameter generates network characteristics that are closely in between $T_c = 3$, and $T_c = 12$ and $T_c = 24$.

Panels (c) in Figures 10 to 14) show that despite the changes in co-visit detection parameters, the proportion of weights among category pairs are not affected significantly. Note that positive differences (blue color) mean that the baseline network ($T_s = 1$,$T_c = 6$) contains more weight in the specific category pair. The only exception is $T_c = 1$ (similar to the in-weights, out-weights, and

distance decay curves), where we observe much fewer weights among shopping POIs in the baseline network. This indicates that when we limit the time difference to 1 hour, we capture significantly more of the shopping-to-shopping weights compared to other category pairs.

Network diagrams in Figures 15 to 17 show the average dependencies between POI subcategories in the five cities. Each node represents a POI subcategory (there are 96 of them in the dataset) and the three largest outgoing dependency edges are shown for each node. Node sizes show the in-degree of the constructed network (i.e., how many other POI categories depend on that node). Many shopping subcategories including supercenters, department stores, malls, and clothing stores, and colleges, cafes, restaurants are depended by many other subcategories.

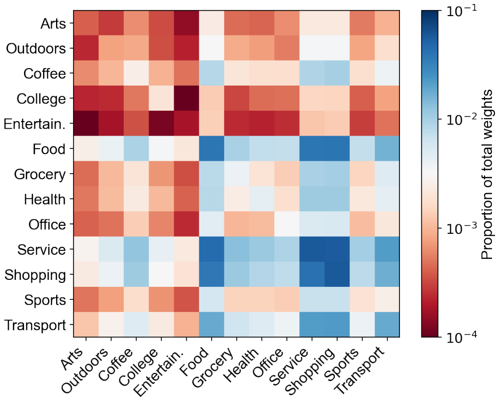

(a) New York

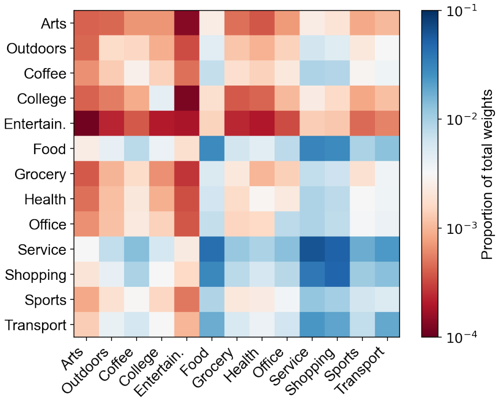

(b) Boston

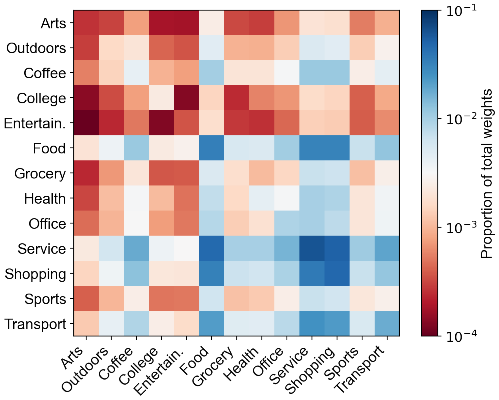

(c) Seattle

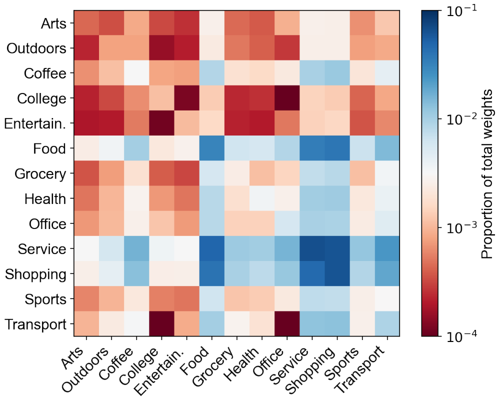

(d) Los Angeles

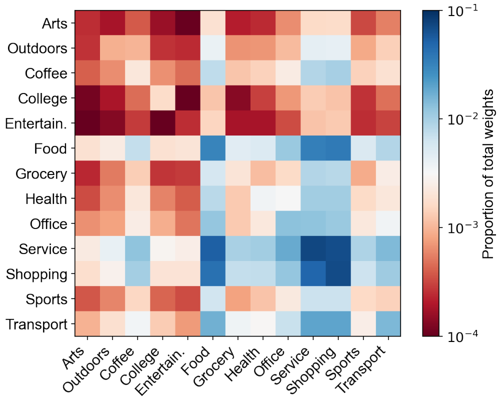

(e) Dallas

Supplementary Figure 7: Proportion of edge weights among POI category pairs. The proportion of edge weights was computed by taking the sum of weights that connect the vertical and horizontal categories and dividing that by the total weights that exist in the network. The patterns of dependency are consistent across cities, with Food, Service, Shopping, and Transport playing a big role in both depending on and being depended by others.

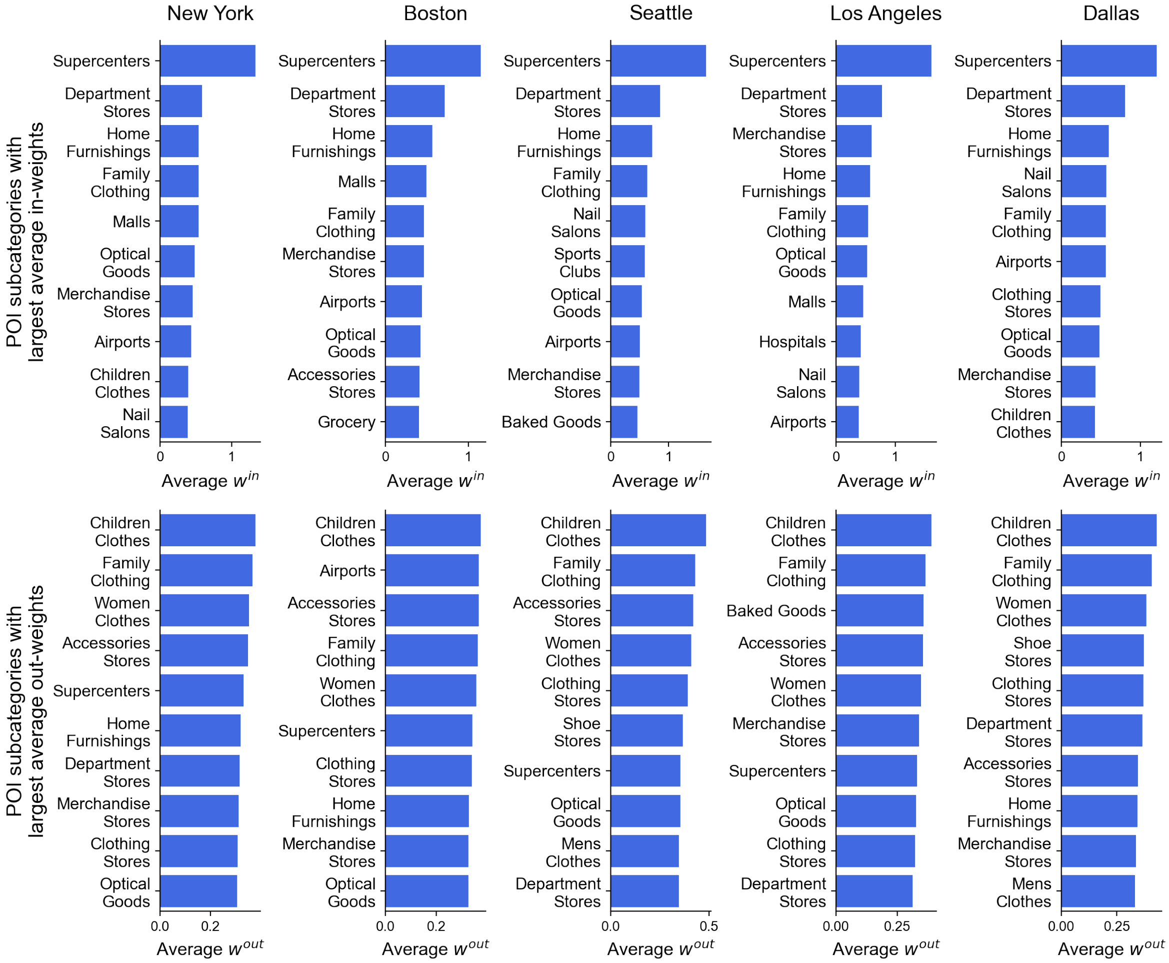

Supplementary Figure 8: POI subcategories with the largest average in- and out-weights, when $T_c = 1$ hour Supercenters, malls, department malls, and airports had the largest in-weights, while places such as art dealers and various types of retail stores had the largest average out-weight. Results were similar to the baseline parameter when $T_c = 6$ hours.

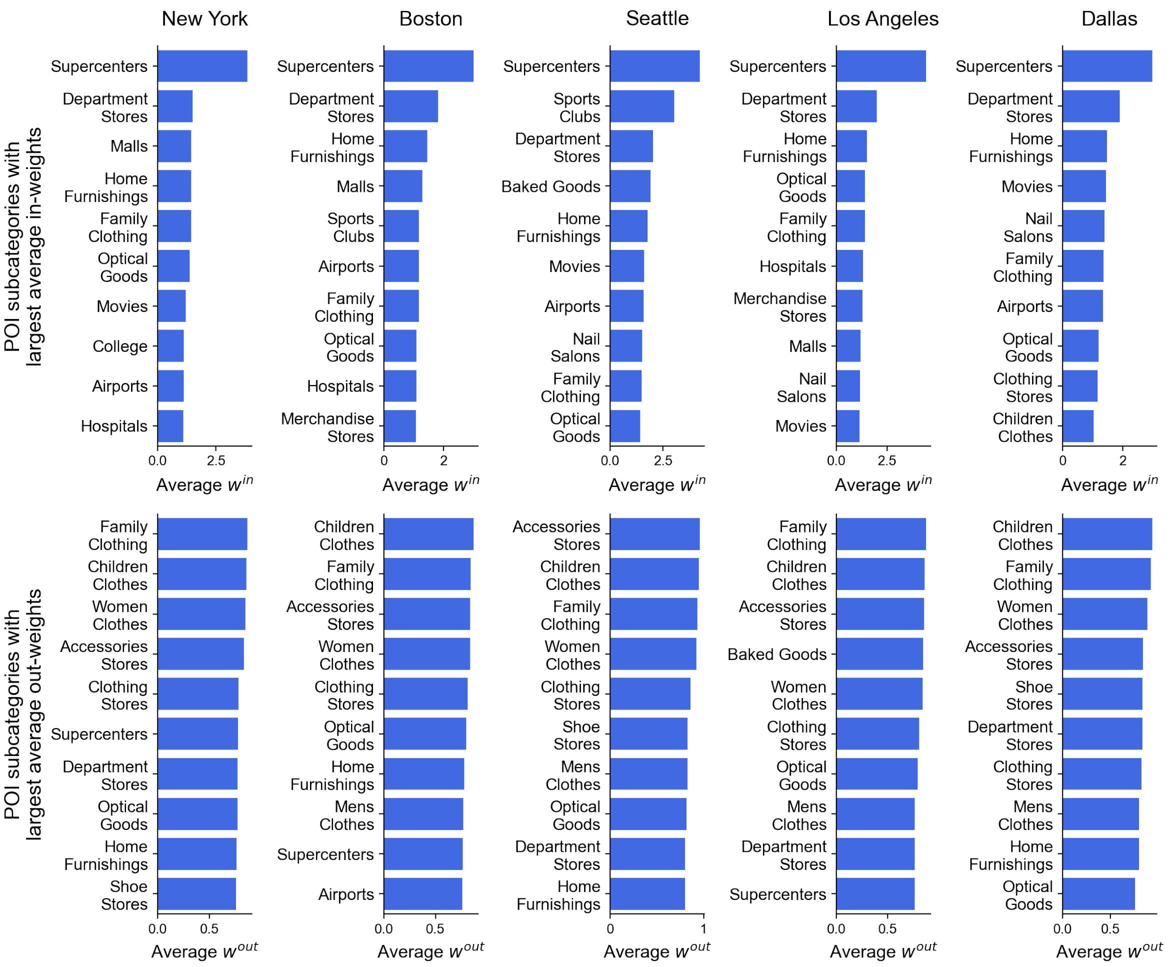

Supplementary Figure 9: POI subcategories with the largest average in- and out-weights, when $T_c = 3$ hour Supercenters, malls, department malls, and airports had the largest in-weights, while places such as art dealers and various types of retail stores had the largest average out-weight. Results were similar to the baseline parameter when $T_c = 6$ hours.

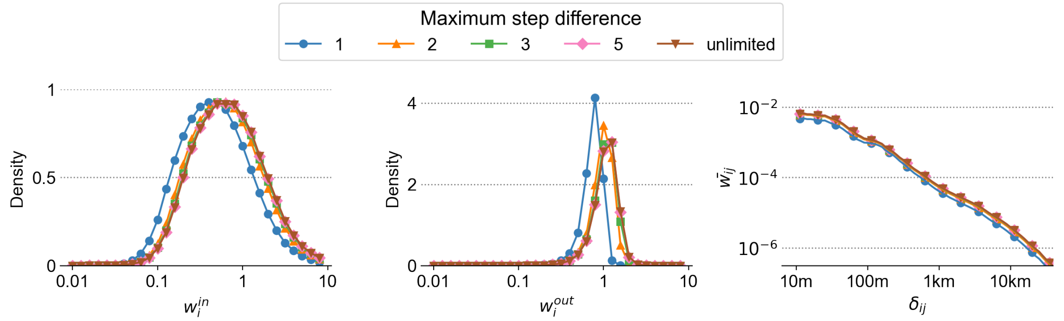

(a) Network characteristics under different maximum step difference T_s

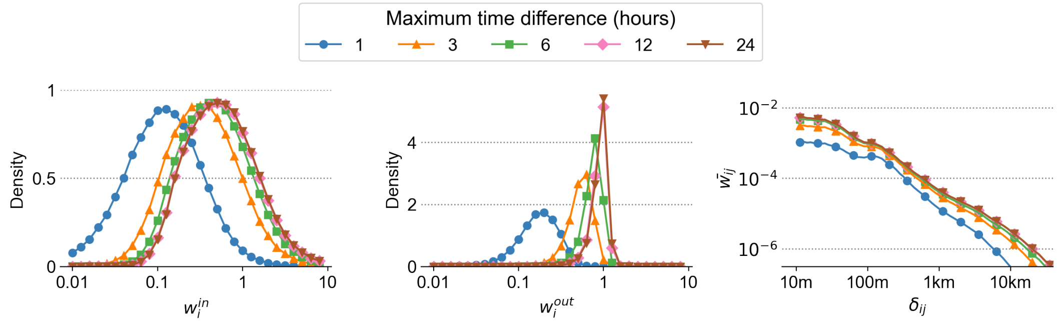

(b) Network characteristics under different maximum time difference T_c

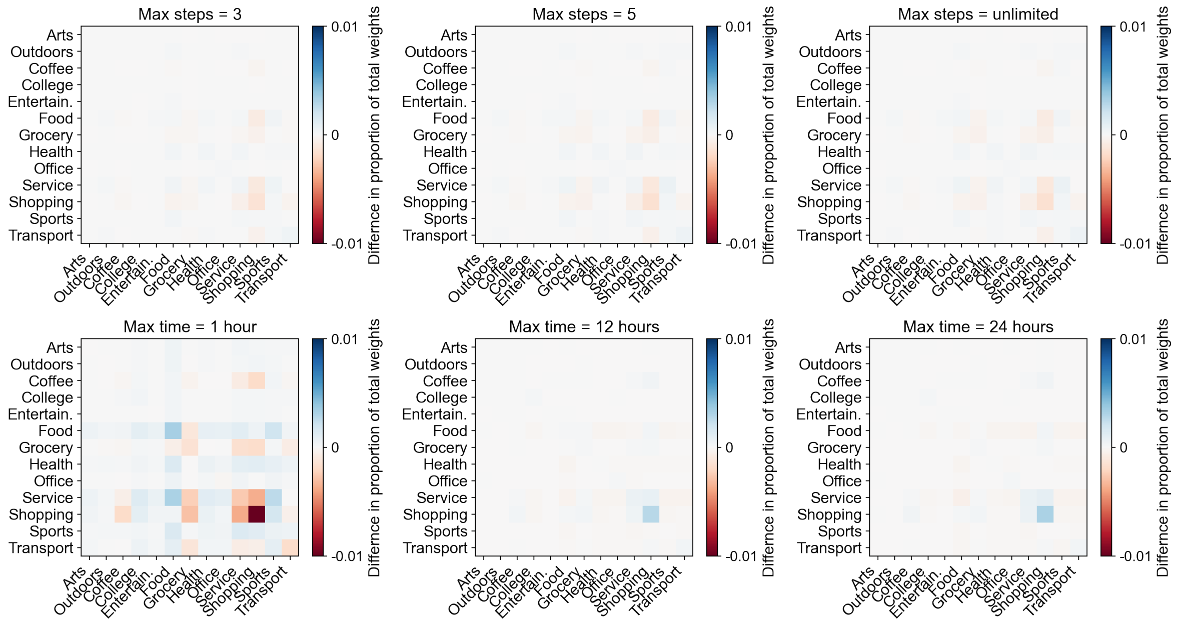

(c) Difference in category pairwise dependency weights compared to baseline network

Supplementary Figure 10: Dependency network in New York under different co-visit detection parameters.

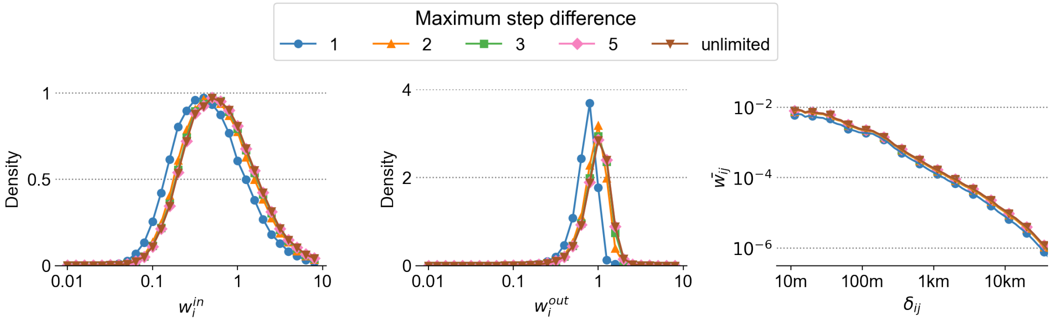

(a) Network characteristics under different maximum step difference T_s

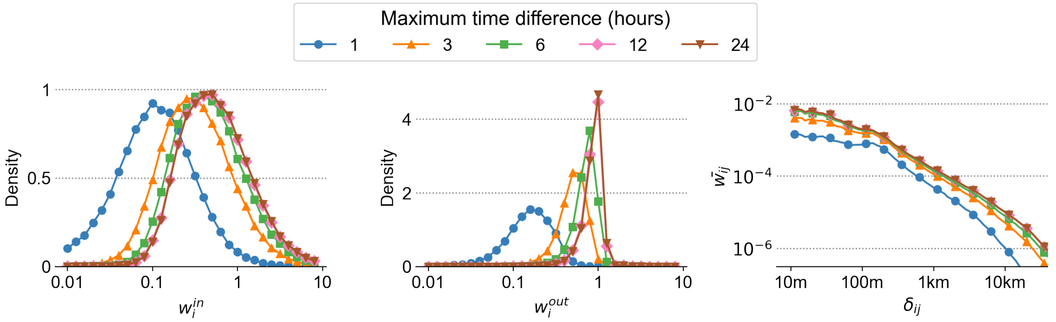

(b) Network characteristics under different maximum time difference T_c

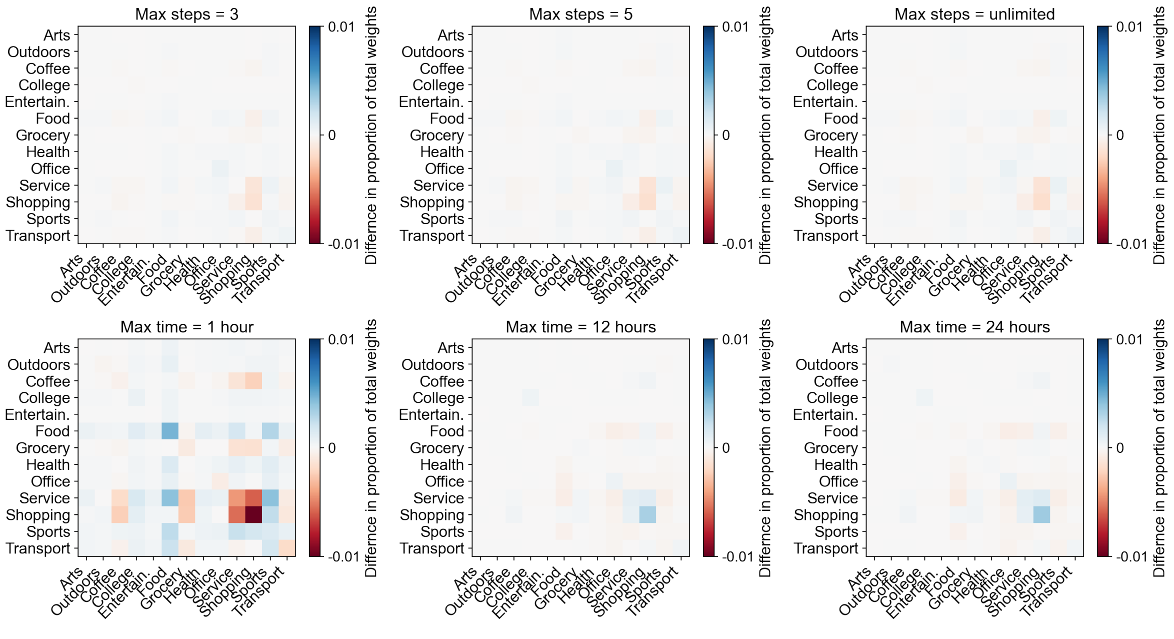

(c) Difference in category pairwise dependency weights compared to baseline network

Supplementary Figure 11: Dependency network in Boston under different co-visit detection parameters.

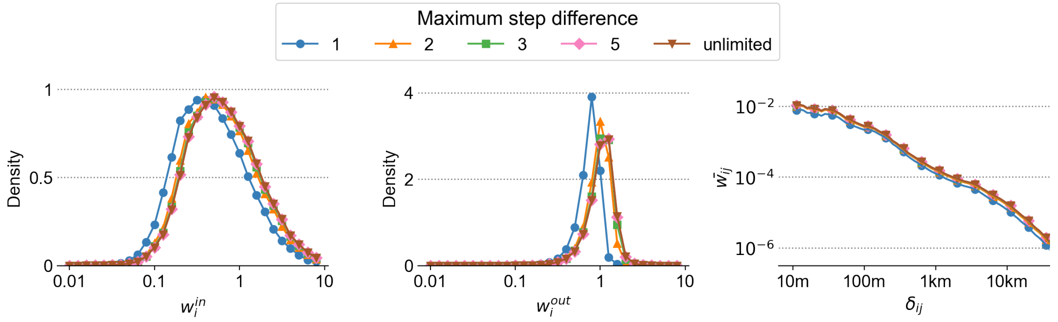

(a) Network characteristics under different maximum step difference T_s

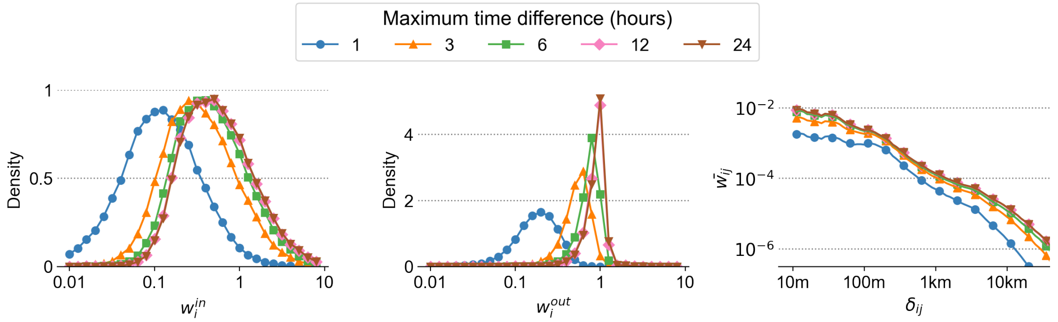

(b) Network characteristics under different maximum time difference T_c

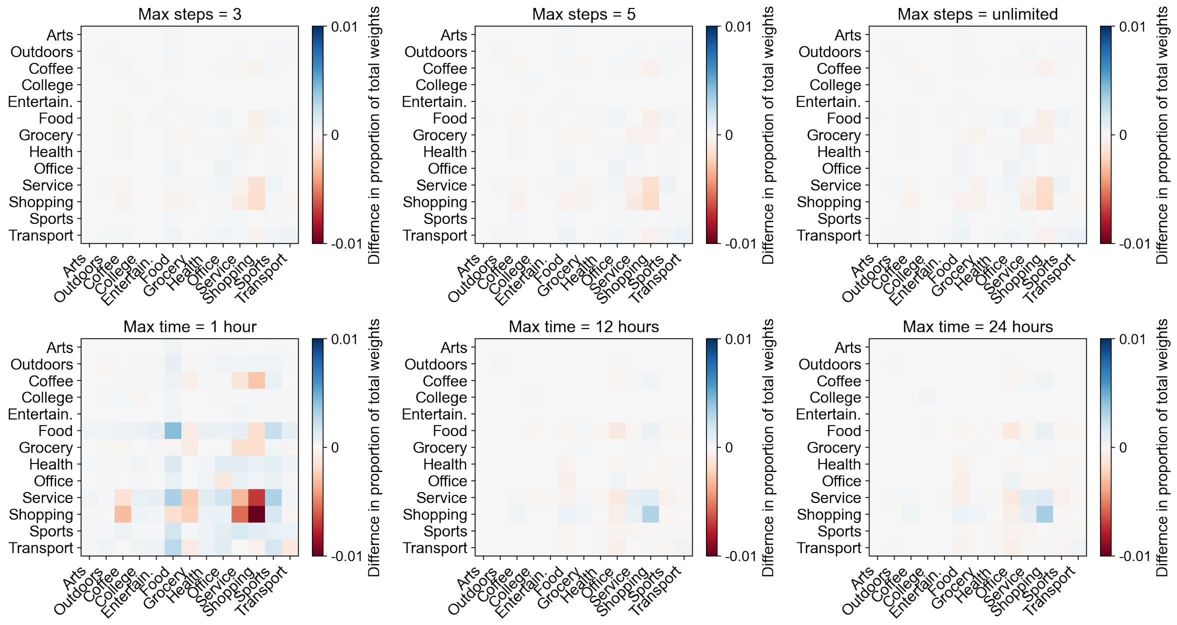

(c) Difference in category pairwise dependency weights compared to baseline network

Supplementary Figure 12: Dependency network in Seattle under different co-visit detection parameters.

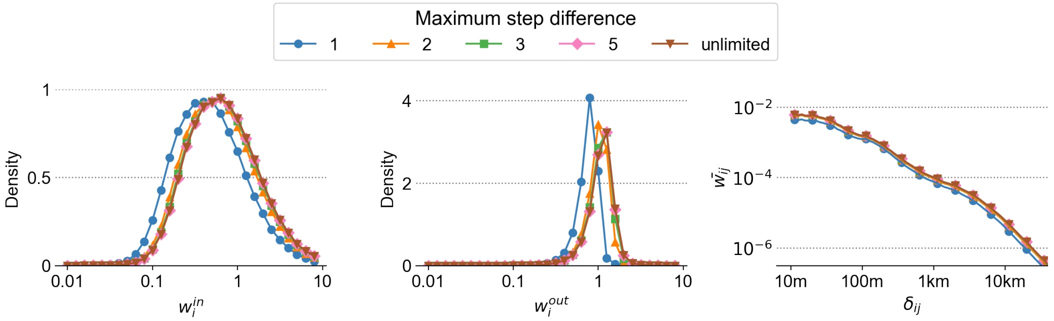

(a) Network characteristics under different maximum step difference T_s

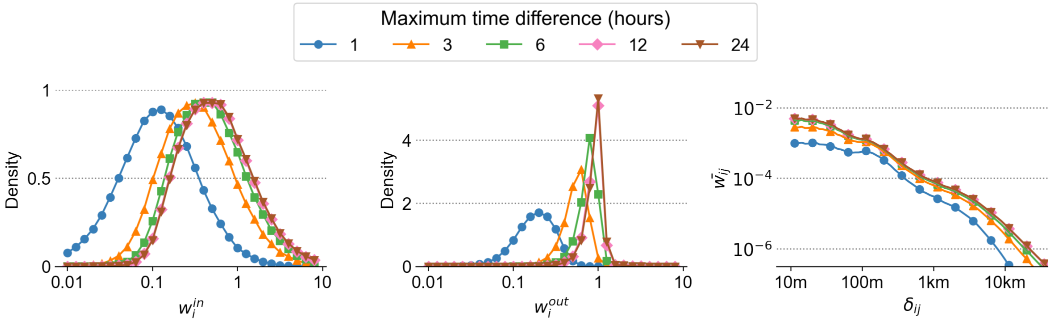

(b) Network characteristics under different maximum time difference T_c

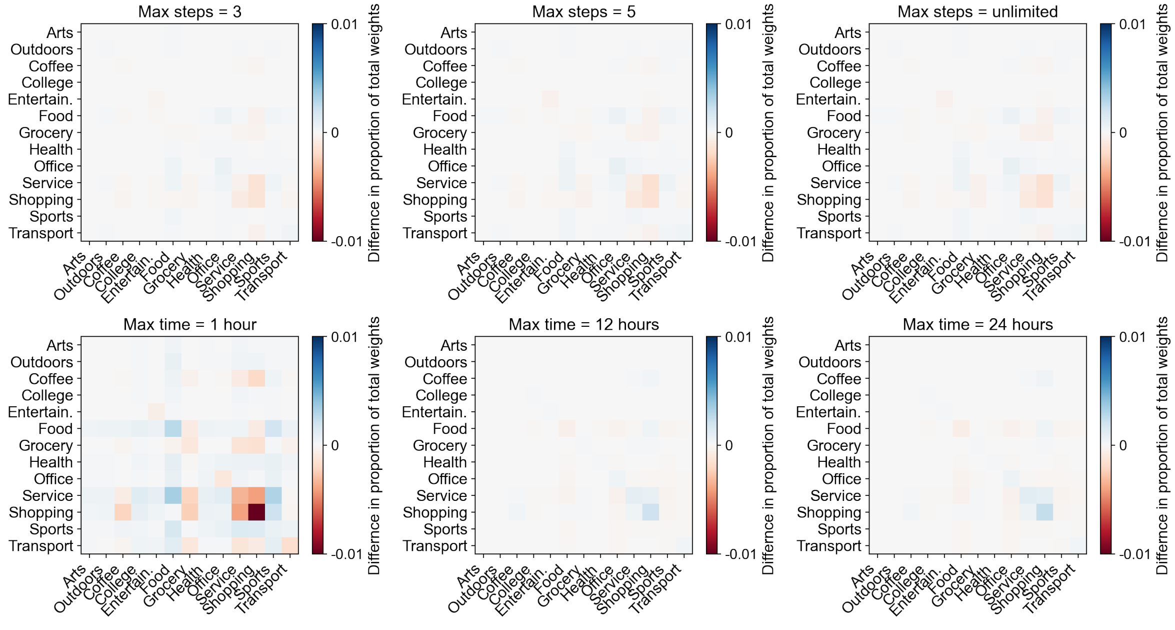

(c) Difference in category pairwise dependency weights compared to baseline network

Supplementary Figure 13: Dependency network in Los Angeles under different co-visit detection parameters.

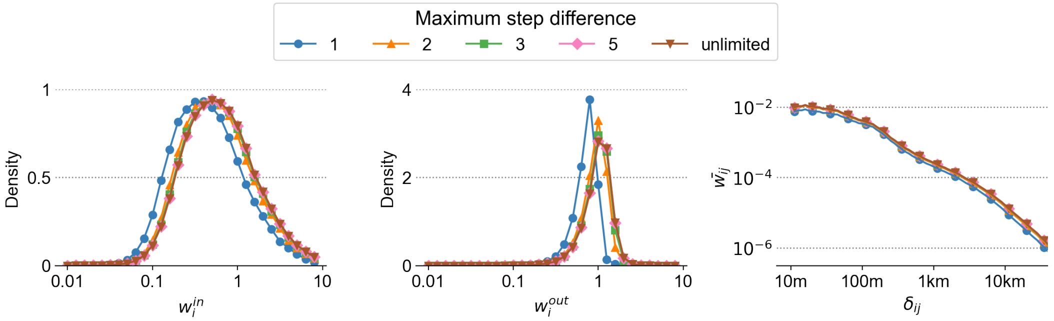

(a) Network characteristics under different maximum step difference T_s

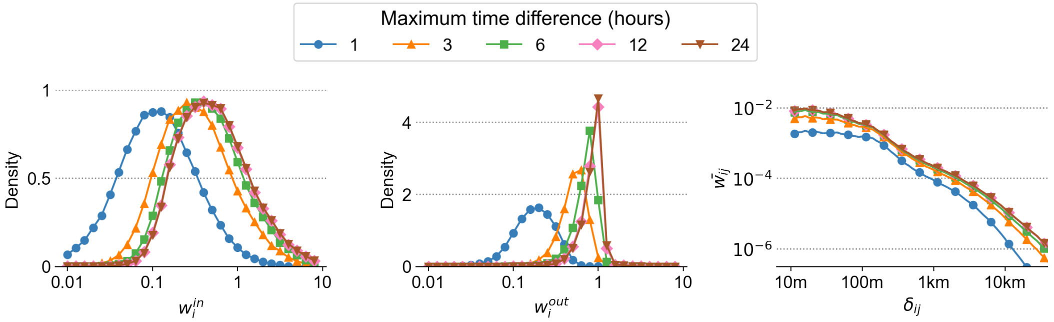

(b) Network characteristics under different maximum time difference T_c

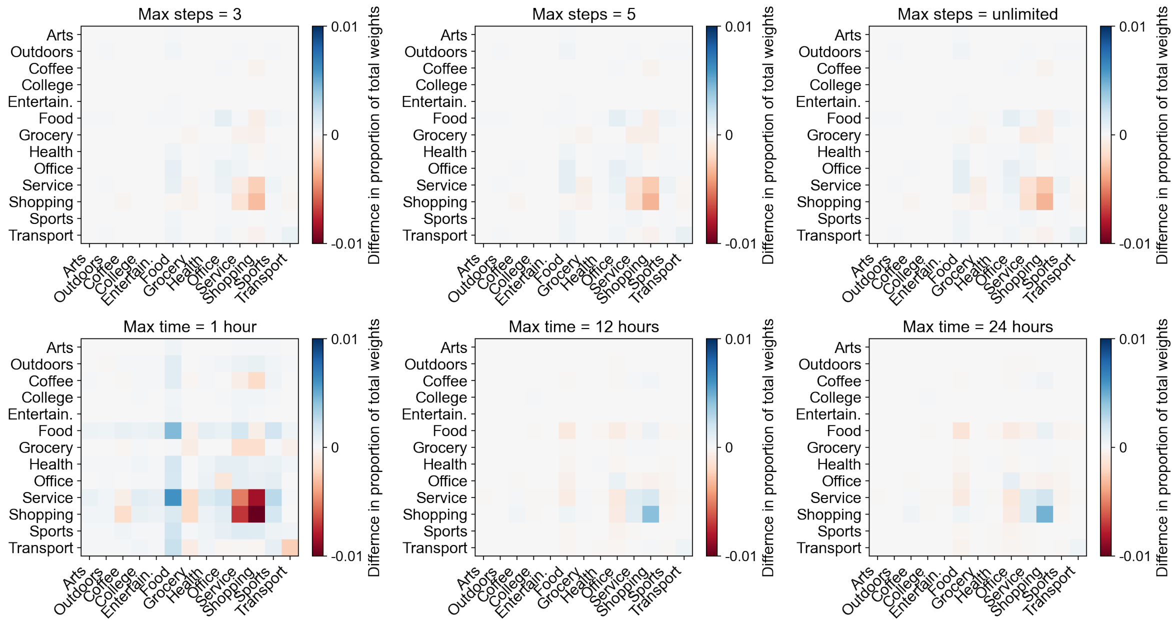

(c) Difference in category pairwise dependency weights compared to baseline network

Supplementary Figure 14: Dependency network in Dallas under different co-visit detection parameters.

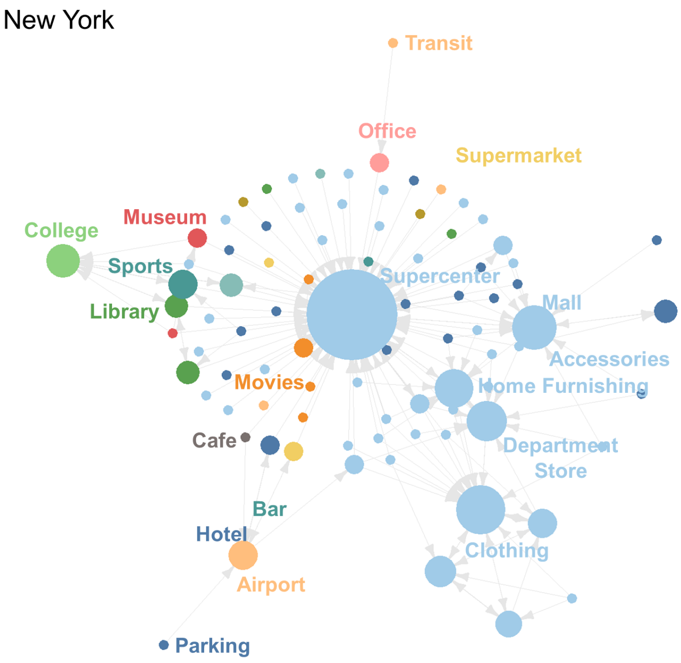

(a) New York

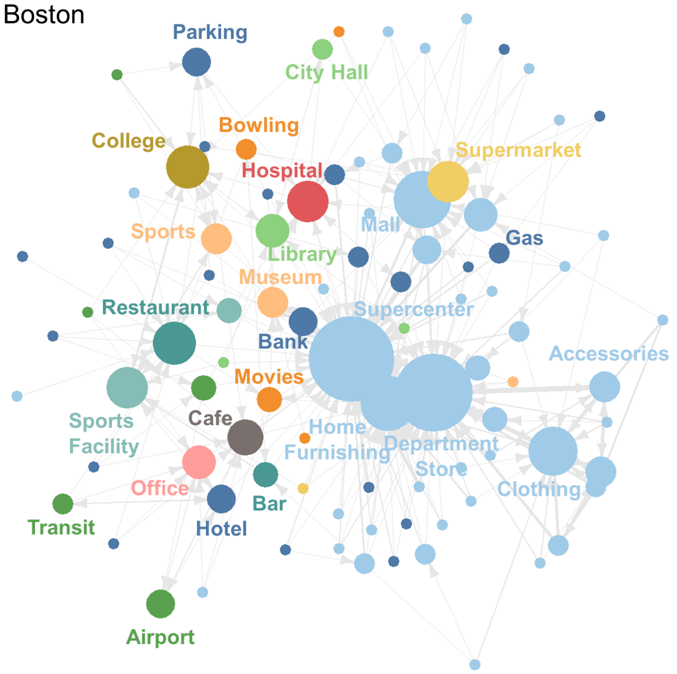

(b) Boston

Supplementary Figure 15: Network diagram showing the average dependencies between POI subcategories in New York and Boston. Each node is a POI subcategory and the three largest outgoing dependency edges are shown for each node. Node sizes show the in-degree of the constructed network.

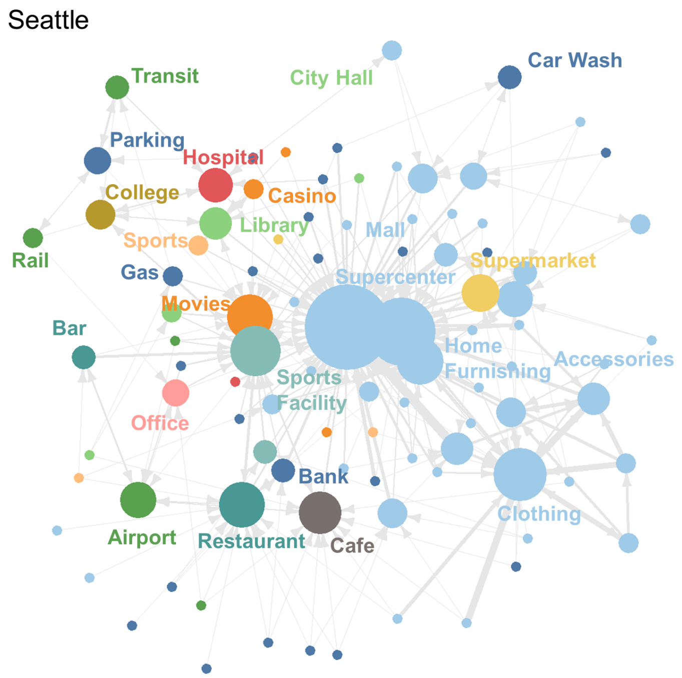

(a) Seattle

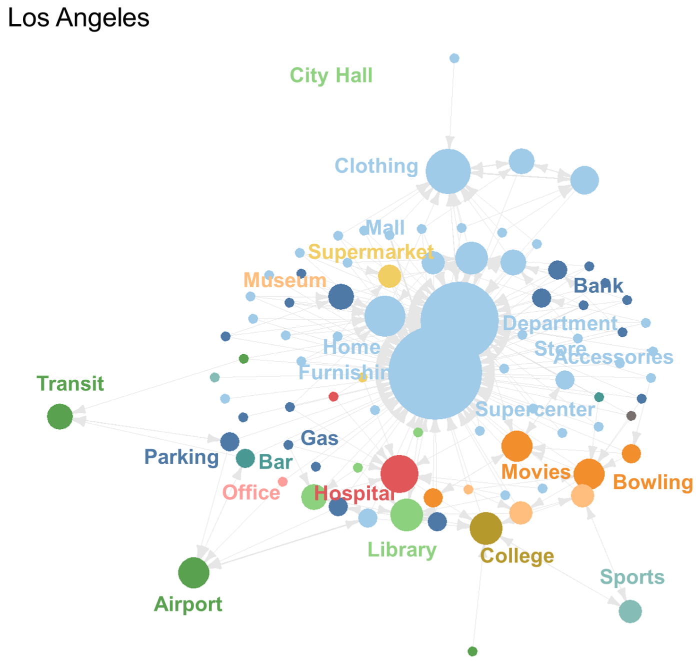

(b) Los Angeles

Supplementary Figure 16: Network diagram showing the average dependencies between POI subcategories in Seattle and Los Angeles. Each node is a POI subcategory and the three largest outgoing dependency edges are shown for each node. Node sizes show the in-degree of the constructed network.

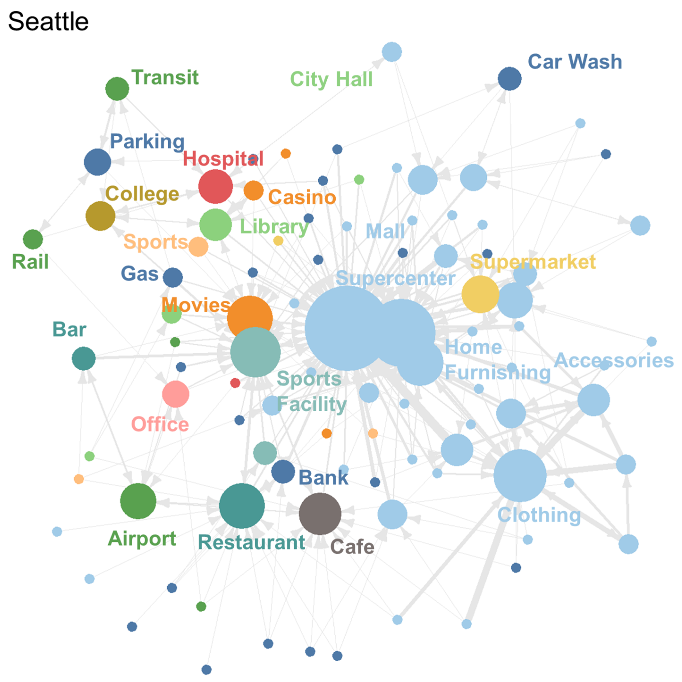

(a) Dallas

Supplementary Figure 17: Network diagram showing the average dependencies between POI subcategories in Dallas. Each node is a POI subcategory and the three largest outgoing dependency edges are shown for each node. Node sizes show the in-degree of the constructed network.

3 Statistical robustness of behavior-based dependency networks

Apart from co-visit detection parameters T_c and T_s, many factors and decision parameters could affect the characteristics of the dependency network and its weights. In this section, we test the statistical robustness of the edge weights and network characteristics by comparing the dependency network with several variants. More specifically, we will make comparisons with the following networks:

- Dependency network computed using data from a different time period, for example, January – April 2019, instead of September – December 2019.
- Dependency network where edges with small weights are removed. This is done by computing the 25th percentile of the weights w_{ij} using bootstrap method.
- Dependency network calculated without the post-stratification procedure.

The following subsections show the details of how each type of network is calculated and comparisons of network statistics with the empirical dependency network. In this section, comparisons of edge-based and category-aggregated edge weights are compared between the empirical and alternative dependency networks. Further robustness checks on the modeling of network weights, the predictability of shocks during COVID-19, and resilience to future hypothetical shocks are performed in Supplementary Notes 4, 5, and 6, respectively.

3.1 Robustness against choice of time period

The baseline dependency network was generated using data collected during the period of September to December, 2019. To assess whether the dependency networks are dynamic across time, we compared the in- and out-weights of each POI across different data collection periods. Figures 18 and 19 shows that the in- and out-weights across different time periods (2019 May – August and 2019 January – April) are highly correlated ($\rho > 0.7$) with the baseline time period (2019 September – December). Moreover, the category pairwise weight proportions were compared across different data collection periods, as shown in Figures 20 and 21. The category pairs that are highly dependent on eachother are consistent across different time periods (2019 May – August and 2019 January – April) with the baseline time period (2019 September – December).

3.2 Computing quartiles of w_{ij} via bootstrap method

As shown in Figure 1 of the main manuscript and other figures in the Supplementary Material (e.g., Figures 10 - 14), edge weights w_{ij} are distributed across 4 orders of magnitude, and there exist edges with very small weights (i.e., $w_{ij} < 0.001$). To test the robustness of the results presented in this paper to such extremely small edge weights, we estimate the confidence interval of each edge weight w_{ij} using bootstrap resampling.

To obtain bootstrapped samples of the dependency weights w_{ij}, the co-visits were sampled randomly with replacement from the original co-visit dataset. For each bootstrap sample the bootstrap replication of the dependency weights w_{ij}^b where $b = 1, 2, 3, ..., 30$ were computed. The 25th percentile of the distribution of the weights, denoted by w_{ij}^{p25} were computed from the sample distribution, and only edges with $w_{ij}^{p25} > 0$ were used in the 'bootstrap network'.

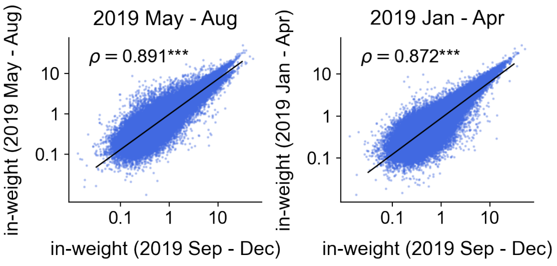

(a) New York, in-weight

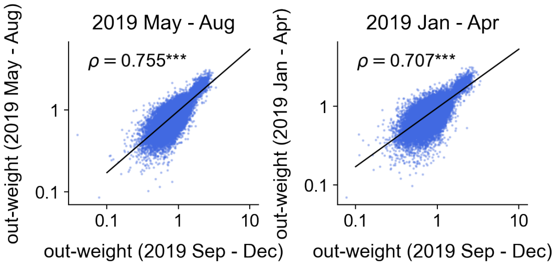

(b) New York, out-weight

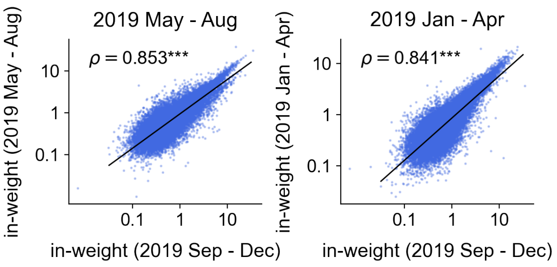

(c) Boston, in-weight

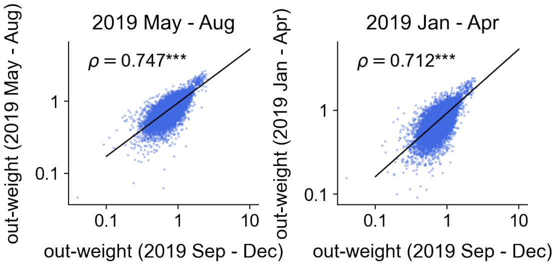

(d) Boston, out-weight

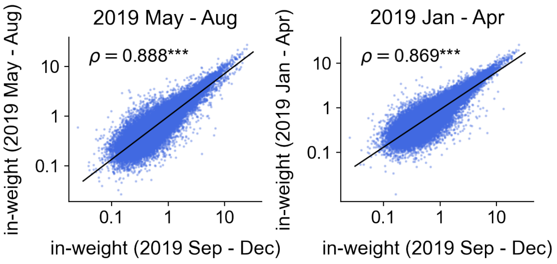

(e) Seattle, in-weight

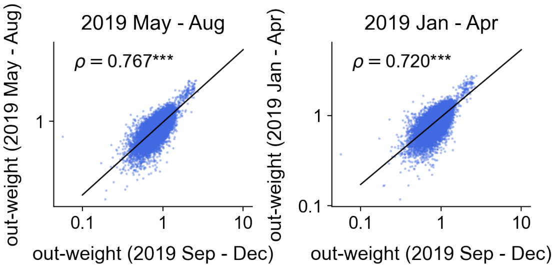

(f) Seattle, out-weight

Supplementary Figure 18: Comparison of in- and out-weights of each POI across different data collection periods for New York, Boston, and Seattle. In- and out-weights across different time periods (2019 May – August and 2019 January – April) are highly correlated ($\rho > 0.7$) with the baseline time period (2019 September – December).

Figure 22 shows the in- and out- weights of POIs before and after applying the bootstrap method to select statistically significant edges. The correlation is generally moderate ($\rho = 0.6$) for out-weight and high ($\rho = 0.9$) for in-weights. The variance becomes larger especially for the nodes with out-weights below 1. Figure 23 shows the out- and in-weights of categories when applying bootstrap method to remove insignificant weights. The correlation is extremely high ($\rho > 0.99$) for both out- and in-weights when aggregated to POI categories, indicating that bootstrapping does not have a significant effect when the analysis is aggregated to the category level.

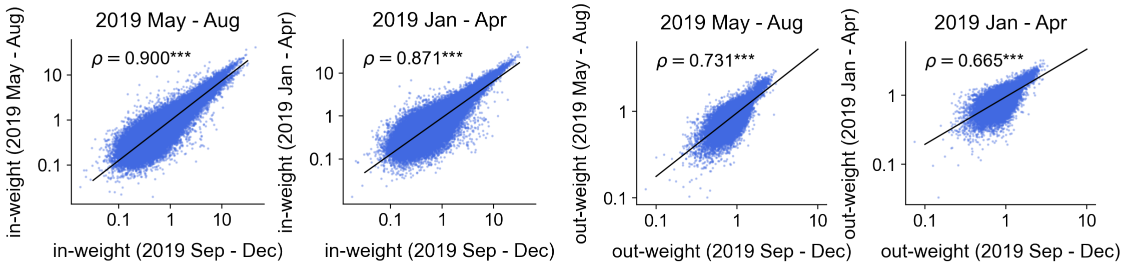

(a) Los Angeles, in-weight **(b)** Los Angeles, out-weight

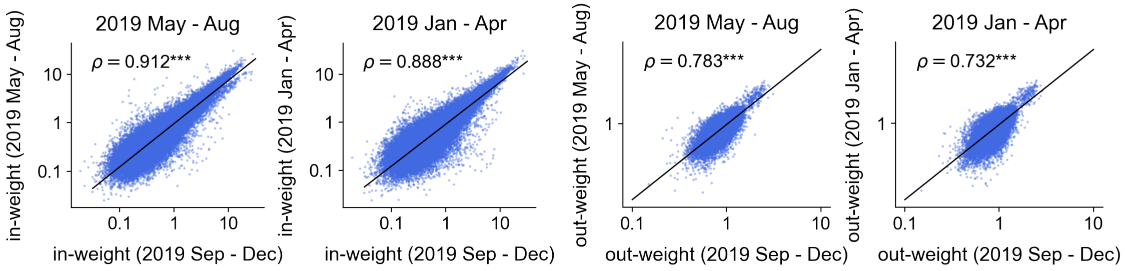

(c) Dallas, in-weight **(d)** Dallas, out-weight

Supplementary Figure 19: Comparison of in- and out-weights of each POI across different data collection periods for Los Angeles and Dallas. In- and out-weights across different time periods (2019 May – August and 2019 January – April) are highly correlated ($\rho > 0.7$) with the baseline time period (2019 September – December).

3.3 Robustness against post-stratification processing

In Supplementary Note 1, analyses showed significant biases in sampling rates of mobile phone users across census block groups and income ranges. To correct for such bias, we conducted post stratification method (Supplementary Note 1.4) and used the corrected data to estimate and analyze the dependency networks. Here, we measure how different the estimates of out- and in-weights of POIs would be if we did not apply the post-stratification technique to correct for biases in mobile phone data. In Figure 24, we observe that the correlation is high ($\rho > 0.8$) for both out- and in-weights even though there was significant bias in sample rates across CBGs and income groups.

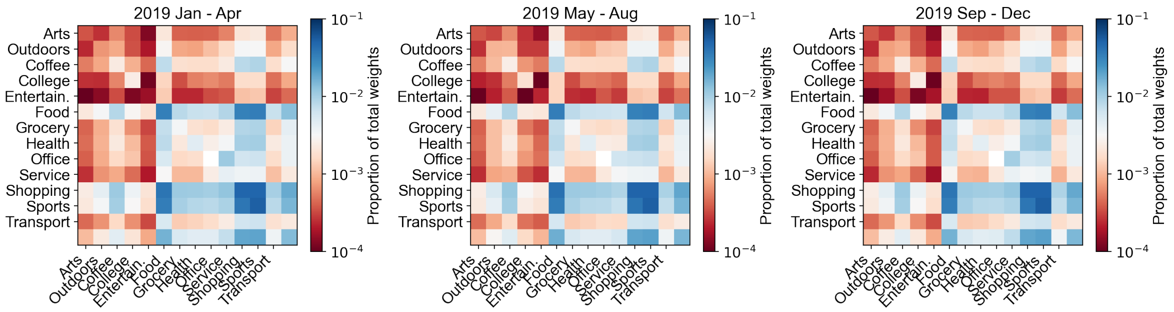

(a) New York

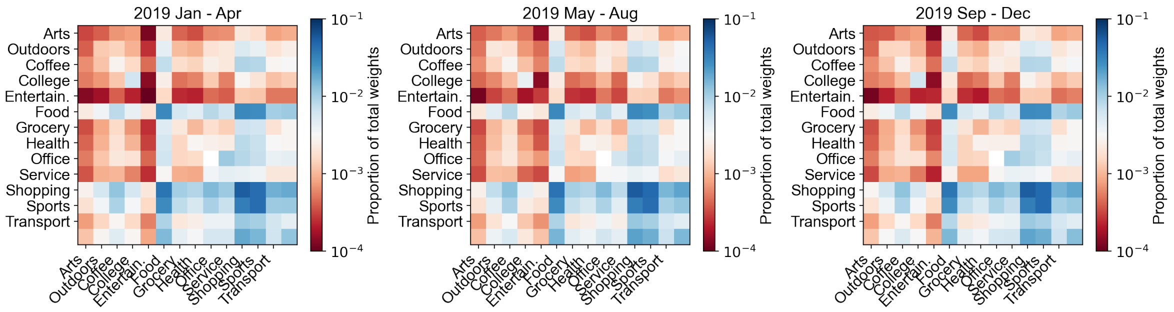

(b) Boston

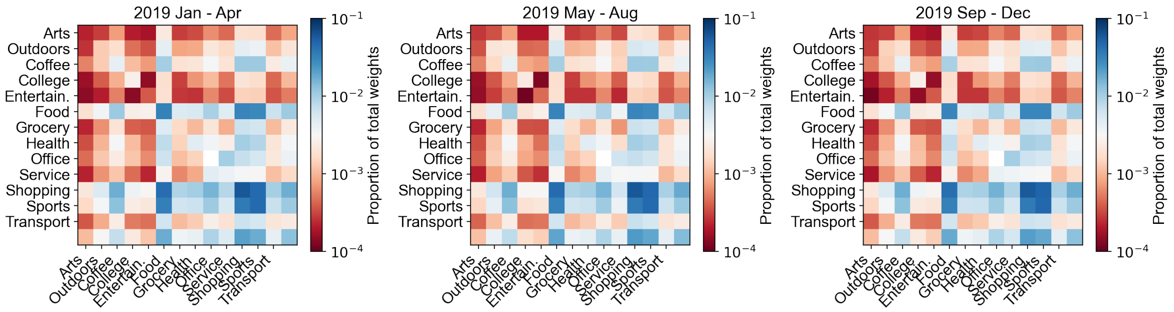

(c) Seattle

Supplementary Figure 20: Comparison of category pairwise weight proportion across different data collection periods, for New York, Boston, and Seattle. The category pairs that are highly dependent on eachother are consistent across different time periods (2019 May – August and 2019 January – April) with the baseline time period (2019 September – December).

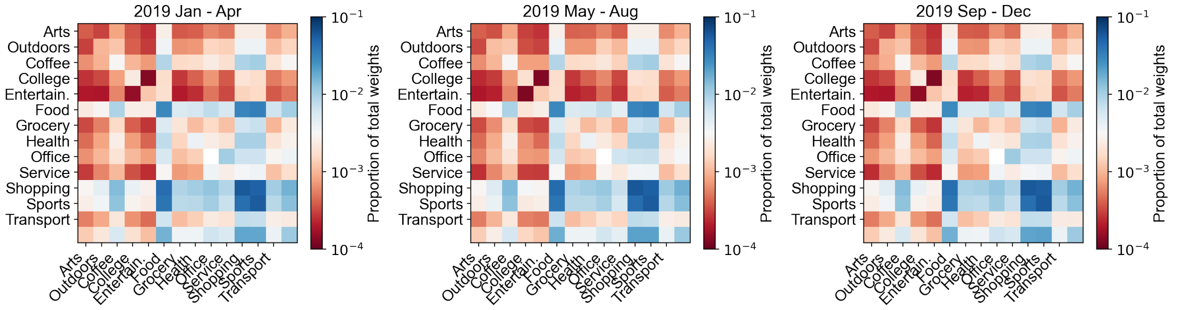

(a) Boston

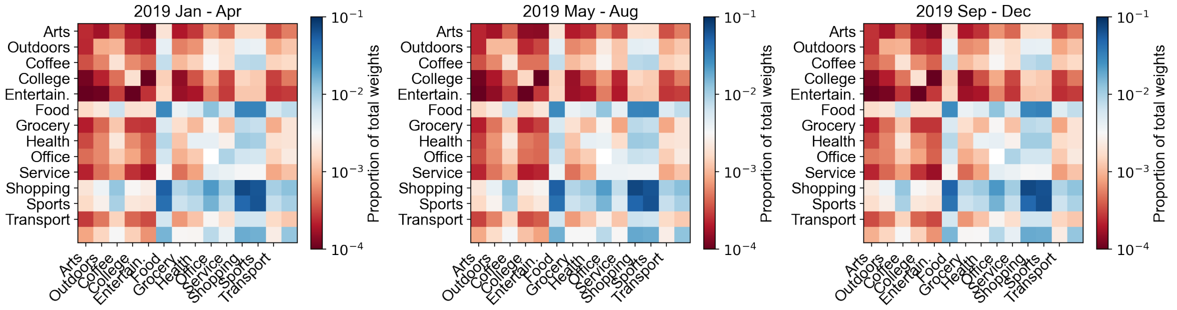

(b) Boston

Supplementary Figure 21: Comparison of category pairwise weight proportion across different data collection periods, for Los Angeles and Dallas. The category pairs that are highly dependent on eachother are consistent across different time periods (2019 May – August and 2019 January – April) with the baseline time period (2019 September – December).

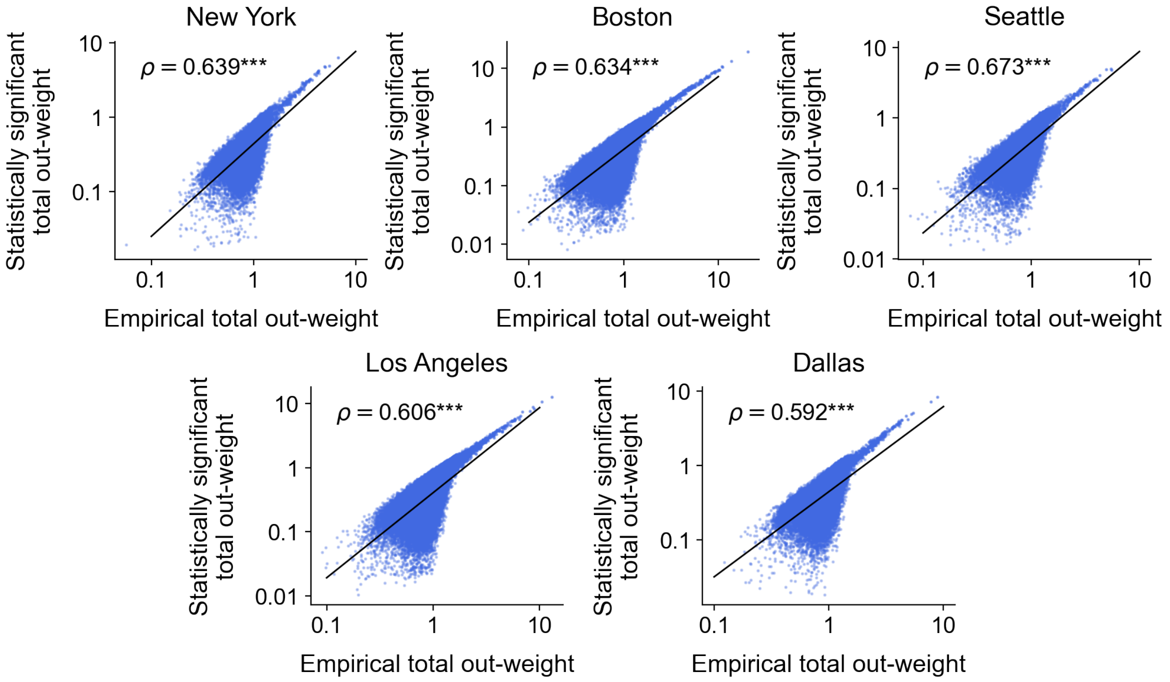

(a) Out-weights of POIs

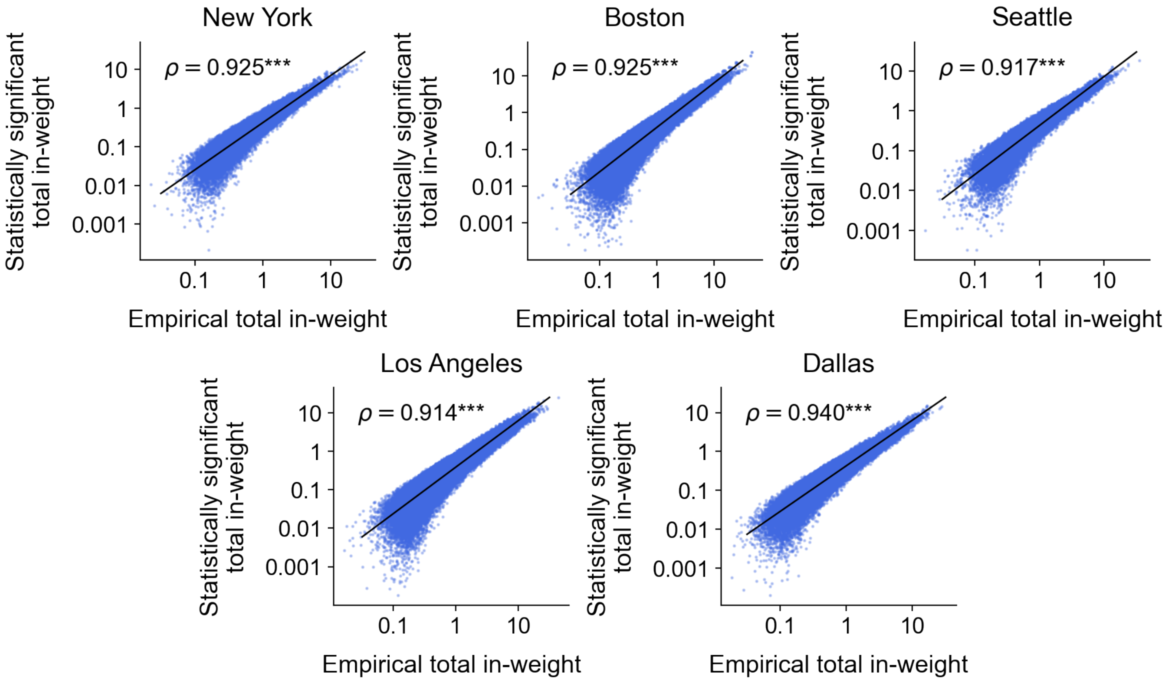

(b) In-weights of POIs

Supplementary Figure 22: Out- and in-weights of POIs when applying bootstrap method to remove smaller weights. The correlation is generally moderate ($\rho = 0.6$) for out-weight and high ($\rho = 0.9$) for in-weights.

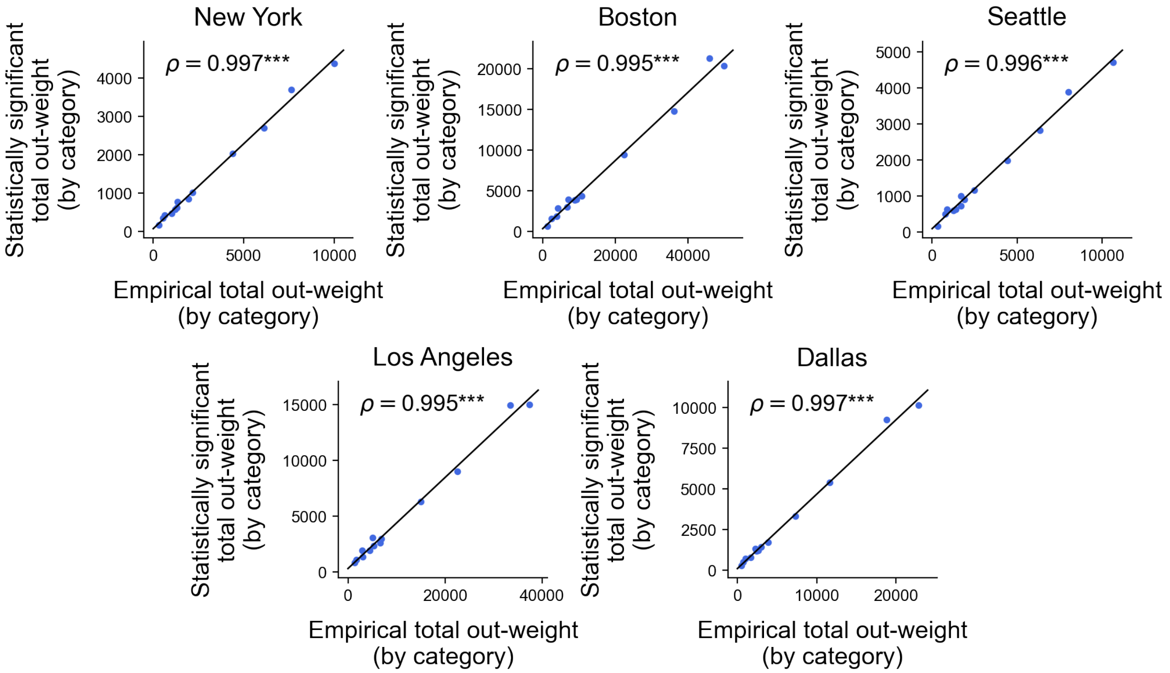

(a) Out-weights of categories

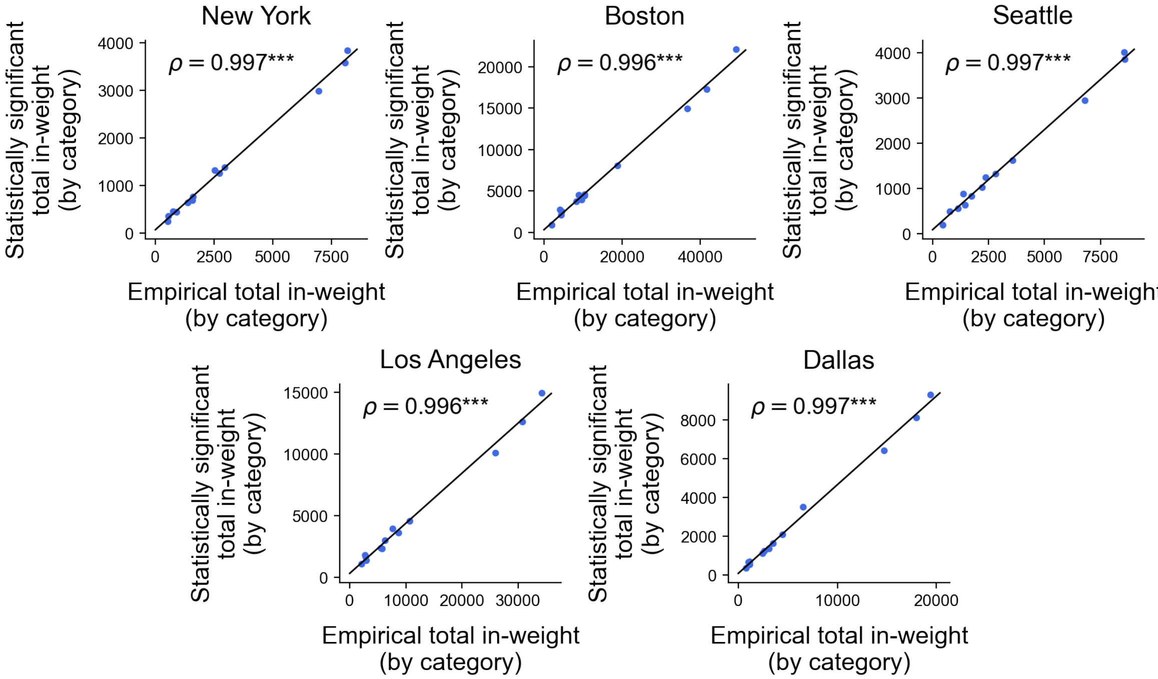

(b) In-weights of categories

Supplementary Figure 23: Out- and in-weights of categories when applying bootstrap method to remove insignificant weights. The correlation is extremely high ($\rho > 0.99$) for both out- and in-weights when aggregated to POI categories.

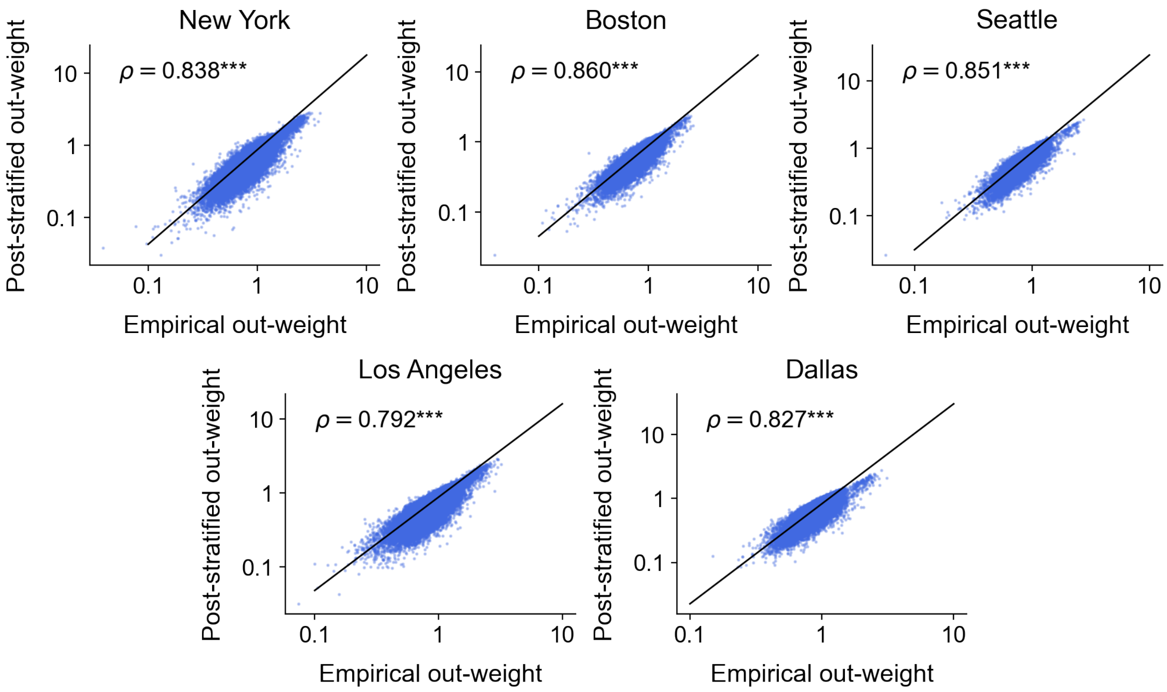

(a) Out-weights of POIs

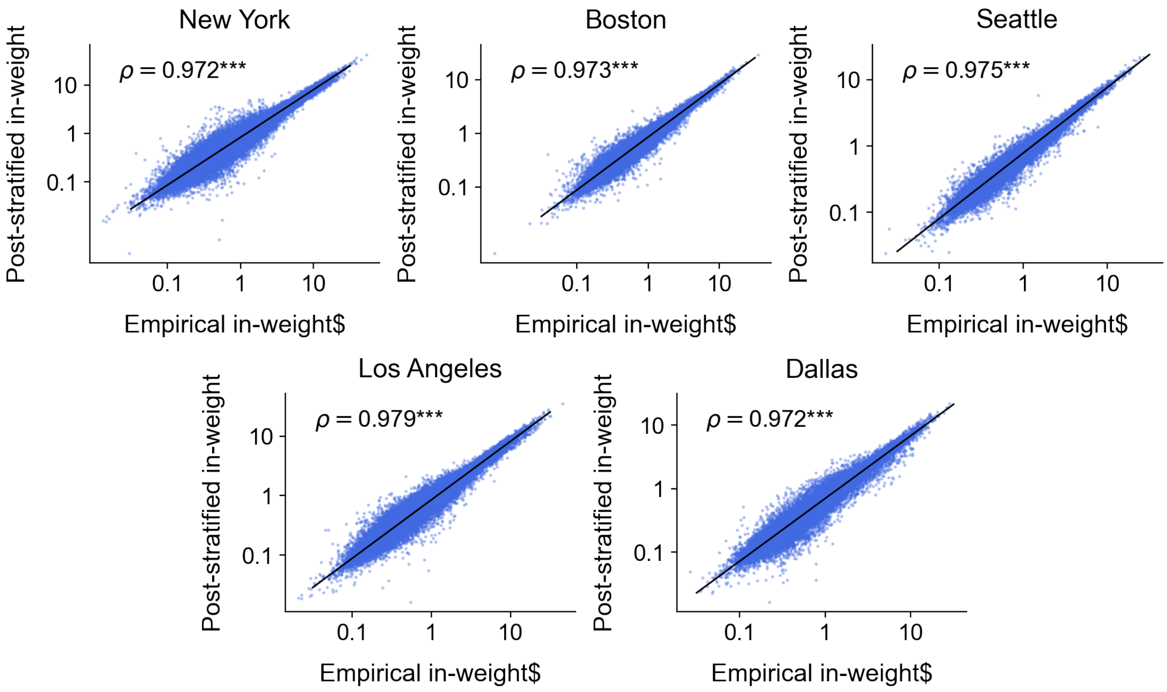

(b) In-weights of POIs

Supplementary Figure 24: Out- and in-weights of POIs when applying post-stratification to correct for biases in mobile phone data. The correlation is high ($\rho > 0.8$) for both out- and in-weights even though there was significant bias in sample rates across CBGs and income groups.

4 Modeling dependency network weights

4.1 Gravity-based null networks

To further understand the structural properties of the dependency networks, we generated null networks that satisfy the following properties:

- the weight w_{ij} decays with physical distance
- the in-weight w_{ij} is larger for nodes with larger visitation n_i

To generate such null networks that preserve such qualities, we utilized the generalized gravity law as the theoretical starting point, where we can model the number of common visitors as the gravity component g_{ij}, which is the product of the total number of visitors to places i and j, n_i and n_j, divided by a function of the distance between i and j, d_{ij}, which takes the form $f(d_{ij}) = (d_0 + d_{ij})^\gamma$:

$$g_{ij} = n_i n_j / (d_0 + d_{ij})^\gamma \tag{2}$$

where n_i and n_j are the total number of visits to POIs i and j, d_{ij} is the physical distance between POIs i and j, d_0 is the distance cutoff parameter, and γ is the exponent parameter of the gravity model. Parameters d_0 and γ were fitted empirically to maximize the correlation between g_{ij} and n_{ij}, which is the total number of common visitors between POIs i and j, and as shown in Figure 25, $d_0 = 0.2$ and $\gamma = 1.5$ showed the highest correlation when the values were logged and not logged.

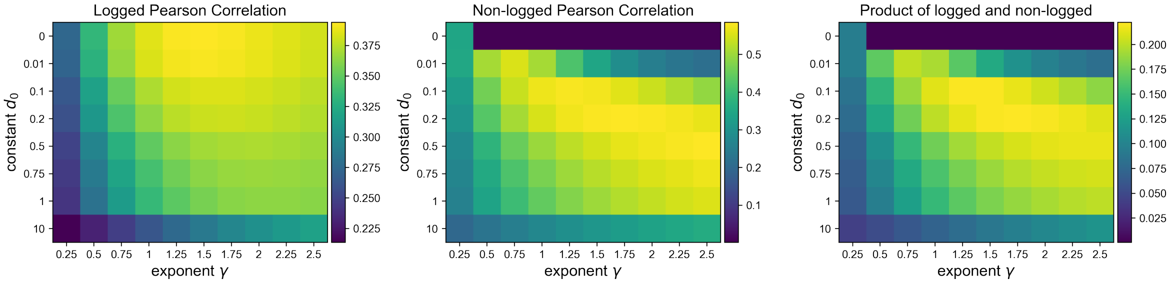

Supplementary Figure 25: Correlation between the observed n_{ij} values and the gravity component g_{ij} under different model parameters

Using the fitted gravity model $w_{ij} = n_{ij}/n_i \sim n_j/(d_0 + d_{ij})^\gamma$, we now construct null networks that mimic the physical properties of the actual dependency networks. To enable a fair comparison between the actual dependency network and the null network, we constructed the null networks while fulfilling the following two properties:

1. the in-degree of each node are kept consistent in the null network
2. the in-weight of each node are kept consistent in the null network

To achieve this, first we construct a linear relationship between the gravity component $g_{ij} = n_j/(d_0 + d_{ij})^\gamma$ and w_{ij}, $g_{ij} = \phi(w_{ij})$. Then, for each edge in the actual dependency network connecting i and j with a dependency weight w_{ij}, we compute its theoretical gravity component using the linear relationship ϕ and select an alternative node with the same level of corresponding gravity component from its 10,000 closest nodes, and is assigned the same weight w_{ij}. This algorithm enables us to

construct a null network where we 1) maintain the linear relationship between w_{ij} and g_{ij}, 2) the same number of in-edges are selected for each node, and 3) the total in-weight for each node is kept consistent.

Figures 26 to 28 show the map visualizations of the actual (left) and null (right) networks for the five cities. In all cities, we can observe the null network being more locally clustered around large POIs, while the actual network is more dispersed and contains more long-distance connections. Note that the in-degrees and in-weights of all nodes are equivalent across the two networks. Figure 29 shows the histogram of physical distances of all edges in the actual and null networks. Figure 30 compares the proportion of edge weights among POI category pairs across the actual and null networks. The proportion of edge weights was computed by taking the sum of weights that connect the vertical and horizontal categories and dividing that by the total weights that exist in the network. The patterns of dependency are consistent across cities, with Service and Transport node depending more on other nodes in the real network compared to the null network.

4.2 Regression model of w_{ij}

Results comparing the relationship between dependency weights and physical distance suggest that the dependency weights could be dictated by physical factors. Here, we model dependency weights w_{ij} with various physical factors to investigate how much of the variance we observe in dependency weights can be explained by such factors. We build simple linear regression models of the form:

$$\log_{10} w_{ij} \sim \log_{10} d_{ij} + n_j + \eta_i + \eta_j + \theta_i + \theta_j \tag{3}$$

where $\log_{10} w_{ij}$ denotes the logged dependency weight of POI i on POI j, and:

- $\log_{10} d_{ij}$ is the logged Haversine distance between POIs i and j.
- n_j is the number of visitors that POI j receives. The premise is that popular POIs are depended more by other POIs. This variable was standardized ($(x - \mu)/\sigma$) prior to the analysis for comparison across cities.
- η_i is the fixed effect (FE) for POI i's subcategory. There are 97 subcategories in the POI dataset, and examples are shown in Figure 6.
- θ_i is the fixed effect for POI i's located Public Use Microdata Area (PUMA).

The regression results are shown in Table 3. To save computation time, 1 million edges were randomly sampled to include in the regression. The resulting R^2 or the coefficients were robust against different sampling runs. Overall, the adjusted R^2 was relatively low (between 0.10 and 0.16 for all cities), suggesting that physical factors determine a small $\%$ of the variance of dependency weights. The coefficients are similar across cities, with the logged Haversine distance showing a slower decay compared to the decay observed in Figure 10, which is due to the samples in the regression being limited to ones that have non-zero weights.

4.3 Robustness against different network instances

Table 4 shows the same regression results using the selected network edges using the bootstrap method introduced in Supplementary Note 3.2. The bootstrap method removed edges with weights that were almost zero (if the 25th percentile of the bootstrap sample distribution was below zero), and as a

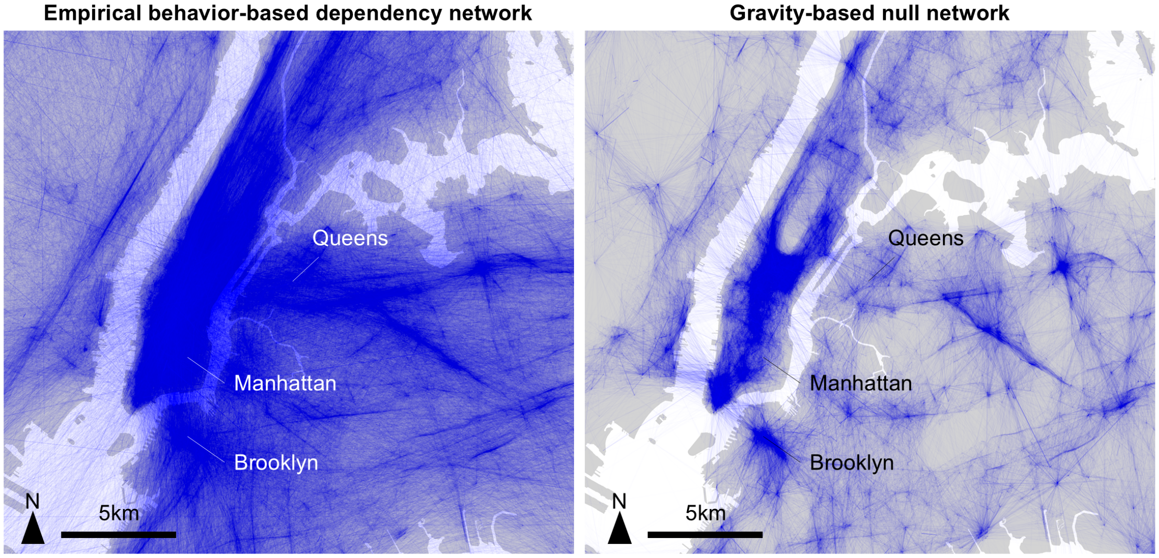

(a) New York

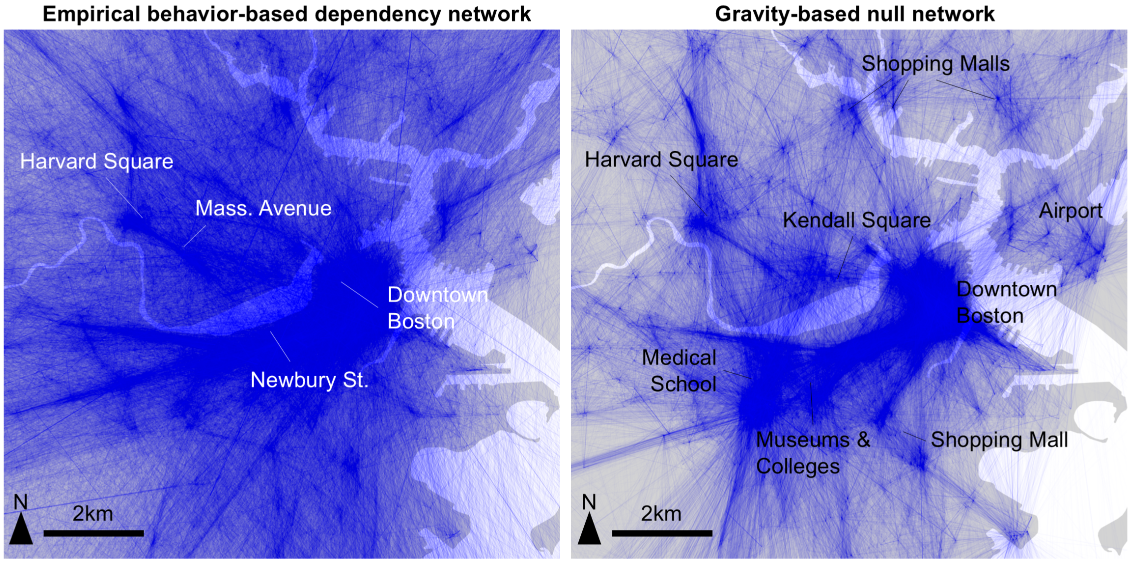

(b) Boston

Supplementary Figure 26: Comparison of actual and null networks in New York and Boston. For visual purposes, around 1 million links are shown for both the actual and null networks. Maps were produced in Python using the TIGER shapefiles from the U.S. Census Bureau [15].

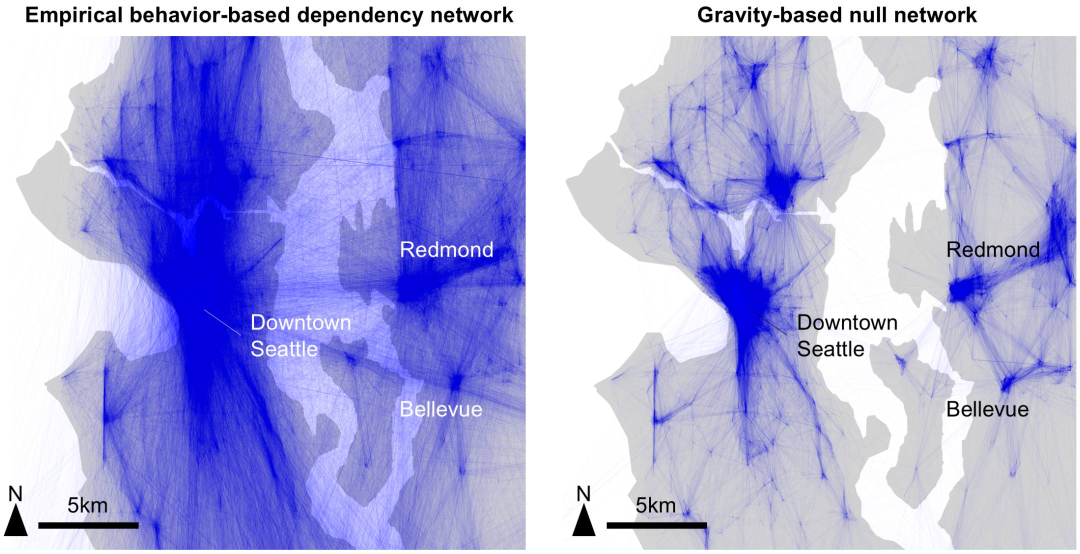

(a) New York

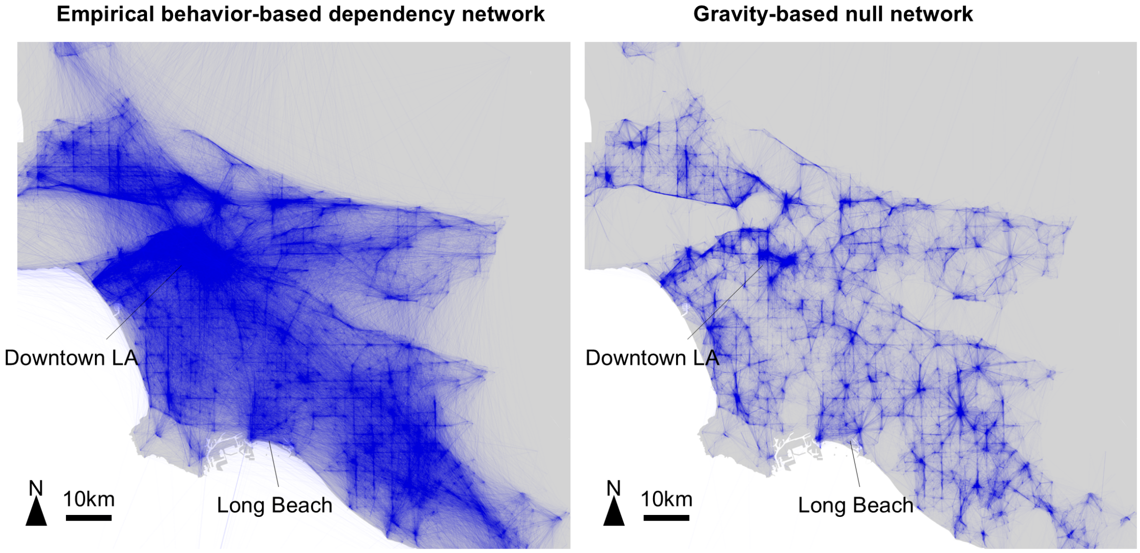

(b) Boston

Supplementary Figure 27: Comparison of actual and null networks in Seattle and Los Angeles. For visual purposes, around 1 million links are shown for both the actual and null networks. Maps were produced in Python using the TIGER shapefiles from the U.S. Census Bureau [15].

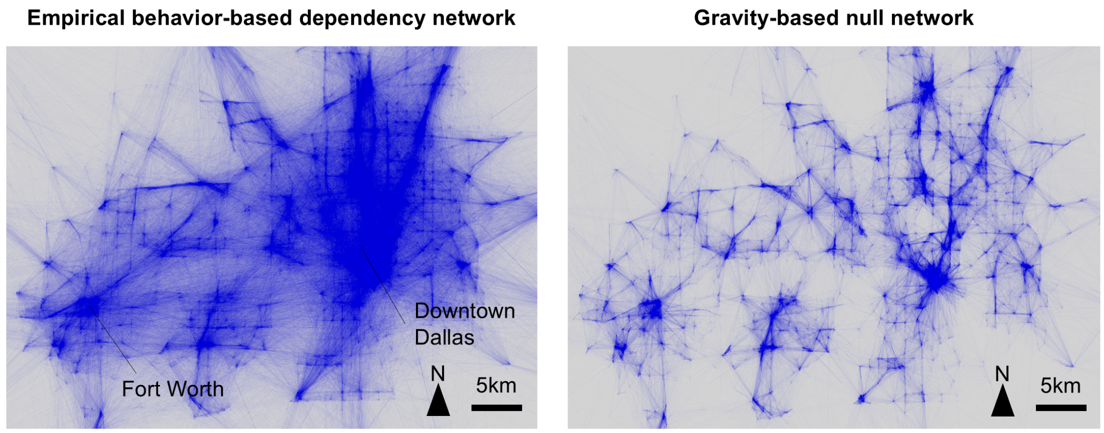

(a) Dallas

Supplementary Figure 28: Comparison of actual and null networks in Dallas. For visual purposes, around 1 million links are shown for both the actual and null networks. Maps were produced in Python using the TIGER shapefiles from the U.S. Census Bureau [15].

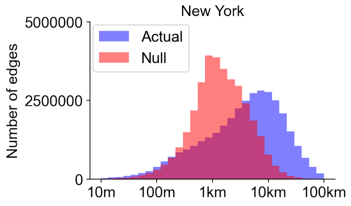

(a) New York

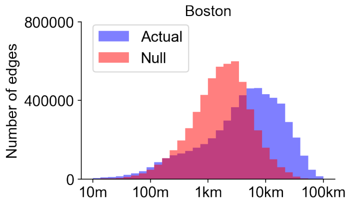

(b) Boston

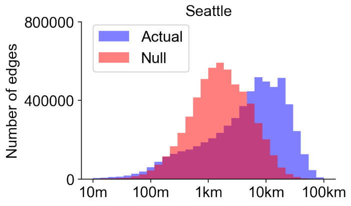

(c) Seattle

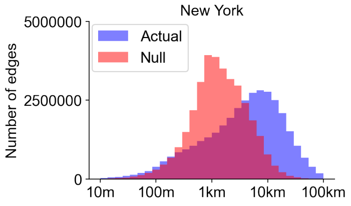

(d) Los Angeles

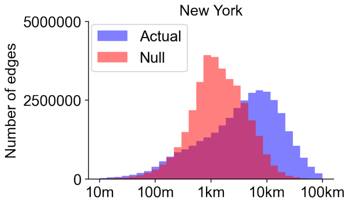

(e) Dallas

Supplementary Figure 29: Comparison between real and null network on the proportion of edge weights among POI category pairs. The proportion of edge weights was computed by taking the sum of weights that connect the vertical and horizontal categories and dividing that by the total weights that exist in the network. The patterns of dependency are consistent across cities, with Service and Transport node depending more on other nodes in the real network compared to the null network.

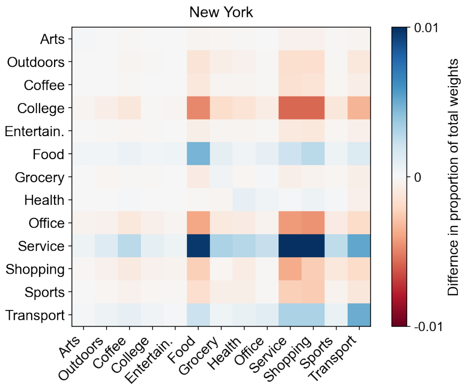

(a) New York

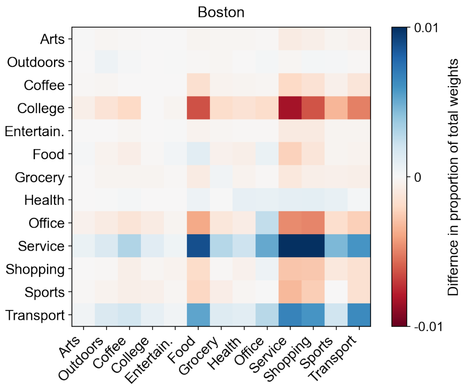

(b) Boston

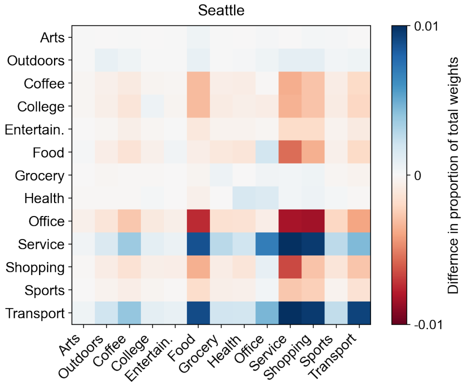

(c) Seattle

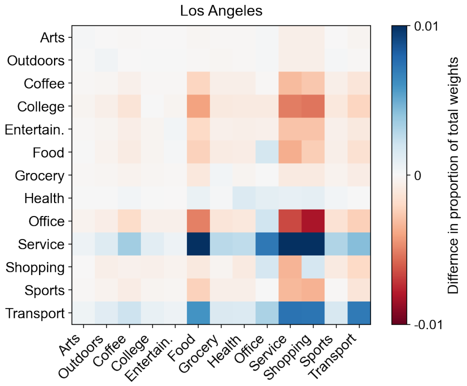

(d) Los Angeles

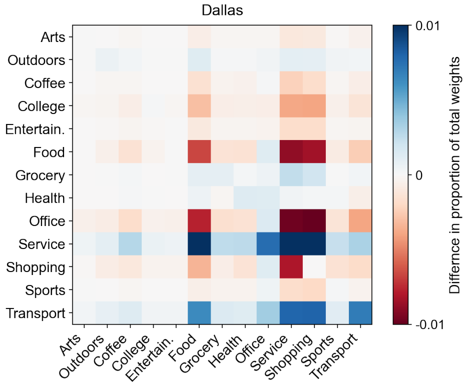

(e) Dallas

Supplementary Figure 30: Histograms of edge distances between actual and null networks. The actual networks contain more long distance edges than the null networks.

Supplementary Table 3: Linear regression models predicting the logged dependency weights between POIs i and j ($log_{10} w_{ij}$) in US cities using the logged Haversine distance ($\log_{10} d_{ij}$), the popularity in visits to place j (v_j), and the subcategory fixed effects and PUMA fixed effects of POIs i and j. Variables were centered and standardized prior to analysis. Regression coefficients are reported, followed by coefficient estimate standard error in parentheses, estimate P value and a 95% confidence interval. Similar analyses are presented in Tables 4 - 7.

	Dependent Variable: $\log_{10} w_{ij}$				
Variable	New York	Boston	Seattle	Los Angeles	Dallas
Constant	-2.224 (0.038)	-2.265 (0.017)	-2.281 (0.032)	-2.295 (0.024)	-2.355 (0.037)
	P <0.001	P <0.001	P <0.001	P <0.001	P <0.001
	(-2.299, -2.149)	(-2.298, -2.231)	(-2.344, -2.218)	(-2.344, -2.247)	(-2.428, -2.283)
$\log_{10} d_{ij}$	-0.129 (0.0005)	-0.147 (0.0005)	-0.134 (0.0005)	-0.127 (0.0005)	-0.143 (0.0005)
	P <0.001	P <0.001	P <0.001	P <0.001	P <0.001
	(-0.130, -0.128)	(-0.148, -0.146)	(-0.135, -0.133)	(-0.128, -0.126)	(-0.144, -0.142)
v_j (standardized)	0.022 (0.0004)	0.026 (0.0004)	0.027 (0.0004)	0.021 (0.0004)	0.022 (0.0003)
	P <0.001	P <0.001	P <0.001	P <0.001	P <0.001
	(0.0209, 0.0225)	(0.027, 0.028)	(0.027, 0.028)	(0.020, 0.021)	(0.022, 0.023)
Subcategory FE for i and j	Yes	Yes	Yes	Yes	Yes
PUMA FE for i and j	Yes	Yes	Yes	Yes	Yes
Observations (sampled)	1,000,000	1,000,000	1,000,000	1,000,000	1,000,000
R^2	0.090	0.118	0.122	0.110	0.116
Adj. R^2	0.089	0.117	0.121	0.110	0.115

result, as shown in Figure 22(a), the POIs with smaller weights (especially out-weights) experienced a significant decrease in edge weights. The regression results show a much higher R^2 compared to the original results using all of the edges, explaining around 20% to 30% of the variance in the dependency weights. The results are once again consistent across cities, with regression coefficients in the same range.

Table 5 shows the same regression results using the dependency network computed using only short stays (< 4 hours). The results of the regression (adjusted R squared and the model coefficients) are consistent with the results from the full network shown in Table 3.

4.4 Robustness against co-visit detection parameters

The regression analysis showed two main findings: 1) the physical factors can only explain a fraction of the variance in dependency weights, and 2) the coefficient of the logged Haversine distance is moderate compared to distance exponents used in traditional models such as the Gravity Model (i.e., -2). To assess the robustness of these findings of the regression model, the regression was performed using dependency networks generated using different co-visit detection parameters (also used in Supplementary Note 2.3). The 10 pairs of parameters ($T_s = [1, 2, 3, 5, unlimited]$ and $T_c = [1, 3, 6, 12, 24]$ hours) were used to generate the dependency networks for each city, and the regression results (adjusted R^2 and coefficient for Haversine distance) are shown in Figure 31. The matrix plots across the five metropolitan areas show similar patterns, both the adjusted R^2 and coefficient for Haversine distance are relatively different when the maximum time difference is 1 hour. In the rest of the parameter pairs, we obtain a similar result compared to Table 3.

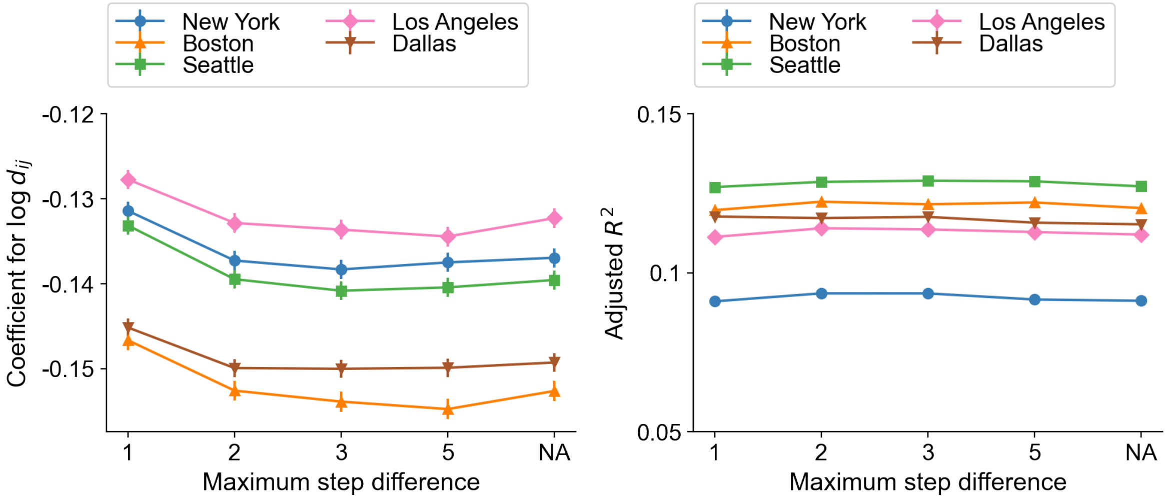

(a) Step difference parameter

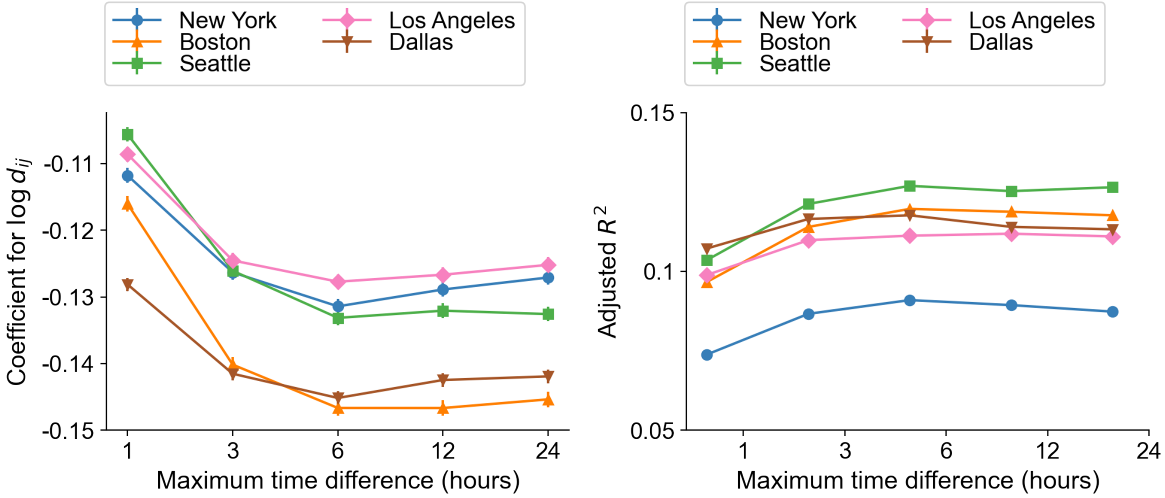

(b) Time difference parameter

Supplementary Figure 31: Regression results for dependency weights when using different co-visit detection parameters, continued for Los Angeles and Dallas. The error bars show the 95% confidence intervals for the coefficients. Except for the networks generated using maximum time difference of 1 hour, the results (adjusted R^2 and coefficient for Haversine distance) are relatively similar as the results in Table 3.

Supplementary Table 4: Linear regression models predicting the logged dependency weights between POIs i and j ($log_{10} w_{ij}$) in US cities which were selected using the bootstrap method, using the logged Haversine distance ($\log_{10} d_{ij}$), the popularity in visits to place j (v_j), and the subcategory fixed effects and PUMA fixed effects of POIs i and j. Variables were centered and standardized prior to analysis.

	Dependent Variable: $\log_{10} w_{ij}$				
Variable	New York	Boston	Seattle	Los Angeles	Dallas
Constant	-2.117 (0.051)	-1.978 (0.021)	-2.057 (0.053)	-2.250 (0.030)	-2.048 (0.056)
	P <0.001	P <0.001	P <0.001	P <0.001	P <0.001
	(-2.218, -2.016)	(-2.020, -1.935)	(-2.162, -1.951)	(-2.310, -2.190)	(-2.160, -1.937)
$\log_{10} d_{ij}$	-0.162 (0.0005)	-0.199 (0.0005)	-0.186 (0.0005)	-0.188 (0.0005)	-0.232 (0.0005)
	P <0.001	P <0.001	P <0.001	P <0.001	P <0.001
	(-0.163, -0.161)	(-0.200, -0.198)	(-0.187, -0.184)	(-0.189, -0.187)	(-0.233, -0.231)
v_j (standardized)	0.026 (0.0004)	0.033 (0.0004)	0.032 (0.0004)	0.030 (0.0004)	0.032 (0.0004)
	P <0.001	P <0.001	P <0.001	P <0.001	P <0.001
	(0.025, 0.027)	(0.032, 0.034)	(0.031, 0.033)	(0.030, 0.032)	(0.031, 0.033)
Subcategory FE for i and j	Yes	Yes	Yes	Yes	Yes
PUMA FE for i and j	Yes	Yes	Yes	Yes	Yes
Observations (sampled)	1,000,000	1,000,000	1,000,000	1,000,000	1,000,000
R^2	0.136	0.193	0.172	0.215	0.245
Adj. R^2	0.135	0.193	0.172	0.215	0.244

4.5 Robustness against different time periods

To check the robustness of the results with respect to the time periods used to generate the dependency network, the same regression experiments were performed using 2019 January – April data and 2019 May – August data, as shown in Tables 6 and 7. We can observe that the R^2 values and the regression coefficients are similar with those obtained in Table 3.

Supplementary Table 5: Linear regression models predicting the logged dependency weights between POIs i and j ($log_{10} w_{ij}$) in US cities which were selected using only short stays (¡ 4 hrs), using the logged Haversine distance ($\log_{10} d_{ij}$), the popularity in visits to place j (v_j), and the subcategory fixed effects and PUMA fixed effects of POIs i and j. Variables were centered and standardized prior to analysis.

	Dependent Variable: $\log_{10} w_{ij}$				
Variable	New York	Boston	Seattle	Los Angeles	Dallas
Constant	-2.208 (0.039)	-2.220 (0.017)	-2.291 (0.031)	-2.260 (0.025)	-2.276 (0.036)
	P <0.001	P <0.001	P <0.001	P <0.001	P <0.001
	(-2.285, -2.132)	(-2.229, -2.160)	(-2.354, -2.228)	(-2.310, -2.209)	(-2.348, -2.205)
$\log_{10} d_{ij}$	-0.130 (0.0005)	-0.149 (0.0005)	-0.134 (0.0005)	-0.125 (0.0005)	-0.144 (0.0005)
	P <0.001	P <0.001	P <0.001	P <0.001	P <0.001
	(-0.131, -0.129)	(-0.150, -0.148)	(-0.135, -0.133)	(-0.126, -0.124)	(-0.145, -0.143)
v_j (standardized)	0.022 (0.0004)	0.029 (0.0004)	0.028 (0.0004)	0.021 (0.0004)	0.024 (0.0003)
	P <0.001	P <0.001	P <0.001	P <0.001	P <0.001
	(0.021, 0.022)	(0.028, 0.030)	(0.027, 0.029)	(0.021, 0.022)	(0.023, 0.025)
Subcategory FE for i and j	Yes	Yes	Yes	Yes	Yes
PUMA FE for i and j	Yes	Yes	Yes	Yes	Yes
Observations (sampled)	1,000,000	1,000,000	1,000,000	1,000,000	1,000,000
R^2	0.097	0.126	0.130	0.117	0.120
Adj. R^2	0.096	0.125	0.130	0.116	0.120

Supplementary Table 6: Linear regression models predicting the logged dependency weights between POIs i and j ($log_{10} w_{ij}$) in US cities using network from 2019 January - April data.

	Dependent Variable: $\log_{10} w_{ij}$ (2019 January - April data)				
Variable	New York	Boston	Seattle	Los Angeles	Dallas
Constant	-2.219 (0.038)	-2.241 (0.017)	-2.294 (0.031)	-2.247 (0.024)	-2.280 (0.038)
	P <0.001	P <0.001	P <0.001	P <0.001	P <0.001
	(-2.295, -2.143)	(-2.275, -2.207)	(-2.356, -2.232)	(-2.295, -2.199)	(-2.356, -2.203)
$\log_{10} d_{ij}$	-0.129 (0.0005)	-0.146 (0.0005)	-0.132 (0.0005)	-0.128 (0.0005)	-0.144 (0.0005)
	P <0.001	P <0.001	P <0.001	P <0.001	P <0.001
	(-0.130, -0.127)	(-0.148, -0.145)	(-0.133, -0.131)	(-0.129, -0.126)	(-0.145, -0.143)
v_j (standardized)	0.019 (0.0004)	0.029 (0.0004)	0.026 (0.0004)	0.019 (0.0004)	0.021 (0.0004)
	P <0.001	P <0.001	P <0.001	P <0.001	P <0.001
	(0.018, 0.020)	(0.028, 0.029)	(0.025, 0.027)	(0.018, 0.020)	(0.019, 0.021)
Subcategory FE for i and j	Yes	Yes	Yes	Yes	Yes
PUMA FE for i and j	Yes	Yes	Yes	Yes	Yes
Observations (sampled)	1,000,000	1,000,000	1,000,000	1,000,000	1,000,000
R^2	0.093	0.121	0.121	0.112	0.118
Adj. R^2	0.092	0.120	0.121	0.111	0.118

Supplementary Table 7: Linear regression models predicting the logged dependency weights between POIs i and j ($log_{10}w_{ij}$) in US cities using network from 2019 May - August data.

	Dependent Variable: $\log_{10} w_{ij}$ (2019 May - August data)				
Variable	New York	Boston	Seattle	Los Angeles	Dallas
Constant	-2.247 (0.038)	-2.287 (0.017)	-2.351 (0.029)	-2.334 (0.023)	-2.346 (0.035)
	P <0.001	P <0.001	P <0.001	P <0.001	P <0.001
	(-2.321, -2.172)	(-2.321, -2.253)	(-2.409, -2.292)	(-2.387, -2.294)	(-2.415, -2.276)
$\log_{10} d_{ij}$	-0.131 (0.0005)	-0.148 (0.0005)	-0.134 (0.0005)	-0.128 (0.0005)	-0.142 (0.0005)
	P <0.001	P <0.001	P <0.001	P <0.001	P <0.001
	(-0.131, -0.129)	(-0.149, -0.146)	(-0.135, -0.133)	(-0.129, -0.127)	(-0.143, -0.141)
v_j (standardized)	0.020 (0.0004)	0.023 (0.0004)	0.024 (0.0004)	0.019 (0.0004)	0.021 (0.0003)
	P <0.001	P <0.001	P <0.001	P <0.001	P <0.001
	(0.019, 0.021)	(0.022, 0.024)	(0.024, 0.026)	(0.017, 0.019)	(0.020, 0.021)
Subcategory FE for i and j	Yes	Yes	Yes	Yes	Yes
PUMA FE for i and j	Yes	Yes	Yes	Yes	Yes
Observations (sampled)	1,000,000	1,000,000	1,000,000	1,000,000	1,000,000
R^2	0.091	0.114	0.119	0.109	0.114
Adj. R^2	0.090	0.114	0.119	0.108	0.114

5 Impacts of behavior-based dependency during the COVID-19 pandemic

5.1 Analysis of visitation losses in cities

To investigate the usefulness of the dependency network for understanding the resilience of businesses, we construct a regression modeling framework that predicts the change in visitation patterns to a POI using information about the change in visitation patterns to its alters and the dependency network. The observed change in visits to different places is computed by:

$$\tilde{v}_i = \Big(\frac{v_i^{after}}{v_i^{before}} - 1\Big) * 100(\%) \tag{4}$$

where v_i^{before} and v_i^{after} denote the number of visits to place i before the pandemic (September - December 2019) and during the pandemic period (March - June 2020), respectively. The left side panels in Figure 32 plots the distribution of the change in visits $\tilde{v}_i$ in the five metropolitan areas. Overall, as expected, we observe a peak at around $\tilde{v}_i = -75$ for all cities, suggesting the overall substantial decrease in visits during the initial stages of the pandemic. The right side panels in Figure 32 plots the average change in visits across different POI categories. We observe heterogeneity across POI categories, where Arts, Colleges, Food, and Sports POIs experienced the largest losses during the initial stages of the pandemic. On the other hand, Outdoor and Grocery places had a minor impact compared to the other POI types. Moreover, prior studies have found that POIs located in urban areas were affected more compared to rural areas (e.g., [18]). Therefore, we take into consideration the effects of the category of the places and the area in which the POIs are located to predict the visitation losses to POIs.

To test the hypothesis that the change in visitation patterns to a POI can be modeled using information about the change in visitation patterns to its alters and the dependency network, we first investigate the simple correlation between the two metrics. Figure 33 shows the partial correlation $\rho(\tilde{v}_i, \sum_j w_{ij}\tilde{v}_i)$, where POI i and js' categories are A (vertical) and B (horizontal), respectively. Overall, several category pairs exhibit significant and strong correlation. For example, coffee POIs' changes in visits are strongly correlated with the weighted changes in visits to connected food, service, and shopping POIs across the five metropolitan areas. This significant and strong correlation suggests that the changes in the visitation of the ego may be predicted using the information of the alter POIs connected via the dependency network.

5.2 Model specification and estimation results

Using the correlations revealed in the previous section, we build a simple linear regression model of the form:

$$\tilde{v}_i \sim \sum_j w_{ij}\tilde{v}_j + \sum_j \delta_{ij}\tilde{v}_j + \eta_i + \theta_i \tag{5}$$

where $\tilde{v}_i$ denotes the change in visitations to POI i during the initial stages of the pandemic (March 1st - May 21st, 2020), and:

- $\sum_j w_{ij}\tilde{v}_j$ is the sum of the neighbors' (POIs j) change in visitations ($\tilde{v}_j$) weighted by the dependency network weights w_{ij}.

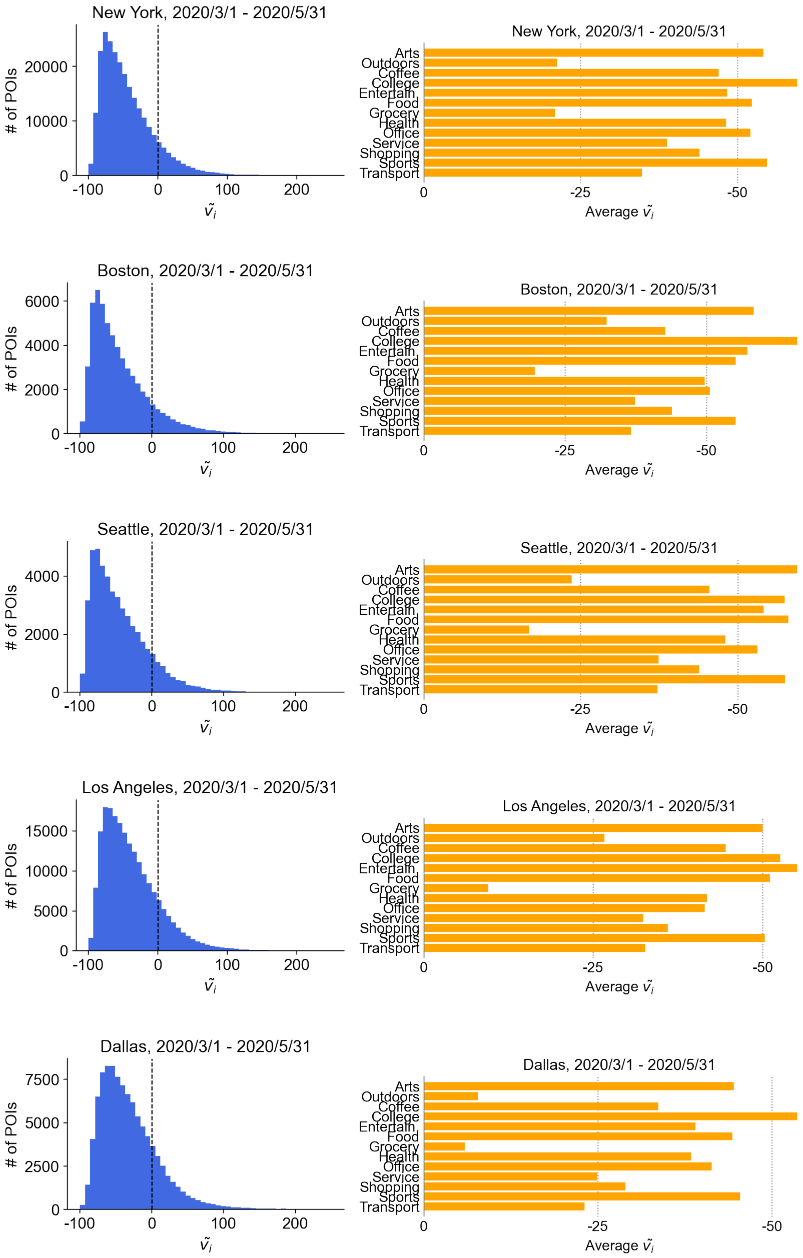

Supplementary Figure 32: Change in visits to POIs in New York, Boston, Seattle, Los Angeles, and Dallas during the pandemic period. The left panels show the distribution of the change in visits, and the right panels show the average effects per POI category.

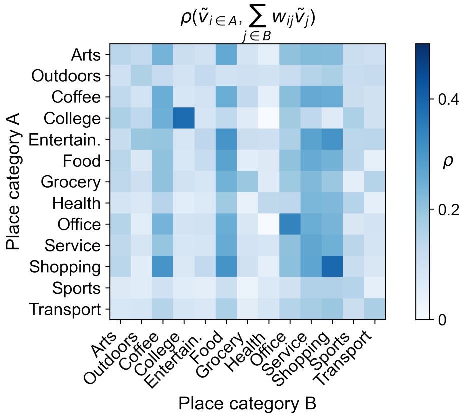

(a) New York

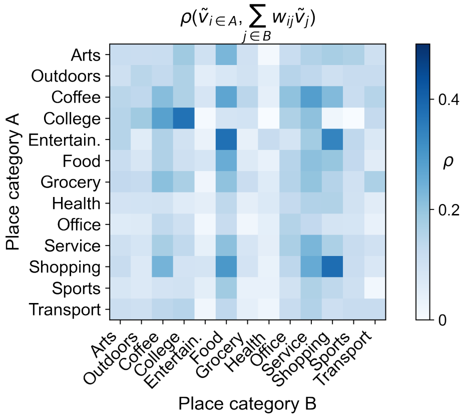

(b) Boston

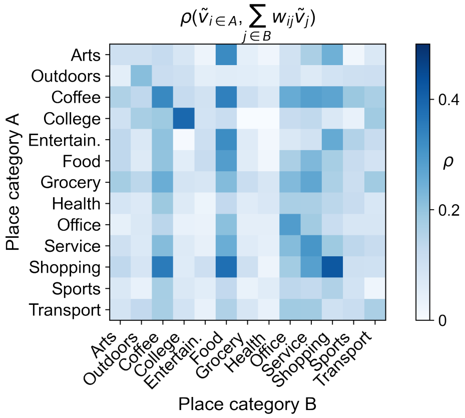

(c) Seattle

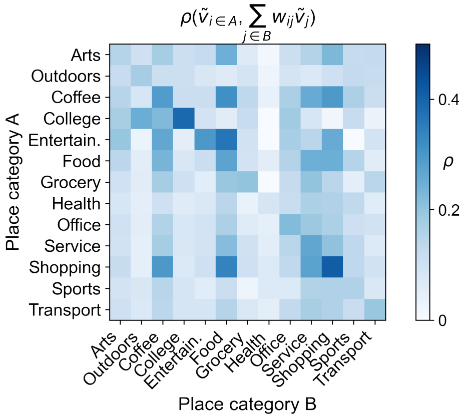

(d) Los Angeles

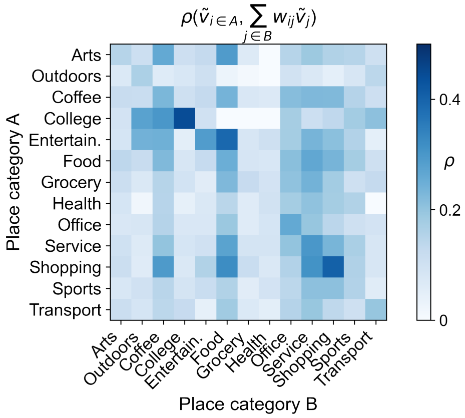

(e) Dallas

Supplementary Figure 33: Correlation between change in visits of ego and the weighted change in visits of alters by category pairs. Significant correlation between several category pairs suggest predictability of $\tilde{v}_i$ using information of alters.

- $\sum_j \delta_{ij}\tilde{v}_j$ is the sum of the neighbors' (POIs j) change in visitations ($\tilde{v}_j$) weighted by the distance based network weights δ_{ij}. The distance based network weights mimic the patterns observed between physical distance and dependency weights, which are constant until 100 meters, but decays as the inverse squared of the actual distance beyond 100 meters.
- η_i is the fixed effect (FE) for POI i's subcategory. There are 97 subcategories in the POI dataset, and examples are shown in Figure 6.
- θ_i is the fixed effect for POI i's located Public Use Microdata Area (PUMA).

Tables 9 to 12 show the regression results for New York, Boston, Seattle, Los Angeles, and Dallas, under the following four models:

(1) using only the subcategory and PUMA fixed effects,

(2) using the distance-based network effects $\sum_j \delta_{ij}\tilde{v}_j$ in addition to the subcategory and PUMA fixed effects,

(3) using the behavior-based dependency network effects $\sum_j w_{ij}\tilde{v}_j$ in addition to the subcategory and PUMA fixed effects, and

(4) using all of the factors introduced in the full model.

Both the distance-based network effects and behavior-based dependency network effects were standardized (by subtracting the mean and dividing by the standard deviation) before analysis, therefore the magnitude of the coefficients can be compared.

In all cities, the model performance significantly increases when we use the behavior-based dependency network effects in model (3), compared to the baseline model (1) and distance-based network effects model (2). Including both distance-based network effects and the behavior-based network effects slightly increases the R^2, however, the estimated coefficients for $\sum_j w_{ij}\tilde{v}_j$ are substantially larger (5 to 15 fold) than the coefficients of $\sum_j \delta_{ij}\tilde{v}_j$, indicating the significance of the dependency network effects.

5.3 Robustness against co-visit detection parameters

To test the robustness of the prediction power of the models using the dependency-based network effects, we test the adjusted R^2 of the models using dependency networks generated using different parameters for the co-visit detection algorithm. Similar to Supplementary Note 4.3, we test the regression models under 25 pairs of parameters ($T_s = [1, 2, 3, 5, unlimited]$ and $T_c = [1, 3, 6, 12, 24]$ hours). Figure 34 shows the adjusted R^2 values of the models using dependency networks generated with the corresponding co-visit detection parameters. Across all five metropolitan areas, we observe the general trend of the performance of the models increasing with longer maximum time differences and with smaller number of between-visit steps. This suggests that, for detecting the impact of the COVID-19 pandemic on businesses, we need to observe 1-step (thus direct) visitation patterns between POIs, regardless of the interval time between the entry and exit.

Supplementary Table 8: Linear regression models predicting the change in visits to POIs during the pandemic (2019 March - May) in New York. Variables were centered and standardized prior to analysis. Regression coefficients are reported, followed by coefficient estimate standard error in parentheses, estimate P value and a 95% confidence interval. Similar analyses are presented in Tables 9 - 52.

	Dependent variable: $\tilde{v}_i$ (Change in visits during the pandemic)			
	(1)	(2)	(3)	(4)
Constant	-40.9 (5.345)	-41.6 (5.314)	-41.7 (5.110)	-42.2 (5.089)
	P <0.001	P <0.001	P <0.001	P <0.001
	(-51.4, -30.4)	(-52.0, -31.1)	(-51.7, -31.7)	(-52.2, -32.2)
Distance effect $\sum_j \delta_{ij}\tilde{v}_j$		4.278 (0.085)		3.410 (0.082)
		P <0.001		P <0.001
		(4.110, 4.446)		(3.248, 3.571)
Dependency effect $\sum_j w_{ij}\tilde{v}_j$			14.59 (0.103)	14.26 (0.103)
			P <0.001	P <0.001
			(14.39, 14.79)	(14.06, 14.47)
Subcategory FE	Yes	Yes	Yes	Yes
PUMA FE	Yes	Yes	Yes	Yes
Observations	213,140	213,140	213,140	213,140
R^2	0.158	0.168	0.230	0.237
Adjusted R^2	0.157	0.167	0.230	0.236

Supplementary Table 9: Linear regression models predicting the change in visits to POIs during the pandemic (2019 March - May) in Boston.

	Dependent variable: $\tilde{v}_i$ *(Change in visits during the pandemic)*			
	(1)	(2)	(3)	(4)
Constant	-20.1 (6.512)	-22.1 (6.464)	-33.7 (6.253)	-34.9 (6.221)
	P =0.001	P <0.001	P <0.001	P <0.001
	(-32.9, -7.38)	(-34.8, -9.46)	(-46.0, -21.5)	(-47.1, -22.7)
Distance effect $\sum_j \delta_{ij}\tilde{v}_j$		4.919 (0.182)		3.946 (0.176)
		P <0.001		P <0.001
		(4.561, 5.277)		(3.600, 4.292)
Dependency effect $\sum_j w_{ij}\tilde{v}_j$			13.00 (0.201)	12.60 (0.200)
			P <0.001	P <0.001
			(12.60, 13.39)	(12.21, 13.00)
Subcategory FE	Yes	Yes	Yes	Yes
PUMA FE	Yes	Yes	Yes	Yes
Observations	48,848	48,848	48,848	48,848
R^2	0.145	0.158	0.213	0.221
Adjusted R^2	0.143	0.155	0.210	0.218

Supplementary Table 10: Linear regression models predicting the change in visits to POIs during the pandemic (2019 March - May) in Seattle.

	Dependent variable: $\tilde{v}_i$ *(Change in visits during the pandemic)*			
	(1)	(2)	(3)	(4)
Constant	-21.5 (10.58)	-22.2 (10.49)	-26.3 (10.06)	-26.6 (10.00)
	P =0.041	P =0.034	P =0.008	P =0.007
	(-42.3, -0.80)	(-42.7, -1.62)	(-46.0, -6.59)	(-46.3, -7.08)
Distance effect $\sum_j \delta_{ij}\tilde{v}_j$		5.021 (0.187)		3.993 (0.179)
		P <0.001		P <0.001
		(4.654, 5.389)		(3.642, 4.344)
Dependency effect $\sum_j w_{ij}\tilde{v}_j$			13.72 (0.205)	13.32 (0.205)
			P <0.001	P <0.001
			(13.32, 14.13)	(12.92, 13.72)
Subcategory FE	Yes	Yes	Yes	Yes
PUMA FE	Yes	Yes	Yes	Yes
Observations	41,451	41,451	41,451	41,451
R^2	0.168	0.182	0.249	0.258
Adjusted R^2	0.166	0.180	0.247	0.256

Supplementary Table 11: Linear regression models predicting the change in visits to POIs during the pandemic (2019 March - May) in Los Angeles.

	Dependent variable: $\tilde{v}_i$ (Change in visits during the pandemic)			
	(1)	(2)	(3)	(4)
Constant	-17.3 (4.820)	-17.8 (4.783)	-21.2 (4.554)	-21.4 (4.534)
	P <0.001	P <0.001	P <0.001	P <0.001
	(-26.8, -7.94)	(-27.2, -8.47)	(-30.1, -12.2)	(-30.3, -12.5)
Distance effect $\sum_j \delta_{ij}\tilde{v}_j$		4.860 (0.095)		3.531 (0.091)
		P <0.001		P <0.001
		(4.672, 5.047)		(3.353, 3.710)
Dependency effect $\sum_j w_{ij}\tilde{v}_j$			14.30 (0.100)	13.89 (0.100)
			P <0.001	P <0.001
			(14.10, 14.49)	(13.69, 14.08)
Subcategory FE	Yes	Yes	Yes	Yes
PUMA FE	Yes	Yes	Yes	Yes
Observations	168,385	168,385	168,385	168,385
R^2	0.137	0.150	0.229	0.236
Adjusted R^2	0.136	0.149	0.228	0.235

Supplementary Table 12: Linear regression models predicting the change in visits to POIs during the pandemic (2019 March - May) in Dallas.

	Dependent variable: $\tilde{v}_i$ (Change in visits during the pandemic)			
	(1)	(2)	(3)	(4)
Constant	3.944 (10.25)	1.362 (10.15)	-11.3 (9.730)	-12.6 (9.671)
	P =0.700	P =0.893	P =0.241	P =0.189
	(-16.1, 24.05)	(-18.5, 21.26)	(-30.4, 7.674)	(-31.6, 6.266)
Distance effect $\sum_j \delta_{ij}\tilde{v}_j$		5.985 (0.146)		4.427 (0.140)
		P <0.001		P <0.001
		(5.699, 6.272)		(4.152, 4.702)
Dependency effect $\sum_j w_{ij}\tilde{v}_j$			15.28 (0.160)	14.66 (0.160)
			P <0.001	P <0.001
			(14.96, 15.59)	(14.35, 14.98)
Subcategory FE	Yes	Yes	Yes	Yes
PUMA FE	Yes	Yes	Yes	Yes
Observations	81,457	81,457	81,457	81,457
R^2	0.140	0.158	0.227	0.236
Adjusted R^2	0.139	0.156	0.225	0.235

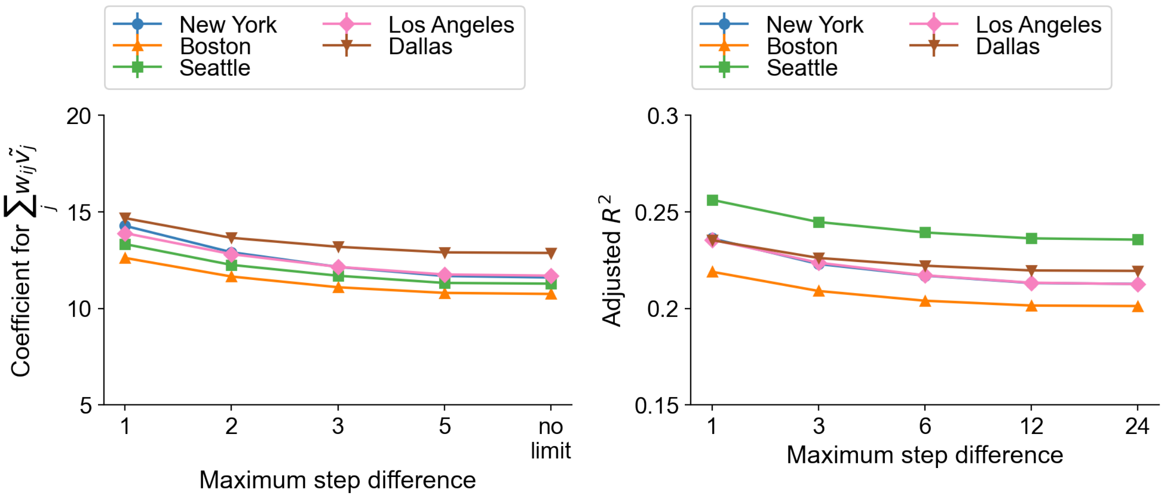

(a) Step difference parameter

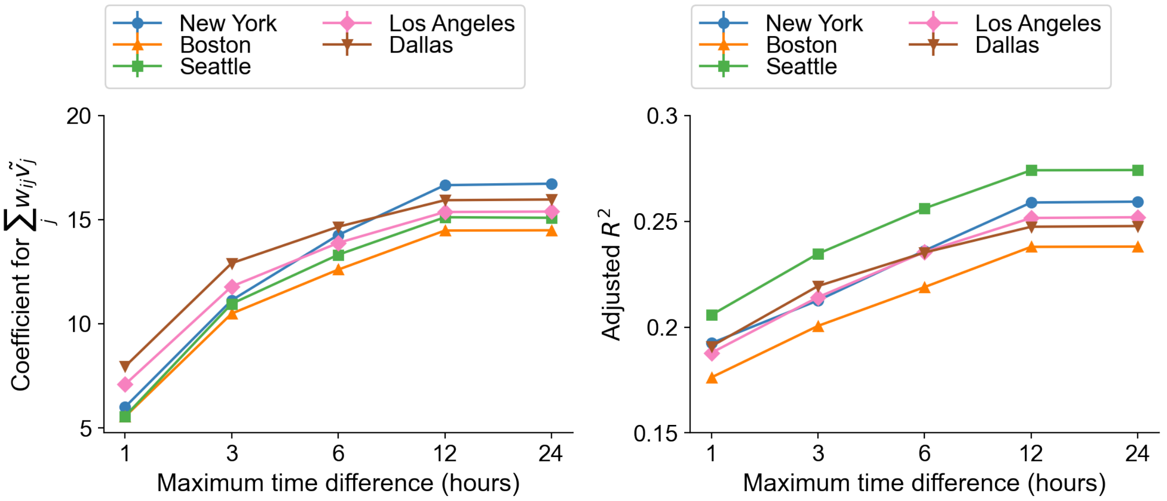

(b) Time difference parameter

Supplementary Figure 34: Regression results for dependency weights when using different co-visit detection parameters, continued for Los Angeles and Dallas. The error bars show the 95% confidence intervals for the coefficients. Except for the networks generated using maximum time difference of 1 hour, the results (adjusted R^2 and coefficient for Haversine distance) are relatively similar as the results in Table 3.

5.4 Results using changes in visits during different time periods

To test whether the behavior-based dependency network effects significantly improves the prediction performance of visitation losses to POIs regardless of the time period, we test the same regression models with dependent variables calculated from different periods during the pandemic. Tables 13 to 17 show the regression models for predicting the change of visits during 2020/6/1 and 2020/8/31, compared to the pre-pandemic period (2019/9/1 to 2019/11/30). Thus, the dependent variable is computed as:

$$\tilde{v}_i = \big(\frac{v_i^{2020June \sim 2020August}}{v_i^{2019September \sim 2019November}} - 1\big) * 100(\%) \tag{6}$$

In all metropolitan areas, the adjusted R^2 using the behavior-based dependency network effects (model 3) outperforms models 1 and 2 by 50% to 100%, showing substantial improvement in predictability. The regression coefficients for the behavior-based dependency network effects is significantly larger (30 fold) than the distance based effects, similar to the findings from the experiments in Tables 9 to 12.

Similarly, Tables 18 to 22 show the regression models for predicting the change of visits during 2020/9/1 and 2020/11/30, compared to the pre-pandemic period (2019/9/1 to 2019/11/30). In all metropolitan areas, the adjusted R^2 using the behavior-based dependency network effects (model 3) outperforms models 1 and 2 by 50% to 100%, showing substantial improvement in predictability. The regression coefficients for the behavior-based dependency network effects is significantly larger (30 fold) than the distance based effects, similar to the findings from the previous experiments. Overall the magnitude of the R^2 across all 4 models are smaller than those in the previous experiments. This suggests that the dependency network observed during one year prior to the dependent variable loses predictive power due to structural changes in the urban form. Nevertheless, experiments using change in visits during different time periods during the pandemic showed that the behavior-based dependency network effects substantially improve predictability compared to using the distance-based network effects.

5.5 Results on post-pandemic recovery using different time periods

In addition to testing the predictability of visitation losses to POIs during different time periods compared to pre-pandemic levels, we test the same regression models with dependent variables as the recovery of visits compared to the initial stages of the pandemic. The dependent variables are computed as:

$$\tilde{v}_i = \big(\frac{v_i^{2020June \sim 2020August}}{v_i^{2020March \sim 2020May}} - 1\big) * 100(\%) \tag{7}$$

and

$$\tilde{v}_i = \big(\frac{v_i^{2020September \sim 2020November}}{v_i^{2020March \sim 2020May}} - 1\big) * 100(\%) \tag{8}$$

Tables 23 to 27 show the regression models for predicting the recovery of visits during 2020/6/1 and 2020/8/31, compared to the initial stages of the pandemic (2020/3/1 to 2020/5/31). Similarly, Tables 28 to 32 show the regression models for predicting the recovery of visits during 2020/9/1 and 2020/11/30, compared to the initial stages of the pandemic (2020/3/1 to 2020/5/31). In all metropolitan areas, the adjusted R^2 using the behavior-based dependency network effects (model 3) outperforms

Supplementary Table 13: Linear regression models predicting the change in visits to POIs during the pandemic (2019 June - August) in New York.

	Dependent variable: $\tilde{v}_i$ *(Change in visits during the pandemic)*			
	(1)	(2)	(3)	(4)
Constant	-20.6 (17.00)	-21.1 (16.65)	-15.8 (15.66)	-16.3 (15.62)
	P =0.225	P =0.204	P =0.312	P =0.295
	(-53.9, 12.72)	(-53.7, 11.51)	(-46.5, 14.88)	(-46.9, 14.26)
Distance effect $\sum_j \delta_{ij}\tilde{v_j}$		26.41 (0.275)		10.12 (0.275)
		P <0.001		P <0.001
		(25.87, 26.95)		(9.583, 10.66)
Dependency effect $\sum_j w_{ij}\tilde{v_j}$			57.97 (0.297)	53.96 (0.316)
			P <0.001	P <0.001
			(57.39, 58.56)	(53.34, 54.58)
Subcategory FE	Yes	Yes	Yes	Yes
PUMA FE	Yes	Yes	Yes	Yes
Observations	213,140	213,140	213,140	213,140
R^2	0.072	0.111	0.213	0.218
Adjusted R^2	0.071	0.110	0.212	0.217

Supplementary Table 14: Linear regression models predicting the change in visits to POIs during the pandemic (2019 June - August) in Boston.

	Dependent variable: $\tilde{v}_i$ *(Change in visits during the pandemic)*			
	(1)	(2)	(3)	(4)
Constant	-19.6 (18.67)	-24.9 (18.22)	-33.7 (17.52)	-35.2 (17.38)
	P =0.291	P =0.170	P =0.054	P =0.042
	(-56.2, 16.92)	(-60.7, 10.72)	(-68.1, 0.588)	(-69.2, -1.13)
Distance effect $\sum_j \delta_{ij}\tilde{v_j}$		25.83 (0.519)		14.70 (0.521)
		P <0.001		P <0.001
		(24.81, 26.84)		(13.68, 15.72)
Dependency effect $\sum_j w_{ij}\tilde{v_j}$			44.40 (0.546)	39.43 (0.570)
			P <0.001	P <0.001
			(43.33, 45.47)	(38.32, 40.55)
Subcategory FE	Yes	Yes	Yes	Yes
PUMA FE	Yes	Yes	Yes	Yes
Observations	48,848	48,848	48,848	48,848
R^2	0.062	0.107	0.173	0.187
Adjusted R^2	0.059	0.104	0.171	0.185

Supplementary Table 15: Linear regression models predicting the change in visits to POIs during the pandemic (2019 June - August) in Seattle.

	Dependent variable: $\tilde{v}_i$ *(Change in visits during the pandemic)*			
	(1)	(2)	(3)	(4)
Constant	3.507 (16.98)	2.605 (16.81)	-1.16 (16.36)	-1.55 (16.27)
	P =0.836	P =0.876	P =0.943	P =0.924
	(-29.7, 36.79)	(-30.3, 35.55)	(-33.2, 30.90)	(-33.4, 30.34)
Distance effect $\sum_j \delta_{ij}\tilde{v}_j$		8.777 (0.303)		6.400 (0.296)
		P <0.001		P <0.001
		(8.183, 9.371)		(5.818, 6.981)
Dependency effect $\sum_j w_{ij}\tilde{v}_j$			19.09 (0.338)	17.98 (0.340)
			P <0.001	P <0.001
			(18.43, 19.76)	(17.31, 18.65)
Subcategory FE	Yes	Yes	Yes	Yes
PUMA FE	Yes	Yes	Yes	Yes
Observations	41,451	41,451	41,451	41,451
R^2	0.106	0.124	0.170	0.180
Adjusted R^2	0.104	0.121	0.168	0.177

Supplementary Table 16: Linear regression models predicting the change in visits to POIs during the pandemic (2019 June - August) in Los Angeles.

	Dependent variable: $\tilde{v}_i$ *(Change in visits during the pandemic)*			
	(1)	(2)	(3)	(4)
Constant	8.252 (7.296)	7.685 (7.219)	3.501 (6.885)	3.390 (6.857)
	P =0.258	P =0.287	P =0.611	P =0.621
	(-6.04, 22.55)	(-6.46, 21.83)	(-9.99, 16.99)	(-10.0, 16.83)
Distance effect $\sum_j \delta_{ij}\tilde{v}_j$		8.702 (0.145)		5.212 (0.140)
		P <0.001		P <0.001
		(8.417, 8.986)		(4.937, 5.488)
Dependency effect $\sum_j w_{ij}\tilde{v}_j$			21.46 (0.149)	20.42 (0.151)
			P <0.001	P <0.001
			(21.16, 21.75)	(20.13, 20.72)
Subcategory FE	Yes	Yes	Yes	Yes
PUMA FE	Yes	Yes	Yes	Yes
Observations	168,385	168,385	168,385	168,385
R^2	0.080	0.099	0.180	0.187
Adjusted R^2	0.079	0.098	0.179	0.186

Supplementary Table 17: Linear regression models predicting the change in visits to POIs during the pandemic (2019 June - August) in Dallas.

	Dependent variable: $\tilde{v}_i$ (Change in visits during the pandemic)			
	(1)	(2)	(3)	(4)
Constant	38.63 (18.73)	33.88 (18.61)	12.24 (18.16)	10.28 (18.11)
	P =0.039	P =0.068	P =0.500	P =0.570
	(1.920, 75.34)	(-2.60, 70.37)	(-23.3, 47.84)	(-25.2, 45.78)
Distance effect $\sum_j \delta_{ij}\tilde{v}_j$		8.472 (0.269)		5.673 (0.265)
		P <0.001		P <0.001
		(7.943, 9.001)		(5.152, 6.194)
Dependency effect $\sum_j w_{ij}\tilde{v}_j$			20.41 (0.283)	19.47 (0.285)
			P <0.001	P <0.001
			(19.85, 20.96)	(18.91, 20.03)
Subcategory FE	Yes	Yes	Yes	Yes
PUMA FE	Yes	Yes	Yes	Yes
Observations	81,457	81,457	81,457	81,457
R^2	0.062	0.073	0.118	0.123
Adjusted R^2	0.060	0.071	0.116	0.121

Supplementary Table 18: Linear regression models predicting the change in visits to POIs during the pandemic (2019 September - November) in New York.

	Dependent variable: $\tilde{v}_i$ (Change in visits during the pandemic)			
	(1)	(2)	(3)	(4)
Constant	-11.7 (9.700)	-12.4 (9.625)	-11.4 (9.413)	-11.9 (9.382)
	P =0.226	P =0.196	P =0.223	P =0.203
	(-30.7, 7.291)	(-31.2, 6.435)	(-29.9, 6.982)	(-30.3, 6.445)
Distance effect $\sum_j \delta_{ij}\tilde{v}_j$		9.426 (0.163)		6.109 (0.162)
		P <0.001		P <0.001
		(9.106, 9.747)		(5.791, 6.428)
Dependency effect $\sum_j w_{ij}\tilde{v}_j$			22.57 (0.196)	21.12 (0.199)
			P <0.001	P <0.001
			(22.18, 22.95)	(20.73, 21.51)
Subcategory FE	Yes	Yes	Yes	Yes
PUMA FE	Yes	Yes	Yes	Yes
Observations	213,140	213,140	213,140	213,140
R^2	0.081	0.095	0.135	0.140
Adjusted R^2	0.080	0.094	0.134	0.139

Supplementary Table 19: Linear regression models predicting the change in visits to POIs during the pandemic (2019 September - November) in Boston.

	Dependent variable: $\tilde{v}_i$ *(Change in visits during the pandemic)*			
	(1)	(2)	(3)	(4)
Constant	-13.4 (15.04)	-15.8 (14.98)	-19.8 (14.94)	-21.2 (14.90)
	P =0.370	P =0.288	P =0.184	P =0.153
	(-42.9, 16.02)	(-45.2, 13.47)	(-49.1, 9.456)	(-50.4, 7.955)
Distance effect $\sum_j \delta_{ij}\tilde{v}_j$		8.396 (0.427)		6.962 (0.429)
		P <0.001		P <0.001
		(7.559, 9.234)		(6.120, 7.804)
Dependency effect $\sum_j w_{ij}\tilde{v}_j$			11.95 (0.469)	10.84 (0.472)
			P <0.001	P <0.001
			(11.03, 12.87)	(9.915, 11.76)
Subcategory FE	Yes	Yes	Yes	Yes
PUMA FE	Yes	Yes	Yes	Yes
Observations	48,848	48,848	48,848	48,848
R^2	0.081	0.088	0.093	0.097
Adjusted R^2	0.078	0.085	0.090	0.095

Supplementary Table 20: Linear regression models predicting the change in visits to POIs during the pandemic (2019 September - November) in Seattle.

	Dependent variable: $\tilde{v}_i$ *(Change in visits during the pandemic)*			
	(1)	(2)	(3)	(4)
Constant	8.718 (16.05)	7.900 (15.92)	4.898 (15.56)	4.468 (15.48)
	P =0.587	P =0.619	P =0.752	P =0.772
	(-22.7, 40.18)	(-23.3, 39.11)	(-25.6, 35.40)	(-25.8, 34.82)
Distance effect $\sum_j \delta_{ij}\tilde{v}_j$		7.389 (0.286)		5.628 (0.281)
		P <0.001		P <0.001
		(6.828, 7.951)		(5.077, 6.179)
Dependency effect $\sum_j w_{ij}\tilde{v}_j$			16.64 (0.323)	15.80 (0.324)
			P <0.001	P <0.001
			(16.01, 17.27)	(15.17, 16.44)
Subcategory FE	Yes	Yes	Yes	Yes
PUMA FE	Yes	Yes	Yes	Yes
Observations	41,451	41,451	41,451	41,451
R^2	0.071	0.086	0.127	0.136
Adjusted R^2	0.069	0.083	0.125	0.133

Supplementary Table 21: Linear regression models predicting the change in visits to POIs during the pandemic (2019 September - November) in Los Angeles.

	Dependent variable: $\tilde{v}_i$ (Change in visits during the pandemic)			
	(1)	(2)	(3)	(4)
Constant	9.110 (7.274)	8.471 (7.214)	4.076 (7.055)	3.920 (7.024)
	P =0.210	P =0.240	P =0.563	P =0.576
	(-5.14, 23.36)	(-5.66, 22.61)	(-9.75, 17.90)	(-9.84, 17.68)
Distance effect $\sum_j \delta_{ij}\tilde{v}_j$		7.655 (0.144)		5.468 (0.142)
		P <0.001		P <0.001
		(7.371, 7.939)		(5.188, 5.749)
Dependency effect $\sum_j w_{ij}\tilde{v}_j$			15.62 (0.151)	14.69 (0.152)
			P <0.001	P <0.001
			(15.33, 15.92)	(14.39, 14.99)
Subcategory FE	Yes	Yes	Yes	Yes
PUMA FE	Yes	Yes	Yes	Yes
Observations	168,385	168,385	168,385	168,385
R^2	0.053	0.069	0.109	0.117
Adjusted R^2	0.052	0.068	0.108	0.116

Supplementary Table 22: Linear regression models predicting the change in visits to POIs during the pandemic (2019 September - November) in Dallas.

	Dependent variable: $\tilde{v}_i$ (Change in visits during the pandemic)			
	(1)	(2)	(3)	(4)
Constant	17.86 (19.67)	14.28 (19.58)	-4.65 (19.18)	-6.44 (19.14)
	P =0.363	P =0.466	P =0.808	P =0.736
	(-20.6, 56.42)	(-24.1, 52.67)	(-42.2, 32.94)	(-43.9, 31.07)
Distance effect $\sum_j \delta_{ij}\tilde{v}_j$		7.407 (0.282)		5.340 (0.278)
		P <0.001		P <0.001
		(6.852, 7.961)		(4.795, 5.886)
Dependency effect $\sum_j w_{ij}\tilde{v}_j$			18.95 (0.292)	18.28 (0.293)
			P <0.001	P <0.001
			(18.38, 19.53)	(17.70, 18.86)
Subcategory FE	Yes	Yes	Yes	Yes
PUMA FE	Yes	Yes	Yes	Yes
Observations	81,457	81,457	81,457	81,457
R^2	0.037	0.045	0.084	0.089
Adjusted R^2	0.035	0.043	0.083	0.087

models 1 and 2 by 50% to 100%, showing substantial improvement in predictability. The regression coefficients for the behavior-based dependency network effects is significantly larger than the distance based effects, similar to the findings from the previous experiments. Overall the magnitude of the R^2 across all 4 models are smaller than those in the previous experiments, suggesting that the recovery dynamics is less predictable compared to visitation losses as we saw in the previous results in Section 5.4. Nevertheless, experiments using the recovery in visits during different time periods during the pandemic showed that the behavior-based dependency network effects substantially improve predictability compared to using the distance-based network effects.

Supplementary Table 23: Linear regression models predicting the recovery of visits to POIs during the pandemic (2020 June - August) in New York compared to the initial stages of the pandemic (2020 March - May).

	Dependent variable: $\tilde{v}_i$ (Recovery of visits during the pandemic)			
	(1)	(2)	(3)	(4)
Constant	29.82 (26.09)	34.93 (25.97)	21.97 (25.16)	24.10 (25.14)
	P =0.253	P =0.178	P =0.382	P =0.337
	(-21.3, 80.97)	(-15.9, 85.83)	(-27.3, 71.28)	(-25.1, 73.38)
Distance effect $\sum_j \delta_{ij}\tilde{v}_j$		19.23 (0.429)		7.063 (0.428)
		P <0.001		P <0.001
		(18.39, 20.07)		(6.222, 7.903)
Dependency effect $\sum_j w_{ij}\tilde{v}_j$			56.12 (0.447)	54.29 (0.460)
			P <0.001	P <0.001
			(55.24, 56.99)	(53.39, 55.20)
Subcategory FE	Yes	Yes	Yes	Yes
PUMA FE	Yes	Yes	Yes	Yes
Observations	208,470	208,470	208,470	208,470
R^2	0.061	0.070	0.127	0.128
Adjusted R^2	0.060	0.069	0.126	0.127

5.6 Robustness of regression results when using different time periods to generate dependency networks

The robustness checks conducted in Supplementary Notes 3.1 and 4.4 showed high correlation in edge weights between different time periods. Nevertheless, in this section, we tested whether the time periods used to generate the behavior-based dependency networks affects the performance of the regression models. Tables 33 to 37 show the regression models for predicting the change of visits during 2020/3/1 and 2020/5/31, using the dependency network computed from data between 2019/1/1 and 2019/4/30.

In all metropolitan areas, the adjusted R^2 using the behavior-based dependency network effects (model 3) outperforms models 1 and 2 by around 50%, showing substantial improvement in predictability. The regression coefficients for the behavior-based dependency network effects is signifi-

Supplementary Table 24: Linear regression models predicting the recovery of visits to POIs during the pandemic (2020 June - August) in Boston compared to the initial stages of the pandemic (2020 March - May).

	Dependent variable: $\tilde{v}_i$ (Recovery of visits during the pandemic)			
	(1)	(2)	(3)	(4)
Constant	9.405 (43.92)	20.06 (43.73)	45.72 (42.78)	45.36 (42.78)
	P =0.830	P =0.646	P =0.285	P =0.289
	(-76.6, 95.50)	(-65.6, 105.7)	(-38.1, 129.5)	(-38.4, 129.2)
Distance effect $\sum_j \delta_{ij}\tilde{v}_j$		25.71 (1.259)		-2.29 (1.373)
		P <0.001		P =0.095
		(23.24, 28.18)		(-4.98, 0.400)
Dependency effect $\sum_j w_{ij}\tilde{v}_j$			64.42 (1.274)	65.47 (1.421)
			P <0.001	P <0.001
			(61.93, 66.92)	(62.69, 68.26)
Subcategory FE	Yes	Yes	Yes	Yes
PUMA FE	Yes	Yes	Yes	Yes
Observations	47,276	47,276	47,276	47,276
R^2	0.042	0.050	0.091	0.091
Adjusted R^2	0.039	0.048	0.089	0.089

cantly larger (around 5 fold) than the distance based effects, similar to the findings from the previous experiments.

Similarly, Tables 38 to 42 show the regression models for predicting the change of visits during 2020/3/1 and 2020/5/31, using the dependency network computed from data between 2019/5/1 and 2019/8/31. Again, in all metropolitan areas, the adjusted R^2 using the behavior-based dependency network effects (model 3) outperforms models 1 and 2 by around 50%, showing the robustness against the choice of time period to generated the dependency networks. In summary, the results on the predictability of shocks using the behavior-based dependency networks are robust against the choice of the time periods used to generate the dependency networks.

5.7 Robustness of regression results when using only short non-work stays

In this section, we test whether the behavior-based dependency network generated using only short (i.e., non-work) stays yields similar performance metrics in the regression models. Tables 43 to 47 show the regression models for predicting the change of visits during 2020/3/1 and 2020/5/31, using the dependency network computed with stays shorter than 4 hours. In all metropolitan areas, the adjusted R^2 using the behavior-based dependency network effects (model 3) outperforms models 1 and 2, showing improvement in predictability. The regression coefficients for the behavior-based dependency network effects are significantly larger (around 5 fold) than the distance based effects, similar to the findings from the previous experiments. This results suggest that our estimation results are robust against the limiting the analysis to the use of short non-work trips.

Supplementary Table 25: Linear regression models predicting the recovery of visits to POIs during the pandemic (2020 June - August) in Seattle compared to the initial stages of the pandemic (2020 March - May).

	Dependent variable: $\tilde{v}_i$ (Recovery of visits during the pandemic)			
	(1)	(2)	(3)	(4)
Constant	11.98 (40.83)	13.94 (40.72)	35.70 (38.02)	35.42 (38.00)
	P =0.769	P =0.732	P =0.347	P =0.351
	(-68.0, 92.01)	(-65.8, 93.75)	(-38.8, 110.2)	(-39.0, 109.9)
Distance effect $\sum_j \delta_{ij}\tilde{v}_j$		10.95 (0.748)		-5.12 (0.729)
		P <0.001		P <0.001
		(9.485, 12.42)		(-6.55, -3.69)
Dependency effect $\sum_j w_{ij}\tilde{v}_j$			59.46 (0.759)	61.05 (0.791)
			P <0.001	P <0.001
			(57.97, 60.95)	(59.50, 62.60)
Subcategory FE	Yes	Yes	Yes	Yes
PUMA FE	Yes	Yes	Yes	Yes
Observations	40,306	40,306	40,306	40,306
R^2	0.079	0.084	0.201	0.202
Adjusted R^2	0.077	0.081	0.199	0.200

5.8 Case study on school closure period

To further test the effectiveness of using the dependency network for predicting the cascade of shocks, we used school holidays as a separate case study. The assumption is that, places that are connected to schools (in our case, college places) tend to have an additional decrease in visits than other places that have less dependency on college POIs. To compute the change in visits, we used the school semester period (September to November 2019) as the post period and the summer break period (June to August 2019) as the pre- period.

$$\tilde{v}_i = \big(\frac{v_i^{2019September \sim 2019November}}{v_i^{2019June \sim 2019August}} - 1\big) * 100(\%) \tag{9}$$

Figure 35 shows the changes in visits to POIs in the five cities during the fall semester period compared to the summer break period. As the right panels clearly show, College POIs have a significant (25% to 60%) increase in the number of visits during the semester compared to the breaks. The same model specification was used to test the effectiveness of using the dependency network. Tables 48 to 52 show the regression results. Although the predictability of the change in visits is generally lower than the pandemic results (due to the smaller impacts of summer holidays compared to the pandemic), in all five cities, the model performance is highest when we use the behavior-based dependency network compared to when we use the distance-based null network.

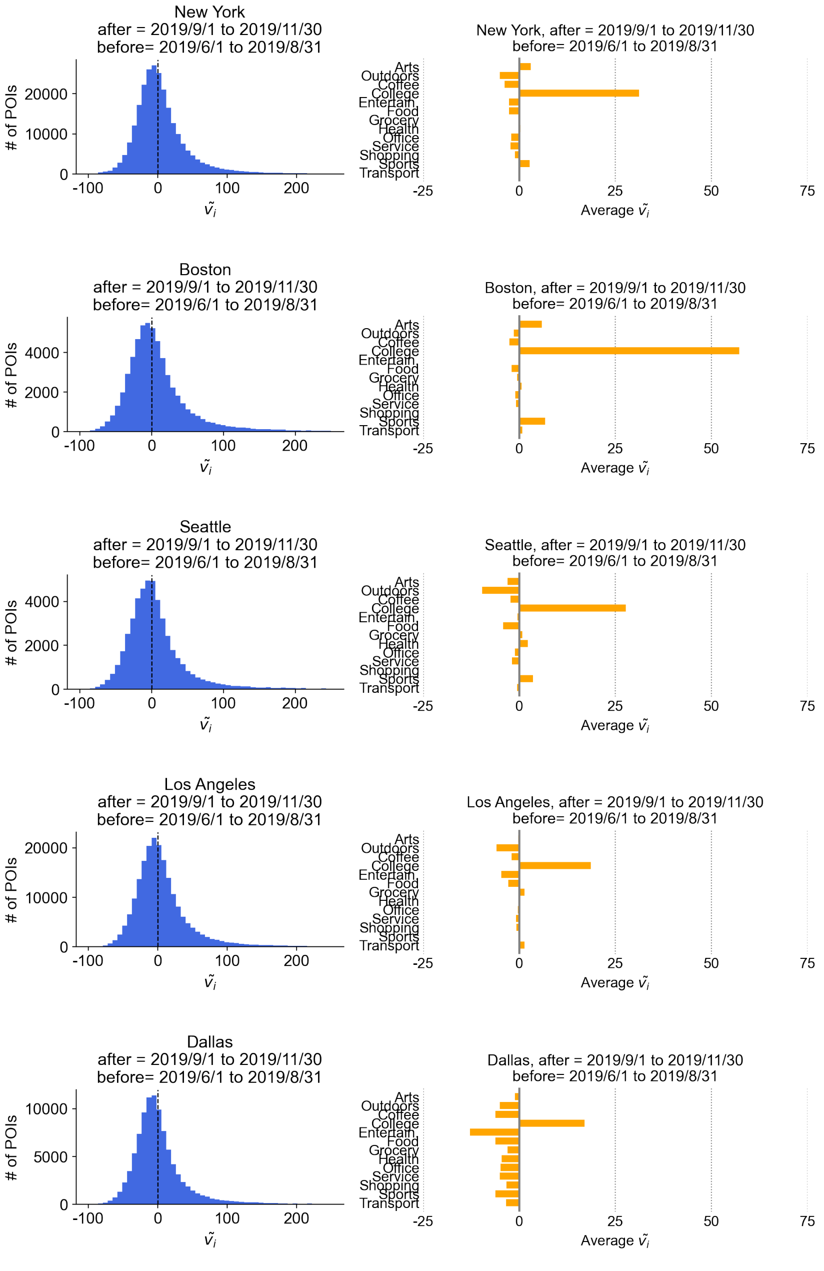

Supplementary Figure 35: Change in visits to POIs in New York, Boston, Seattle, Los Angeles, and Dallas during the fall semester period. The left panels show the distribution of the change in visits, and the right panels show the average effects per POI category.

Supplementary Table 26: Linear regression models predicting the recovery of visits to POIs during the pandemic (2020 June - August) in Los Angeles compared to the initial stages of the pandemic (2020 March - May).

	Dependent variable: $\tilde{v}_i$ *(Recovery of visits during the pandemic)*			
	(1)	(2)	(3)	(4)
Constant	30.90 (14.46)	31.81 (14.44)	41.36 (13.59)	41.00 (13.58)
	P =0.032	P =0.027	P =0.002	P =0.002
	(2.559, 59.24)	(3.508, 60.13)	(14.71, 68.01)	(14.37, 67.64)
Distance effect $\sum_j \delta_{ij}\tilde{v}_j$		5.801 (0.292)		-3.65 (0.282)
		P <0.001		P <0.001
		(5.228, 6.374)		(-4.21, -3.10)
Dependency effect $\sum_j w_{ij}\tilde{v}_j$			43.64 (0.297)	44.55 (0.305)
			P <0.001	P <0.001
			(43.05, 44.22)	(43.95, 45.15)
Subcategory FE	Yes	Yes	Yes	Yes
PUMA FE	Yes	Yes	Yes	Yes
Observations	163,546	163,546	163,546	163,546
R^2	0.060	0.063	0.170	0.171
Adjusted R^2	0.059	0.062	0.169	0.170

Supplementary Table 27: Linear regression models predicting the recovery of visits to POIs during the pandemic (2020 June - August) in Dallas compared to the initial stages of the pandemic (2020 March - May).

	Dependent variable: $\tilde{v_i}$ (Recovery of visits during the pandemic)			
	(1)	(2)	(3)	(4)
Constant	120.5 (39.09)<br>P =0.002<br>(43.95, 197.2)	120.2 (39.09)<br>P =0.002<br>(43.64, 196.8)	96.69 (38.61)<br>P =0.012<br>(21.01, 172.3)	96.92 (38.61)<br>P =0.012<br>(21.24, 172.6)
Distance effect $\sum_j \delta_{ij}\tilde{v_j}$		1.290 (0.572)<br>P =0.024<br>(0.167, 2.413)		-1.68 (0.569)<br>P =0.003<br>(-2.80, -0.56)
Dependency effect $\sum_j w_{ij}\tilde{v_j}$			28.48 (0.635)<br>P <0.001<br>(27.23, 29.72)	28.70 (0.639)<br>P <0.001<br>(27.45, 29.95)
Subcategory FE	Yes	Yes	Yes	Yes
PUMA FE	Yes	Yes	Yes	Yes
Observations	80,495	80,495	80,495	80,495
R^2	0.030	0.031	0.054	0.054
Adjusted R^2	0.029	0.029	0.052	0.052

Supplementary Table 28: Linear regression models predicting the recovery of visits to POIs during the pandemic (2020 September - November) in New York compared to the initial stages of the pandemic (2020 March - May).

	Dependent variable: $\tilde{v_i}$ (Recovery of visits during the pandemic)			
	(1)	(2)	(3)	(4)
Constant	36.96 (25.89)<br>P =0.153<br>(-13.7, 87.72)	40.34 (25.84)<br>P =0.118<br>(-10.3, 91.00)	27.65 (25.08)<br>P =0.270<br>(-21.5, 76.82)	28.96 (25.08)<br>P =0.248<br>(-20.1, 78.12)
Distance effect $\sum_j \delta_{ij}\tilde{v_j}$		12.51 (0.427)<br>P <0.001<br>(11.67, 13.35)		4.313 (0.420)<br>P <0.001<br>(3.488, 5.137)
Dependency effect $\sum_j w_{ij}\tilde{v_j}$			51.93 (0.444)<br>P <0.001<br>(51.06, 52.80)	51.14 (0.450)<br>P <0.001<br>(50.25, 52.02)
Subcategory FE	Yes	Yes	Yes	Yes
PUMA FE	Yes	Yes	Yes	Yes
Observations	207,780	207,780	207,780	207,780
R^2	0.084	0.087	0.140	0.141
Adjusted R^2	0.083	0.086	0.139	0.140

Supplementary Table 29: Linear regression models predicting the recovery of visits to POIs during the pandemic (2020 September - November) in Boston compared to the initial stages of the pandemic (2020 March - May).

	Dependent variable: $\tilde{v}_i$ *(Recovery of visits during the pandemic)*			
	(1)	(2)	(3)	(4)
Constant	15.46 (65.55)	20.77 (65.51)	49.89 (64.67)	49.30 (64.67)
	P =0.813	P =0.751	P =0.440	P =0.445
	(-113., 143.9)	(-107., 149.1)	(-76.8, 176.6)	(-77.4, 176.0)
Distance effect $\sum_j \delta_{ij}\tilde{v}_j$		15.19 (1.872)		-2.76 (1.917)
		P <0.001		P =0.149
		(11.52, 18.86)		(-6.52, 0.997)
Dependency effect $\sum_j w_{ij}\tilde{v}_j$			69.42 (1.922)	70.19 (1.993)
			P <0.001	P <0.001
			(65.65, 73.19)	(66.28, 74.09)
Subcategory FE	Yes	Yes	Yes	Yes
PUMA FE	Yes	Yes	Yes	Yes
Observations	47,657	47,657	47,657	47,657
R^2	0.032	0.034	0.058	0.058
Adjusted R^2	0.030	0.031	0.055	0.055

Supplementary Table 30: Linear regression models predicting the recovery of visits to POIs during the pandemic (2020 September - November) in Seattle compared to the initial stages of the pandemic (2020 March - May).

	Dependent variable: $\tilde{v}_i$ *(Recovery of visits during the pandemic)*			
	(1)	(2)	(3)	(4)
Constant	10.74 (50.72)	13.34 (50.57)	39.90 (47.80)	39.76 (47.79)
	P =0.832	P =0.791	P =0.403	P =0.405
	(-88.6, 110.1)	(-85.7, 112.4)	(-53.7, 133.6)	(-53.9, 133.4)
Distance effect $\sum_j \delta_{ij}\tilde{v}_j$		14.34 (0.933)		-2.46 (0.914)
		P <0.001		P =0.006
		(12.51, 16.17)		(-4.25, -0.67)
Dependency effect $\sum_j w_{ij}\tilde{v}_j$			69.43 (0.975)	70.16 (1.011)
			P <0.001	P <0.001
			(67.52, 71.35)	(68.18, 72.14)
Subcategory FE	Yes	Yes	Yes	Yes
PUMA FE	Yes	Yes	Yes	Yes
Observations	40,318	40,318	40,318	40,318
R^2	0.087	0.092	0.189	0.189
Adjusted R^2	0.084	0.089	0.187	0.187

Supplementary Table 31: Linear regression models predicting the recovery of visits to POIs during the pandemic (2020 September - November) in Los Angeles compared to the initial stages of the pandemic (2020 March - May).

	Dependent variable: $\tilde{v}_i$ *(Recovery of visits during the pandemic)*			
	(1)	(2)	(3)	(4)
Constant	30.20 (21.46)	31.94 (21.46)	42.73 (20.55)	41.74 (20.54)
	P =0.159	P =0.136	P =0.037	P =0.042
	(-11.8, 72.28)	(-10.1, 74.00)	(2.451, 83.01)	(1.473, 82.02)
Distance effect $\sum_j \delta_{ij}\tilde{v}_j$		5.380 (0.430)		-3.53 (0.418)
		P <0.001		P <0.001
		(4.536, 6.224)		(-4.35, -2.71)
Dependency effect $\sum_j w_{ij}\tilde{v}_j$			55.45 (0.456)	56.14 (0.463)
			P <0.001	P <0.001
			(54.56, 56.35)	(55.23, 57.04)
Subcategory FE	Yes	Yes	Yes	Yes
PUMA FE	Yes	Yes	Yes	Yes
Observations	162,331	162,331	162,331	162,331
R^2	0.056	0.057	0.135	0.136
Adjusted R^2	0.055	0.056	0.134	0.135

Supplementary Table 32: Linear regression models predicting the recovery of visits to POIs during the pandemic (2020 September - November) in Dallas compared to the initial stages of the pandemic (2020 March - May).

	Dependent variable: $\tilde{v}_i$ *(Recovery of visits during the pandemic)*			
	(1)	(2)	(3)	(4)
Constant	157.1 (64.10)	156.2 (64.08)	136.8 (63.55)	136.7 (63.55)
	P =0.014	P =0.014	P =0.031	P =0.031
	(31.55, 282.8)	(30.68, 281.8)	(12.25, 261.3)	(12.18, 261.3)
Distance effect $\sum_j \delta_{ij}\tilde{v}_j$		6.391 (0.930)		1.314 (0.933)
		P <0.001		P =0.158
		(4.567, 8.216)		(-0.51, 3.144)
Dependency effect $\sum_j w_{ij}\tilde{v}_j$			37.09 (0.989)	36.88 (1.000)
			P <0.001	P <0.001
			(35.15, 39.03)	(34.92, 38.84)
Subcategory FE	Yes	Yes	Yes	Yes
PUMA FE	Yes	Yes	Yes	Yes
Observations	80,693	80,693	80,693	80,693
R^2	0.021	0.021	0.037	0.037
Adjusted R^2	0.019	0.019	0.036	0.036

Supplementary Table 33: Linear regression models predicting the change in visits to POIs during the pandemic (2019 September - November) in New York when using dependency network generated from data between 2019 January to April.

	Dependent variable: $\tilde{v}_i$ *(Change in visits during the pandemic)*			
	(1)	(2)	(3)	(4)
Constant	-38.3 (5.251)	-38.9 (5.222)	-40.3 (5.033)	-40.8 (5.009)
	P <0.001	P <0.001	P <0.001	P <0.001
	(-48.6, -28.0)	(-49.1, -28.6)	(-50.2, -30.5)	(-50.7, -31.0)
Distance effect $\sum_j \delta_{ij}\tilde{v}_j$		4.089 (0.084)		3.614 (0.081)
		P <0.001		P <0.001
		(3.923, 4.255)		(3.455, 3.774)
Dependency effect $\sum_j w_{ij}\tilde{v}_j$			13.63 (0.100)	13.44 (0.100)
			P <0.001	P <0.001
			(13.43, 13.83)	(13.24, 13.63)
Subcategory FE	Yes	Yes	Yes	Yes
PUMA FE	Yes	Yes	Yes	Yes
Observations	206,108	206,108	206,108	206,108
R^2	0.166	0.175	0.234	0.241
Adjusted R^2	0.165	0.174	0.233	0.240

Supplementary Table 34: Linear regression models predicting the change in visits to POIs during the pandemic (2019 September - November) in Boston when using dependency network generated from data between 2019 January to April.

	Dependent variable: $\tilde{v}_i$ *(Change in visits during the pandemic)*			
	(1)	(2)	(3)	(4)
Constant	-22.0 (6.518)	-24.0 (6.472)	-33.1 (6.277)	-34.6 (6.242)
	P <0.001	P <0.001	P <0.001	P <0.001
	(-34.8, -9.30)	(-36.7, -11.3)	(-45.5, -20.8)	(-46.8, -22.3)
Distance effect $\sum_j \delta_{ij}\tilde{v}_j$		4.779 (0.185)		4.102 (0.178)
		P <0.001		P <0.001
		(4.416, 5.142)		(3.751, 4.452)
Dependency effect $\sum_j w_{ij}\tilde{v}_j$			12.30 (0.202)	12.00 (0.201)
			P <0.001	P <0.001
			(11.90, 12.69)	(11.61, 12.40)
Subcategory FE	Yes	Yes	Yes	Yes
PUMA FE	Yes	Yes	Yes	Yes
Observations	46,643	46,643	46,643	46,643
R^2	0.147	0.159	0.210	0.219
Adjusted R^2	0.145	0.157	0.208	0.217

Supplementary Table 35: Linear regression models predicting the change in visits to POIs during the pandemic (2019 September - November) in Seattle when using dependency network generated from data between 2019 January to April.

	Dependent variable: $\tilde{v}_i$ *(Change in visits during the pandemic)*			
	(1)	(2)	(3)	(4)
Constant	-27.2 (11.03)	-27.5 (10.94)	-30.1 (10.52)	-30.3 (10.46)
	P =0.013	P =0.011	P =0.004	P =0.003
	(-48.8, -5.58)	(-49.0, -6.11)	(-50.7, -9.47)	(-50.8, -9.80)
Distance effect $\sum_j \delta_{ij}\tilde{v}_j$		4.856 (0.191)		3.937 (0.183)
		P <0.001		P <0.001
		(4.481, 5.230)		(3.577, 4.296)
Dependency effect $\sum_j w_{ij}\tilde{v}_j$			13.12 (0.211)	12.75 (0.210)
			P <0.001	P <0.001
			(12.71, 13.53)	(12.34, 13.16)
Subcategory FE	Yes	Yes	Yes	Yes
PUMA FE	Yes	Yes	Yes	Yes
Observations	39,582	39,582	39,582	39,582
R^2	0.173	0.186	0.247	0.256
Adjusted R^2	0.171	0.184	0.245	0.253

Supplementary Table 36: Linear regression models predicting the change in visits to POIs during the pandemic (2019 September - November) in Los Angeles when using dependency network generated from data between 2019 January to April.

	Dependent variable: $\tilde{v}_i$ *(Change in visits during the pandemic)*			
	(1)	(2)	(3)	(4)
Constant	-18.6 (4.828)	-19.3 (4.794)	-20.9 (4.563)	-21.3 (4.542)
	P <0.001	P <0.001	P <0.001	P <0.001
	(-28.1, -9.23)	(-28.7, -9.91)	(-29.8, -11.9)	(-30.2, -12.4)
Distance effect $\sum_j \delta_{ij}\tilde{v}_j$		4.651 (0.097)		3.561 (0.092)
		P <0.001		P <0.001
		(4.460, 4.841)		(3.380, 3.743)
Dependency effect $\sum_j w_{ij}\tilde{v}_j$			14.26 (0.102)	13.91 (0.102)
			P <0.001	P <0.001
			(14.05, 14.46)	(13.71, 14.11)
Subcategory FE	Yes	Yes	Yes	Yes
PUMA FE	Yes	Yes	Yes	Yes
Observations	162,861	162,861	162,861	162,861
R^2	0.138	0.150	0.230	0.237
Adjusted R^2	0.137	0.149	0.229	0.236

Supplementary Table 37: Linear regression models predicting the change in visits to POIs during the pandemic (2019 September - November) in Dallas when using dependency network generated from data between 2019 January to April.

	Dependent variable: $\tilde{v}_i$ (Change in visits during the pandemic)			
	(1)	(2)	(3)	(4)
Constant	7.027 (10.15)<br>P =0.488<br>(-12.8, 26.93)	4.521 (10.05)<br>P =0.652<br>(-15.1, 24.22)	-8.57 (9.647)<br>P =0.374<br>(-27.4, 10.33)	-9.93 (9.585)<br>P =0.300<br>(-28.7, 8.853)
Distance effect $\sum_j \delta_{ij}\tilde{v}_j$		5.827 (0.147)<br>P <0.001<br>(5.538, 6.117)		4.493 (0.141)<br>P <0.001<br>(4.216, 4.771)
Dependency effect $\sum_j w_{ij}\tilde{v}_j$			14.91 (0.162)<br>P <0.001<br>(14.59, 15.23)	14.37 (0.161)<br>P <0.001<br>(14.05, 14.68)
Subcategory FE	Yes	Yes	Yes	Yes
PUMA FE	Yes	Yes	Yes	Yes
Observations	78,222	78,222	78,222	78,222
R^2	0.145	0.161	0.228	0.238
Adjusted R^2	0.143	0.160	0.227	0.237

Supplementary Table 38: Linear regression models predicting the change in visits to POIs during the pandemic (2019 September - November) in New York when using dependency network generated from data between 2019 May to August.

	Dependent variable: $\tilde{v}_i$ (Change in visits during the pandemic)			
	(1)	(2)	(3)	(4)
Constant	-38.2 (5.249)<br>P <0.001<br>(-48.5, -28.0)	-38.7 (5.229)<br>P <0.001<br>(-49.0, -28.5)	-40.5 (5.016)<br>P <0.001<br>(-50.4, -30.7)	-40.9 (5.002)<br>P <0.001<br>(-50.7, -31.1)
Distance effect $\sum_j \delta_{ij}\tilde{v}_j$		3.398 (0.085)<br>P <0.001<br>(3.231, 3.565)		2.733 (0.081)<br>P <0.001<br>(2.573, 2.893)
Dependency effect $\sum_j w_{ij}\tilde{v}_j$			14.70 (0.104)<br>P <0.001<br>(14.50, 14.91)	14.50 (0.103)<br>P <0.001<br>(14.30, 14.70)
Subcategory FE	Yes	Yes	Yes	Yes
PUMA FE	Yes	Yes	Yes	Yes
Observations	210,540	210,540	210,540	210,540
R^2	0.164	0.170	0.236	0.240
Adjusted R^2	0.163	0.169	0.235	0.240

Supplementary Table 39: Linear regression models predicting the change in visits to POIs during the pandemic (2019 September - November) in Boston when using dependency network generated from data between 2019 May to August.

	Dependent variable: $\tilde{v}_i$ *(Change in visits during the pandemic)*			
	(1)	(2)	(3)	(4)
Constant	-19.8 (6.580)	-21.4 (6.551)	-33.8 (6.324)	-34.9 (6.302)
	P =0.002	P =0.001	P <0.001	P <0.001
	(-32.7, -6.94)	(-34.2, -8.56)	(-46.2, -21.4)	(-47.3, -22.6)
Distance effect $\sum_j \delta_{ij}\tilde{v}_j$		3.816 (0.185)		3.244 (0.178)
		P <0.001		P <0.001
		(3.452, 4.180)		(2.894, 3.594)
Dependency effect $\sum_j w_{ij}\tilde{v}_j$			13.20 (0.208)	13.00 (0.207)
			P <0.001	P <0.001
			(12.79, 13.61)	(12.60, 13.41)
Subcategory FE	Yes	Yes	Yes	Yes
PUMA FE	Yes	Yes	Yes	Yes
Observations	47,995	47,995	47,995	47,995
R^2	0.146	0.153	0.212	0.217
Adjusted R^2	0.144	0.151	0.210	0.215

Supplementary Table 40: Linear regression models predicting the change in visits to POIs during the pandemic (2019 September - November) in Seattle when using dependency network generated from data between 2019 May to August.

	Dependent variable: $\tilde{v}_i$ *(Change in visits during the pandemic)*			
	(1)	(2)	(3)	(4)
Constant	-21.9 (10.61)	-22.5 (10.53)	-26.3 (10.07)	-26.6 (10.03)
	P =0.038	P =0.032	P =0.008	P =0.007
	(-42.8, -1.19)	(-43.1, -1.85)	(-46.0, -6.58)	(-46.2, -6.95)
Distance effect $\sum_j \delta_{ij}\tilde{v}_j$		4.667 (0.190)		3.622 (0.181)
		P <0.001		P <0.001
		(4.294, 5.040)		(3.265, 3.978)
Dependency effect $\sum_j w_{ij}\tilde{v}_j$			14.01 (0.210)	13.64 (0.210)
			P <0.001	P <0.001
			(13.60, 14.42)	(13.22, 14.05)
Subcategory FE	Yes	Yes	Yes	Yes
PUMA FE	Yes	Yes	Yes	Yes
Observations	40,800	40,800	40,800	40,800
R^2	0.172	0.184	0.253	0.260
Adjusted R^2	0.169	0.181	0.251	0.258

Supplementary Table 41: Linear regression models predicting the change in visits to POIs during the pandemic (2019 September - November) in Los Angeles when using dependency network generated from data between 2019 May to August.

	Dependent variable: $\tilde{v}_i$ *(Change in visits during the pandemic)*			
	(1)	(2)	(3)	(4)
Constant	-17.6 (4.839)	-17.8 (4.806)	-20.5 (4.561)	-20.6 (4.543)
	P <0.001	P <0.001	P <0.001	P <0.001
	(-27.1, -8.21)	(-27.3, -8.46)	(-29.5, -11.6)	(-29.5, -11.7)
Distance effect $\sum_j \delta_{ij}\tilde{v}_j$		4.597 (0.096)		3.372 (0.091)
		P <0.001		P <0.001
		(4.407, 4.787)		(3.192, 3.552)
Dependency effect $\sum_j w_{ij}\tilde{v}_j$			14.72 (0.102)	14.37 (0.102)
			P <0.001	P <0.001
			(14.52, 14.92)	(14.17, 14.57)
Subcategory FE	Yes	Yes	Yes	Yes
PUMA FE	Yes	Yes	Yes	Yes
Observations	166,292	166,292	166,292	166,292
R^2	0.136	0.148	0.233	0.239
Adjusted R^2	0.135	0.147	0.232	0.238

Supplementary Table 42: Linear regression models predicting the change in visits to POIs during the pandemic (2019 September - November) in Dallas when using dependency network generated from data between 2019 May to August.

	Dependent variable: $\tilde{v}_i$ *(Change in visits during the pandemic)*			
	(1)	(2)	(3)	(4)
Constant	8.334 (10.10)	5.849 (10.01)	-8.60 (9.581)	-9.90 (9.530)
	P =0.409	P =0.559	P =0.369	P =0.298
	(-11.4, 28.13)	(-13.7, 25.48)	(-27.3, 10.17)	(-28.5, 8.771)
Distance effect $\sum_j \delta_{ij}\tilde{v}_j$		5.621 (0.149)		4.216 (0.143)
		P <0.001		P <0.001
		(5.327, 5.914)		(3.935, 4.497)
Dependency effect $\sum_j w_{ij}\tilde{v}_j$			15.68 (0.164)	15.16 (0.165)
			P <0.001	P <0.001
			(15.36, 16.01)	(14.84, 15.49)
Subcategory FE	Yes	Yes	Yes	Yes
PUMA FE	Yes	Yes	Yes	Yes
Observations	80,431	80,431	80,431	80,431
R^2	0.141	0.156	0.228	0.236
Adjusted R^2	0.140	0.154	0.227	0.235

Supplementary Table 43: Linear regression models predicting the change in visits to POIs during the pandemic (2019 September - November) in New York when using dependency network generated from stays shorter than 4 hours.

	Dependent variable: $\tilde{v_i}$ *(Change in visits during the pandemic)*			
	(1)	(2)	(3)	(4)
Constant	-41.1 (5.230)	-41.8 (5.200)	-42.7 (4.943)	-43.2 (4.928)
	P <0.001	P <0.001	P <0.001	P <0.001
	(-51.3, -30.8)	(-52.0, -31.6)	(-52.3, -33.0)	(-52.8, -33.5)
Distance effect $\sum_j \delta_{ij}\tilde{v_j}$		4.217 (0.084)		2.896 (0.080)
		P <0.001		P <0.001
		(4.050, 4.383)		(2.738, 3.055)
Dependency effect $\sum_j w_{ij}\tilde{v_j}$			16.66 (0.105)	16.26 (0.106)
			P <0.001	P <0.001
			(16.45, 16.87)	(16.05, 16.46)
Subcategory FE	Yes	Yes	Yes	Yes
PUMA FE	Yes	Yes	Yes	Yes
Observations	208,329	208,329	208,329	208,329
R^2	0.165	0.174	0.254	0.258
Adjusted R^2	0.164	0.173	0.253	0.257

Supplementary Table 44: Linear regression models predicting the change in visits to POIs during the pandemic (2019 September - November) in Boston when using dependency network generated from stays shorter than 4 hours.

	Dependent variable: $\tilde{v_i}$ *(Change in visits during the pandemic)*			
	(1)	(2)	(3)	(4)
Constant	-20.6 (6.562)	-22.6 (6.515)	-36.6 (6.261)	-37.5 (6.236)
	P =0.001	P <0.001	P <0.001	P <0.001
	(-33.5, -7.81)	(-35.4, -9.88)	(-48.9, -24.3)	(-49.7, -25.2)
Distance effect $\sum_j \delta_{ij}\tilde{v_j}$		4.844 (0.183)		3.456 (0.176)
		P <0.001		P <0.001
		(4.484, 5.203)		(3.110, 3.803)
Dependency effect $\sum_j w_{ij}\tilde{v_j}$			14.16 (0.205)	13.68 (0.206)
			P <0.001	P <0.001
			(13.76, 14.56)	(13.28, 14.09)
Subcategory FE	Yes	Yes	Yes	Yes
PUMA FE	Yes	Yes	Yes	Yes
Observations	47,466	47,466	47,466	47,466
R^2	0.148	0.160	0.225	0.231
Adjusted R^2	0.145	0.158	0.223	0.229

Supplementary Table 45: Linear regression models predicting the change in visits to POIs during the pandemic (2019 September - November) in Seattle when using dependency network generated from stays shorter than 4 hours.

	Dependent variable: $\tilde{v}_i$ *(Change in visits during the pandemic)*			
	(1)	(2)	(3)	(4)
Constant	-21.7 (10.50)	-22.4 (10.41)	-28.8 (9.905)	-29.0 (9.860)
	P =0.038	P =0.031	P =0.003	P =0.003
	(-42.3, -1.19)	(-42.8, -2.03)	(-48.2, -9.38)	(-48.3, -9.71)
Distance effect $\sum_j \delta_{ij}\tilde{v}_j$		4.933 (0.188)		3.439 (0.179)
		P <0.001		P <0.001
		(4.564, 5.302)		(3.087, 3.791)
Dependency effect $\sum_j w_{ij}\tilde{v}_j$			14.94 (0.211)	14.44 (0.212)
			P <0.001	P <0.001
			(14.52, 15.35)	(14.03, 14.86)
Subcategory FE	Yes	Yes	Yes	Yes
PUMA FE	Yes	Yes	Yes	Yes
Observations	40,135	40,135	40,135	40,135
R^2	0.171	0.185	0.263	0.270
Adjusted R^2	0.169	0.183	0.261	0.268

Supplementary Table 46: Linear regression models predicting the change in visits to POIs during the pandemic (2019 September - November) in Los Angeles when using dependency network generated from stays shorter than 4 hours.

	Dependent variable: $\tilde{v}_i$ *(Change in visits during the pandemic)*			
	(1)	(2)	(3)	(4)
Constant	-18.5 (4.834)	-19.0 (4.797)	-22.9 (4.544)	-23.1 (4.527)
	P <0.001	P <0.001	P <0.001	P <0.001
	(-28.0, -9.11)	(-28.4, -9.61)	(-31.8, -14.0)	(-31.9, -14.2)
Distance effect $\sum_j \delta_{ij}\tilde{v}_j$		4.905 (0.096)		3.241 (0.091)
		P <0.001		P <0.001
		(4.716, 5.094)		(3.062, 3.421)
Dependency effect $\sum_j w_{ij}\tilde{v}_j$			15.07 (0.102)	14.61 (0.103)
			P <0.001	P <0.001
			(14.87, 15.27)	(14.40, 14.81)
Subcategory FE	Yes	Yes	Yes	Yes
PUMA FE	Yes	Yes	Yes	Yes
Observations	164,195	164,195	164,195	164,195
R^2	0.139	0.152	0.239	0.245
Adjusted R^2	0.138	0.151	0.238	0.244

Supplementary Table 47: Linear regression models predicting the change in visits to POIs during the pandemic (2019 September - November) in Dallas when using dependency network generated from stays shorter than 4 hours.

	Dependent variable: $\tilde{v}_i$ *(Change in visits during the pandemic)*			
	(1)	(2)	(3)	(4)
Constant	3.572 (10.11)	0.977 (10.01)	-13.2 (9.521)	-14.2 (9.476)
	P =0.723	P =0.922	P =0.162	P =0.132
	(-16.2, 23.40)	(-18.6, 20.60)	(-31.9, 5.378)	(-32.8, 4.309)
Distance effect $\sum_j \delta_{ij}\tilde{v}_j$		5.910 (0.146)		3.843 (0.140)
		P <0.001		P <0.001
		(5.622, 6.197)		(3.568, 4.118)
Dependency effect $\sum_j w_{ij}\tilde{v}_j$			16.34 (0.161)	15.65 (0.163)
			P <0.001	P <0.001
			(16.02, 16.66)	(15.34, 15.97)
Subcategory FE	Yes	Yes	Yes	Yes
PUMA FE	Yes	Yes	Yes	Yes
Observations	78,929	78,929	78,929	78,929
R^2	0.144	0.162	0.242	0.250
Adjusted R^2	0.143	0.160	0.241	0.248

Supplementary Table 48: Linear regression models predicting the change in visits to POIs during the school semester (2019 September - November) compared to summer break (2019 June - August) in New York.

	Dependent variable: $\tilde{v}_i$ *(Change in visits during school semester)*			
	(1)	(2)	(3)	(4)
Constant	-5.92 (14.83)	-2.48 (14.70)	-4.70 (14.42)	-3.06 (14.39)
	P =0.689	P =0.865	P =0.744	P =0.831
	(-35.0, 23.15)	(-31.2, 26.33)	(-32.9, 23.56)	(-31.2, 25.14)
Distance effect $\sum_j \delta_{ij}\tilde{v}_j$		15.11 (0.240)		7.714 (0.247)
		P <0.001		P <0.001
		(14.64, 15.58)		(7.229, 8.200)
Dependency effect $\sum_j w_{ij}\tilde{v}_j$			26.13 (0.235)	23.74 (0.247)
			P <0.001	P <0.001
			(25.67, 26.59)	(23.25, 24.22)
Subcategory FE	Yes	Yes	Yes	Yes
PUMA FE	Yes	Yes	Yes	Yes
Observations	212,008	212,008	212,008	212,008
R^2	0.015	0.033	0.069	0.073
Adjusted R^2	0.014	0.032	0.068	0.072

Supplementary Table 49: Linear regression models predicting the change in visits to POIs during the school semester (2019 September - November) compared to summer break (2019 June - August) in Boston.

	Dependent variable: $\tilde{v}_i$ *(Change in visits during school semester)*			
	(1)	(2)	(3)	(4)
Constant	12.09 (16.29)<br>P =0.458<br>(-19.8, 44.02)	17.42 (15.96)<br>P =0.274<br>(-13.8, 48.72)	18.28 (15.45)<br>P =0.236<br>(-12.0, 48.58)	20.11 (15.39)<br>P =0.191<br>(-10.0, 50.29)
Distance effect $\sum_j \delta_{ij}\tilde{v}_j$		20.47 (0.456)<br>P <0.001<br>(19.57, 21.36)		9.685 (0.475)<br>P <0.001<br>(8.754, 10.61)
Dependency effect $\sum_j w_{ij}\tilde{v}_j$			33.63 (0.458)<br>P <0.001<br>(32.73, 34.53)	29.87 (0.492)<br>P <0.001<br>(28.90, 30.83)
Subcategory FE	Yes	Yes	Yes	Yes
PUMA FE	Yes	Yes	Yes	Yes
Observations	48,652	48,652	48,652	48,652
R^2	0.049	0.087	0.144	0.151
Adjusted R^2	0.046	0.084	0.141	0.148

Supplementary Table 50: Linear regression models predicting the change in visits to POIs during the school semester (2019 September - November) compared to summer break (2019 June - August) in Seattle.

	Dependent variable: $\tilde{v}_i$ *(Change in visits during school semester)*			
	(1)	(2)	(3)	(4)
Constant	-7.76 (56.04)<br>P =0.889<br>(-117., 102.0)	-8.32 (56.01)<br>P =0.881<br>(-118., 101.4)	-8.75 (55.94)<br>P =0.875<br>(-118., 100.8)	-9.05 (55.92)<br>P =0.871<br>(-118., 100.5)
Distance effect $\sum_j \delta_{ij}\tilde{v}_j$		6.630 (0.991)<br>P <0.001<br>(4.687, 8.573)		4.304 (1.011)<br>P <0.001<br>(2.321, 6.286)
Dependency effect $\sum_j w_{ij}\tilde{v}_j$			12.02 (0.974)<br>P <0.001<br>(10.11, 13.93)	11.15 (0.995)<br>P <0.001<br>(9.206, 13.10)
Subcategory FE	Yes	Yes	Yes	Yes
PUMA FE	Yes	Yes	Yes	Yes
Observations	41,294	41,294	41,294	41,294
R^2	0.004	0.005	0.008	0.008
Adjusted R^2	0.001	0.002	0.005	0.005

Supplementary Table 51: Linear regression models predicting the change in visits to POIs during the school semester (2019 September - November) compared to summer break (2019 June - August) in Los Angeles.

	Dependent variable: $\tilde{v}_i$ (Change in visits during school semester)			
	(1)	(2)	(3)	(4)
Constant	4.979 (12.90)	5.578 (12.83)	6.292 (12.63)	6.486 (12.61)
	P =0.699	P =0.663	P =0.618	P =0.607
	(-20.3, 30.27)	(-19.5, 30.72)	(-18.4, 31.05)	(-18.2, 31.21)
Distance effect $\sum_j \delta_{ij}\tilde{v}_j$		11.14 (0.253)		5.416 (0.260)
		P <0.001		P <0.001
		(10.64, 11.63)		(4.905, 5.927)
Dependency effect $\sum_j w_{ij}\tilde{v}_j$			21.40 (0.251)	19.81 (0.262)
			P <0.001	P <0.001
			(20.90, 21.89)	(19.29, 20.32)
Subcategory FE	Yes	Yes	Yes	Yes
PUMA FE	Yes	Yes	Yes	Yes
Observations	166,784	166,784	166,784	166,784
R^2	0.009	0.020	0.050	0.052
Adjusted R^2	0.008	0.019	0.049	0.051

Supplementary Table 52: Linear regression models predicting the change in visits to POIs during the school semester (2019 September - November) compared to summer break (2019 June - August) in Dallas.

	Dependent variable: $\tilde{v}_i$ (Change in visits during school semester)			
	(1)	(2)	(3)	(4)
Constant	15.96 (45.82)	32.60 (42.74)	25.89 (41.70)	33.77 (40.70)
	P =0.727	P =0.445	P =0.534	P =0.406
	(-73.8, 105.7)	(-51.1, 116.3)	(-55.8, 107.6)	(-46.0, 113.5)
Distance effect $\sum_j \delta_{ij}\tilde{v}_j$		65.77 (0.598)		40.32 (0.634)
		P <0.001		P <0.001
		(64.59, 66.94)		(39.08, 41.57)
Dependency effect $\sum_j w_{ij}\tilde{v}_j$			75.00 (0.579)	57.42 (0.629)
			P <0.001	P <0.001
			(73.86, 76.13)	(56.19, 58.66)
Subcategory FE	Yes	Yes	Yes	Yes
PUMA FE	Yes	Yes	Yes	Yes
Observations	81,103	81,103	81,103	81,103
R^2	0.006	0.135	0.177	0.216
Adjusted R^2	0.004	0.134	0.175	0.214

6 Cascading impacts of hypothetical shocks

6.1 Leontief open model

To explore what the behavior-based dependency network can tell us about other types of future shocks, we apply the network effects regression model to simulate the spatial cascades of such shocks in different cities. More specifically, re-writing and reorganizing the regression model in matrix form, we obtain

$$\vec{v} = W\vec{v} + \vec{f} \tag{10}$$

where $\vec{v}$ is a vector of $\tilde{v}_i$ for all N places, W is an $N \times N$ matrix where each element is $\tilde{w}_{ij} = \hat{\beta}_W w_{ij}$, and vector $\vec{f}$ is an aggregation of all fixed effects β_0, η_i, and θ_i. This model specification is known as the Leontief Open Model, which is a simplified and linear economic model for an economy in which input equals output [8]. To predict the propagation of shocks throughout places in the city, the shocks are modeled in the fixed effect vector f (e.g., all colleges experience an external shock of -50% visits reduction due to uptake of online education), and the production vector $\vec{v}$ is computed by solving the linear system $\hat{\vec{v}} = (I - W)^{-1}\vec{f}$ via the generalized minimal residual iteration method.

The shift to online education which occurred during the pandemic is reported to have a continuing effect, with roughly 20 percent of school systems planning to or have already started online school programs [12]. Previous studies [1] as well as analysis in Figure 2 in the main manuscript have pointed out that college campuses have a substantial impact on the local economy. If online learning and remote education were permanent and increased with the help of advanced technology (e.g., augmented reality), what impacts would it have on other businesses and amenities?

Figure 36a shows the simulated effects of a 50% reduction in visits to college POIs (gray points) on nearby non-college POIs (red points; darker red indicates larger negative impacts). Impacted POIs are not limited to those in proximity to college POIs, but also in locations that are popular with college students, for example, Massachusetts Avenue which connects MIT and Harvard University. For comparison, we simulated the shocks to non-college POIs using the physical distance network $\hat{W}$, where $\hat{w}_{ij}$ is used as the matrix elements instead of behavior-based dependency w_{ij} in Figure 36c. Comparing the simulation results using the dependency network and the null network shows that neglecting the behavior-based dependencies results in a substantial underestimation of the effects on POIs that are located further away from colleges. Model parameters, $\beta_W = 0.70$ and $\beta_{null} = 0.15$ were derived from prior regressions on estimation of changes in visits during pre-pandemic periods.

The effects of online education were heterogeneous for different place categories located at different distances from colleges. Figure 36b shows the 90th percentile of impacts on POIs by category and distance (log scaled). While most significant impacts occur within 0.5 km, places such as arts and museums, food, and service places experience substantial long-distance impacts. Furthermore, simulations assuming different levels of visit decrease to colleges (e.g., -100%, -25%) show a similar long-distance cascade of shocks (Figures 37 and 38). These persistent spatial cascades emphasize the importance of considering behavior-based dependency relationships between places to grasp the holistic impact of such urban shocks for resilient urban planning.

6.2 Cascading effects of individual place closures

Further leveraging the network model, we are able to simulate the impacts of POI closure scenarios and identify the seed nodes (POIs) that have the largest cascading effects on other POIs if inflicted by other urban shocks. For each node, we simulate the cascading impacts of a 100% visit change to a

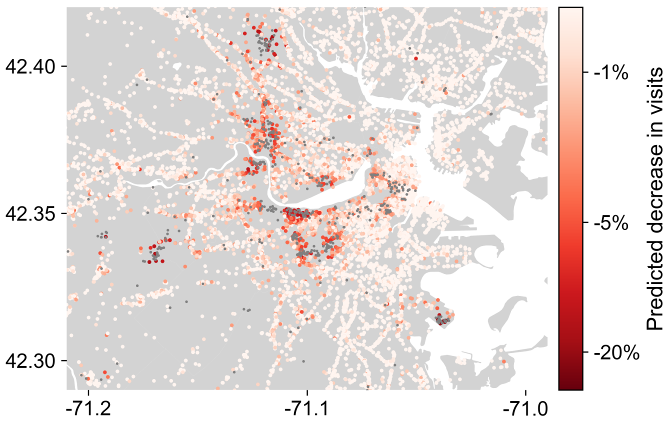

(a) Map of effects using dependency network

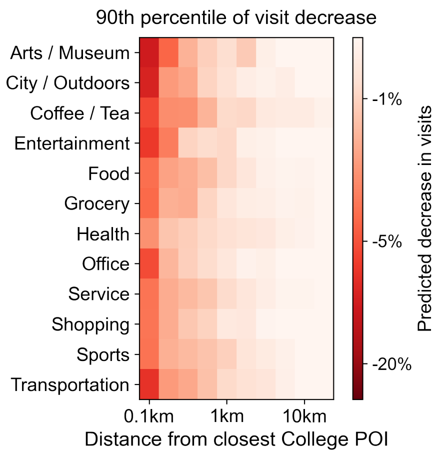

(b) Category and distance effects using dependency network

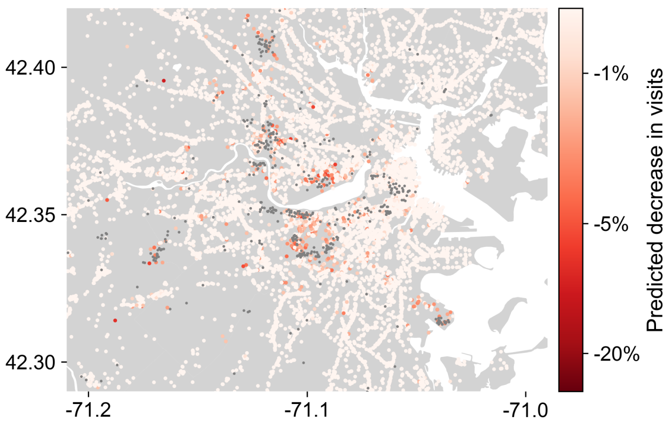

(c) Map of effects using null network

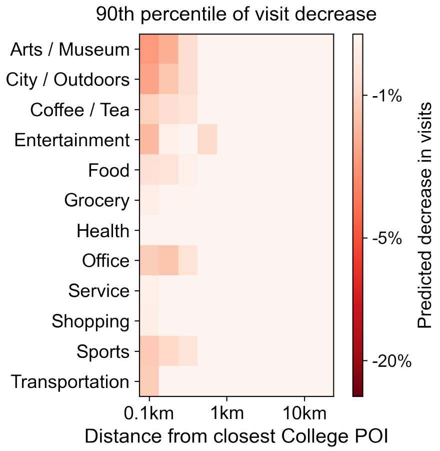

(d) Category and distance effects using null network

Supplementary Figure 36: Cascading impacts of a 50% visit reduction to colleges. a. Simulated effects of a 50% reduction in visits to college POIs (gray points) on nearby non-college POIs (red points; darker red indicates larger negative impacts), using the fitted Leontief Open Model. Impacted POIs are not limited to those in proximity to college POIs. b. Impacts of the 50% visit reduction to colleges on places by category and distance (90th percentile decrease in visits are shown, log scaled). c. and d. show results using the null networks. Maps were produced in Python using the TIGER shapefiles from the U.S. Census Bureau [15].

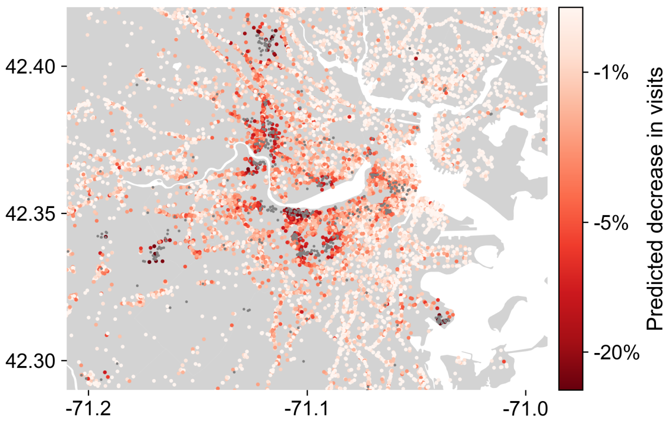

(a) Map of effects using dependency network

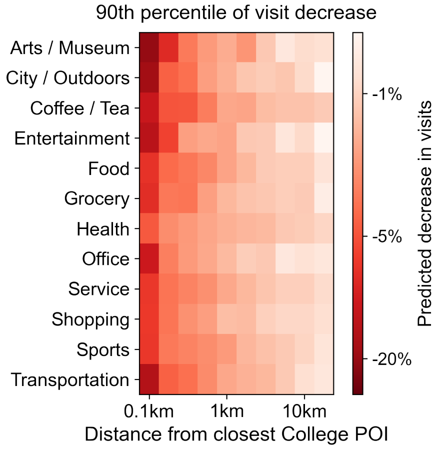

(b) Category and distance effects using dependency network

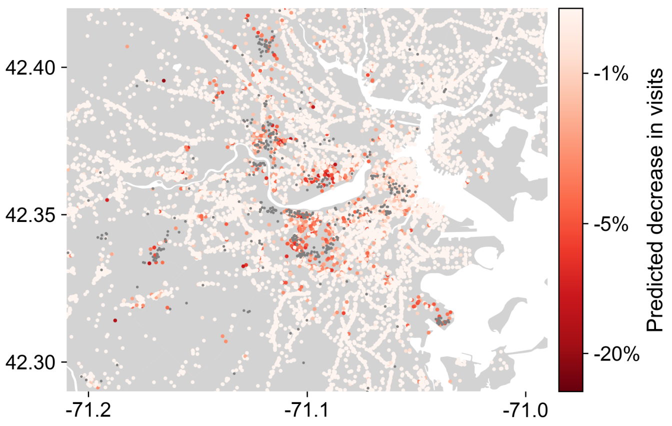

(c) Map of effects using null network

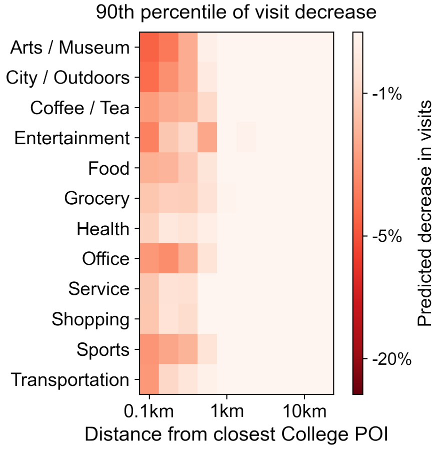

(d) Category and distance effects using null network

Supplementary Figure 37: Cascading impacts of a 100% visit reduction to colleges. a. Simulated effects of a 50% reduction in visits to college POIs (gray points) on nearby non-college POIs (red points; darker red indicates larger negative impacts), using the fitted Leontief Open Model. Impacted POIs are not limited to those in proximity to college POIs. b. Impacts of the 100% visit reduction to colleges on places by category and distance (90th percentile decrease in visits are shown, log scaled). c. and d. show results using the null networks. Maps were produced in Python using the TIGER shapefiles from the U.S. Census Bureau [15].

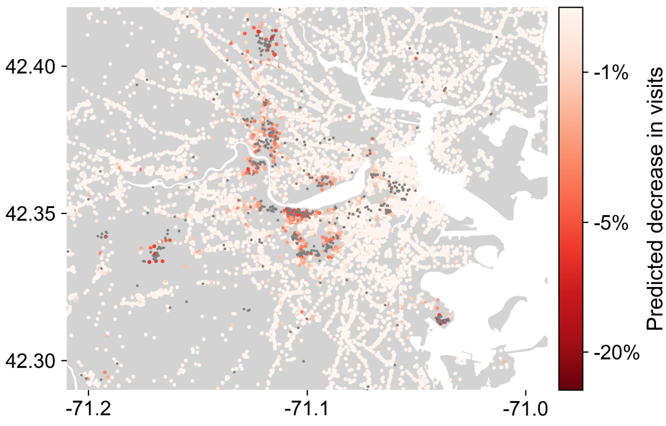

(a) Map of effects using dependency network

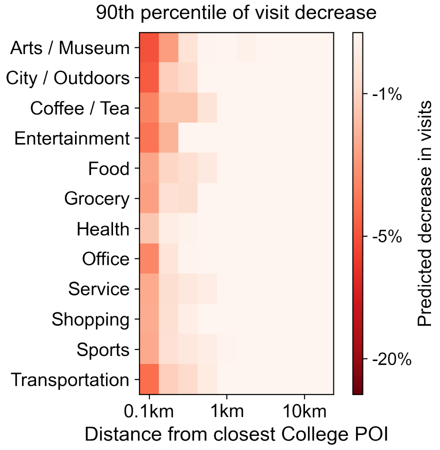

(b) Category and distance effects using dependency network

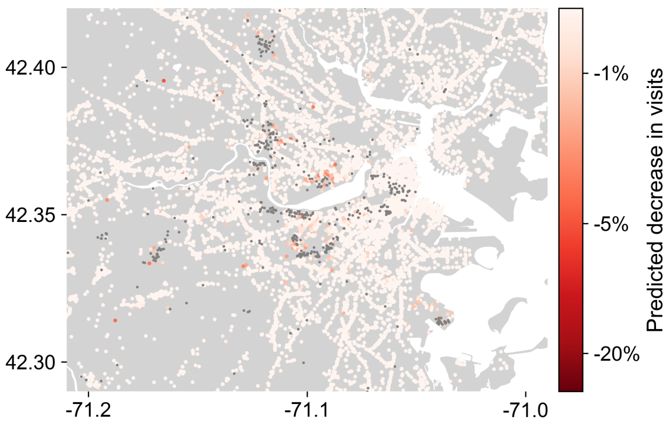

(c) Map of effects using null network

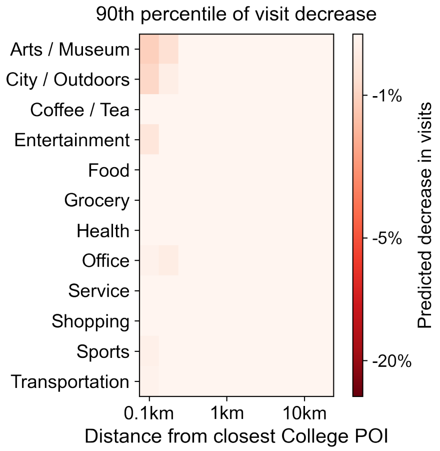

(d) Category and distance effects using null network

Supplementary Figure 38: Cascading impacts of a 25% visit reduction to colleges. a. Simulated effects of a 25% reduction in visits to college POIs (gray points) on nearby non-college POIs (red points; darker red indicates larger negative impacts), using the fitted Leontief Open Model. Impacted POIs are not limited to those in proximity to college POIs. b. Impacts of the 50% visit reduction to colleges on places by category and distance (90th percentile decrease in visits are shown, log scaled). c. and d. show results using the null networks. Maps were produced in Python using the TIGER shapefiles from the U.S. Census Bureau [15].

single node i, by computing $\hat{\vec{\nu}}^{(i)} = (I - W)^{-1}\vec{e}^{(i)}$, where $\vec{e}^{(i)}$ is a one-hot encoding vector of the initial shock that assigns a change in visits of $+1$ to node i and 0 otherwise, and $\hat{\vec{\nu}}^{(i)}$ is the resulting vector of the cascading impacts, where each element measures the impacts of the initial shock to all nodes. The total impacts of changes in the number of visits to all nodes can be computed by multiplying $\hat{\vec{\nu}}^{(i)} = (\hat{\nu}_1^{(i)}, \ldots, \hat{\nu}_N^{(i)})$ with the vector of total visits to each POI, $\vec{n} = (n_1, \cdots, n_N)$. Thus, the total impacts of the initial shock to node i can be computed by $C_i = \sum_{j;j \neq i} \hat{\nu}_j^{(i)} n_j$. By further scaling the impact to its own size n_i, we obtain the total relative cascading effect as $\hat{C}_i = C_i / n_i$. $\hat{C}_i = 0.3$ indicates that increasing the number of visits to node i by 100% $(= n_i)$ results in a total of $30\% * n_i$ increase in visits across all other nodes. The mean relative cascading impacts of each POI category, $\hat{C}_{category}$ are shown in the y-axis of Figures 39 to 41. POI categories such as airports, supercenters, colleges, furniture stores, theme parks, railway stations, and sports stadiums have a high impact on other POIs in urban areas propagated through behavior-based dependency networks.

When implementing policies to close down certain POIs for emergency response (e.g., lockdowns during pandemics), it is important to understand the spatial extent of the cascade. To quantify this, we defined the distance range of the cascade by computing the average distance to impacted nodes, weighted by the magnitude of the impacts. More specifically, we compute the weighted distance range of POI i by $\hat{d}_i = \sum_{j;j \neq i} \hat{\nu}_j^{(i)} d_{ij} / \sum_{j;j \neq i} \hat{\nu}_j^{(i)}$.

The weighted distance range of impact for each POI category, $\hat{d}_{category}$ are shown in the x-axis of Figures 39 to 41. Supercenters and colleges have high cascading effects but are focused locally ($\sim$ 1.5km around the POI). On the other hand, the impacts of airports, stadiums, theme parks, and gas stations are both large and far-reaching ($\sim$ 2.5km to 3.5km). Understanding the magnitude and spatial extent of the cascading effects could be applied to design emergency management policies to effectively close places while minimizing economic losses. The large magnitude of the spatial cascades that occur due to behavior-based dependency networks calls for new urban policy-making approaches that balance the benefits of mobility restriction measures (e.g., preventing the spread of diseases) while minimizing the total cascading economic impacts to urban places and amenities.

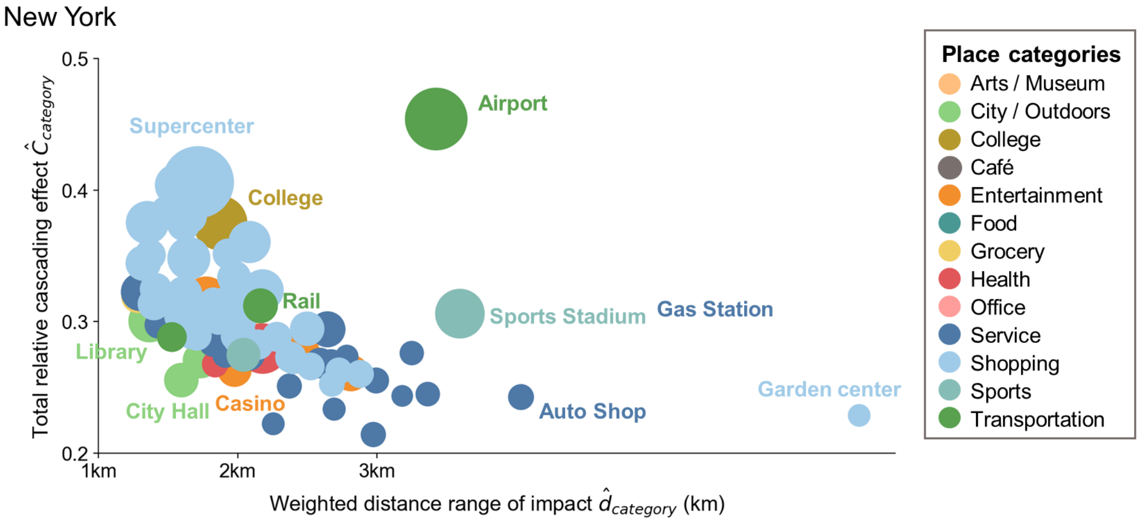

(a) New York

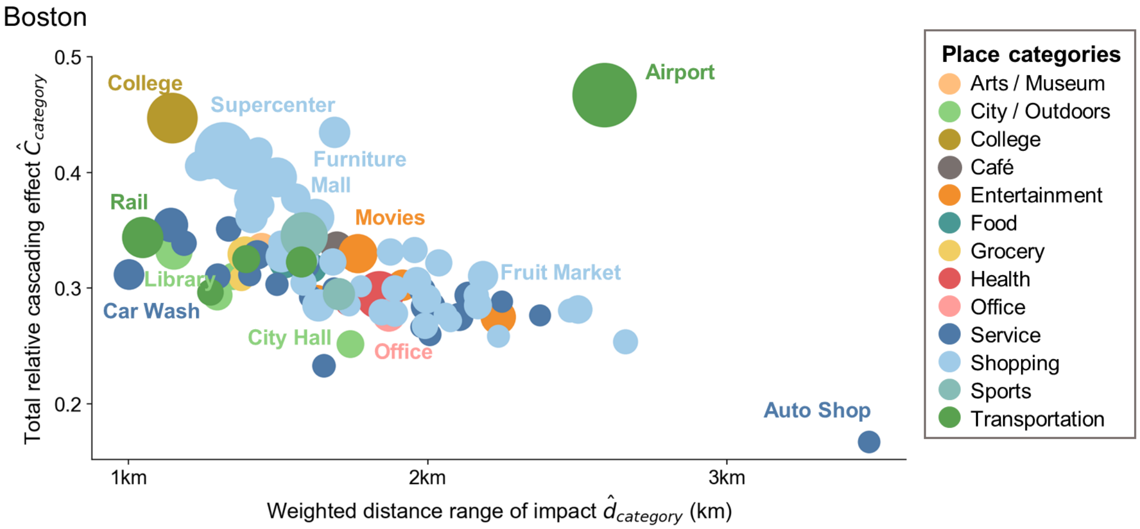

(b) Boston

Supplementary Figure 39: Total cascading impact of closing places on other locations. Total cascading impact of closing places on other locations, relative to its own size (x-axis) and the weighted distance range of the impact (y-axis) for different POI subcategories in New York and Boston.

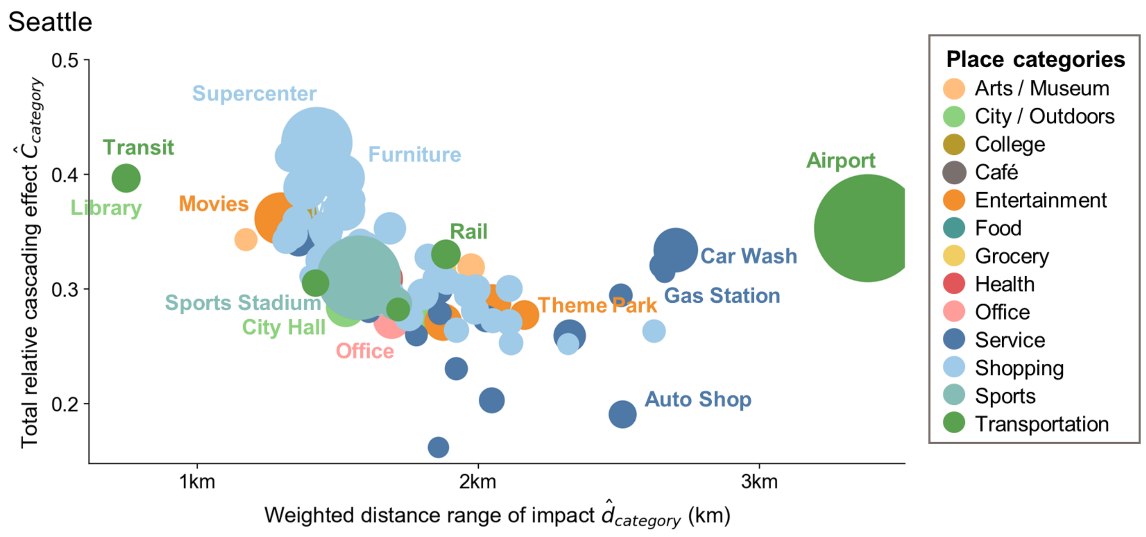

(a) Seattle

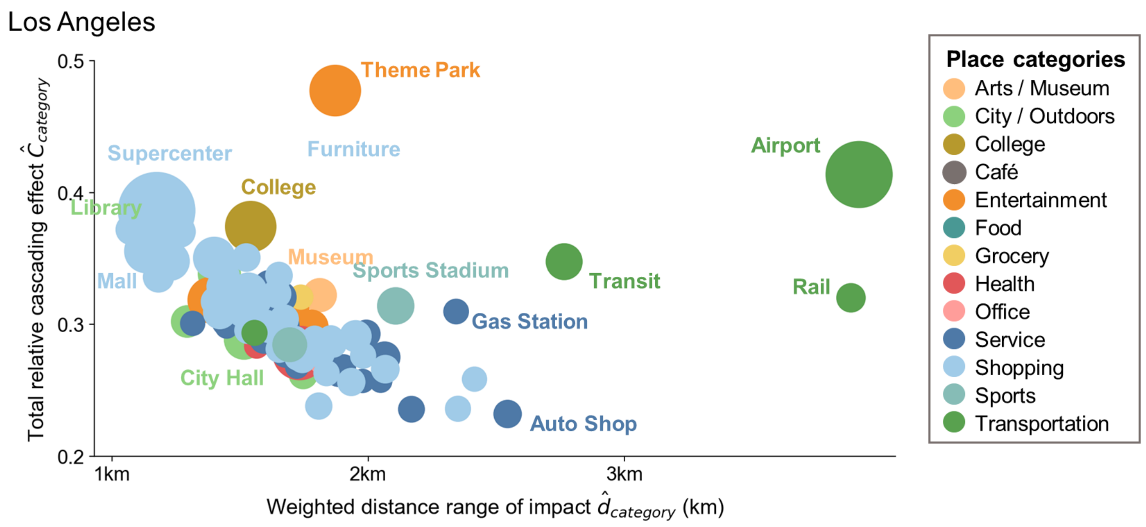

(b) Los Angeles

Supplementary Figure 40: Total cascading impact of closing places on other locations. Total cascading impact of closing places on other locations, relative to its own size (x-axis) and the weighted distance range of the impact (y-axis) for different POI subcategories in Seattle and Los Angeles.

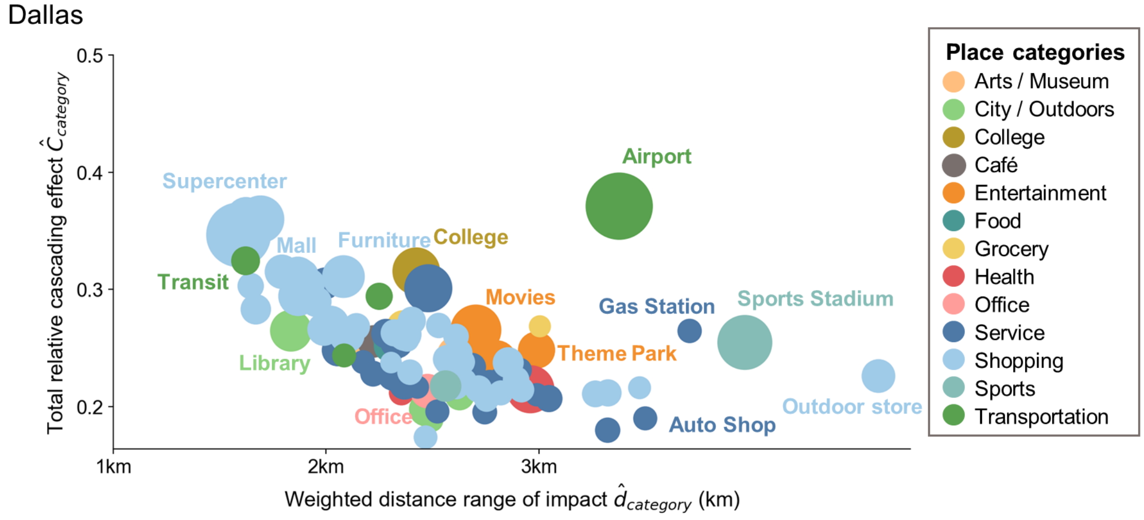

(a) Dallas

Supplementary Figure 41: Total cascading impact of closing places on other locations. Total cascading impact of closing places on other locations, relative to its own size (x-axis) and the weighted distance range of the impact (y-axis) for different POI subcategories in Dallas.

7 Software

Analysis was conducted using Python, Jupyter Lab, and the following libraries and software:

- `NumPy` [4] for general computation on Python.
- `Pandas` [9] for loading, transforming, and analyzing data tables.
- `Matplotlib` [5] for creating plots and figures.
- `GeoPandas` [7] for spatial analysis and plotting map figures.
- `networkx` [3] for network analysis.
- `Statsmodels` [13] for statistical modeling and econometric analysis.
- A Python implementation of the R `Stargazer` multiple regression model creation tool[5] was used to create the regression tables.

[5] `https://github.com/mwburke/stargazer`

References

[1] Ernest R Bonner. The economic impact of a university on its local community. *Journal of the American Institute of Planners*, 34(5):339–343, 1968.

[2] United States Census Bureau. American community survey. `https://www.census.gov/programs-surveys/acs`. Accessed: 2019-12-14.

[3] Aric A. Hagberg, Daniel A. Schult, and Pieter J. Swart. Exploring network structure, dynamics, and function using networkx. In Gaël Varoquaux, Travis Vaught, and Jarrod Millman, editors, *Proceedings of the 7th Python in Science Conference*, pages 11 – 15, Pasadena, CA USA, 2008.

[4] Charles R Harris, K Jarrod Millman, Stéfan J Van Der Walt, Ralf Gommers, Pauli Virtanen, David Cournapeau, Eric Wieser, Julian Taylor, Sebastian Berg, Nathaniel J Smith, et al. Array programming with numpy. *Nature*, 585(7825):357–362, 2020.

[5] John D Hunter. Matplotlib: A 2d graphics environment. *Computing in science & engineering*, 9(03):90–95, 2007.

[6] Shan Jiang, Yingxiang Yang, Siddharth Gupta, Daniele Veneziano, Shounak Athavale, and Marta C González. The timegeo modeling framework for urban mobility without travel surveys. *Proceedings of the National Academy of Sciences*, 113(37):E5370–E5378, 2016.

[7] K Jordahl. Geopandas: Python tools for geographic data. *URL: https://github.com/geopandas/geopandas*, 3, 2014.

[8] Wassily Leontief. *Input-output economics*. Oxford University Press, 1986.

[9] Wes McKinney et al. pandas: a foundational python library for data analysis and statistics. *Python for high performance and scientific computing*, 14(9):1–9, 2011.

[10] Esteban Moro, Dan Calacci, Xiaowen Dong, and Alex Pentland. Mobility patterns are associated with experienced income segregation in large us cities. *Nature Communications*, 12(1):1–10, 2021.

[11] Matthew J Salganik. *Bit by bit: Social research in the digital age*. Princeton University Press, 2019.

[12] Heather L. Schwartz, David Grant, Melissa Kay Diliberti, Gerald P. Hunter, and Claude Messan Setodji. *Remote Learning Is Here to Stay: Results from the First American School District Panel Survey*. RAND Corporation, Santa Monica, CA, 2020.

[13] Skipper Seabold and Josef Perktold. Statsmodels: Econometric and statistical modeling with python. In *Proceedings of the 9th Python in Science Conference*, volume 57, page 61. Austin, TX, 2010.

[14] Eric Tsetsi and Stephen A Rains. Smartphone internet access and use: Extending the digital divide and usage gap. *Mobile Media & Communication*, 5(3):239–255, 2017.

[15] United States Census Bureau. Tiger data products guide. `https://www.census.gov/programs-surveys/geography/guidance/tiger-data-products-guide.html`. Accessed: 2023-02-27.

[16] Qi Wang, Nolan Edward Phillips, Mario L Small, and Robert J Sampson. Urban mobility and neighborhood isolation in america's 50 largest cities. *Proceedings of the National Academy of Sciences*, 115(30):7735–7740, 2018.

[17] Longgang Xiang, Meng Gao, and Tao Wu. Extracting stops from noisy trajectories: A sequence oriented clustering approach. *ISPRS International Journal of Geo-Information*, 5(3):29, 2016.

[18] Takahiro Yabe, Bernardo García Bulle Bueno, Xiaowen Dong, Alex Pentland, and Esteban Moro. Behavioral changes during the covid-19 pandemic decreased income diversity of urban encounters. *Nature Communications*, 14(1):2310, 2023.
